# Health and economic impact of delaying large-scale HPV vaccination and screening implementation on cervical cancer in China: a modelling study

**DOI:** 10.1016/j.lanwpc.2023.100768

**Published:** 2023-04-20

**Authors:** Meng Gao, Shangying Hu, Xuelian Zhao, Tingting You, Mark Jit, Yang Liu, Youlin Qiao, Fanghui Zhao, Chen Wang

**Affiliations:** a School of Population Medicine and Public Health, Chinese Academy of Medical Sciences & Peking Union Medical College, Beijing, China; b Department of Cancer Epidemiology, National Cancer Center/National Clinical Research Center for Cancer/Cancer Hospital, Chinese Academy of Medical Sciences and Peking Union Medical College, Beijing, China; c Department of Infectious Disease Epidemiology, Faculty of Epidemiology and Population Health, London School of Hygiene & Tropical Medicine, London, United Kingdom; d School of Public Health, University of Hong Kong, Hong Kong, Hong Kong SAR, China

**Keywords:** Delay, Human papillomavirus vaccination, Cervical screening, Economic evaluation, Elimination

## Abstract

**Background:**

Current uptake of HPV vaccination and screening in China is far below World Health Organization 2030 targets for cervical cancer elimination. We quantified health and economic losses of delaying large-scale HPV vaccination and screening implementation in China.

**Methods:**

We used a previously validated transmission model to project lifetime health benefits, costs, effectiveness, and timeline for cervical cancer elimination of alternative scenarios, including combining HPV vaccination initiated from 2022 to 2030 with screening in different modalities and coverage increase rates, as well as screening alone. All women living or projected to be born in China during 2022–2100 were considered. We employed a societal perspective.

**Findings:**

Regardless of vaccine type, immediate large-scale vaccination initiated in 2022 and achieving 70% coverage of HPV-based screening in 2030 (no-delay scenario) would be the least costly and most effective. Compared with the no-delay scenario, delaying vaccination by eight years would result in 434,000–543,000 additional cervical cancer cases, 138,000–178,000 deaths, and $2863–4437 million costs, and delay elimination by 9–10 years. Even with immediate vaccination, the gradual scale-up of LBC-based screening to 70% coverage in 2070 would result in 2,530,000–3,060,000 additional cases, 909,000–1,040,000 deaths, and $5098–5714 million costs compared with no-delay scenario, and could not achieve elimination if domestic 2vHPV or 4vHPV vaccines are used (4.09–4.21 cases per 100,000 woman in 2100).

**Interpretation:**

Delaying large-scale HPV vaccination and/or high-performance screening implementation has detrimental consequences for cervical cancer morbidity, mortality, and expenditure. These findings should spur health authorities to expedite large-scale vaccine rollout and improve screening.

**Funding:**

10.13039/100000865Bill & Melinda Gates Foundation (INV-031449 and INV-003174) and 10.13039/501100003345CAMS Innovation Fund for Medical Sciences (CIFMS) (2021-I2M-1-004).

**Research in context**

**Evidence before this study**

Human papillomavirus (HPV) vaccination and high-performance cervical screening are recommended by the World Health Organization (WHO) for women in all countries, but have seen slow uptake in many low- and middle-income countries. China accounts for one-fifth of the world's cervical cancer burden, but the vaccination and screening coverage is far below the WHO 2030 targets for cervical cancer elimination. Implementation of large-scale centrally-funded vaccination and screening programs, where the government can negotiate lower prices for vaccines and detection tests and then provide them for free to the target population, is able to effectively increase coverage and allow for the interventions to be more cost-effective. However, the health and economic cost of delaying implementation of such programs for China is still unclear. We searched PubMed and China National Knowledge Infrastructure, without language restrictions, for studies published from Jan 1, 2000, to October 31, 2022, with the search terms “cervical cancer”, “delay”, “HPV vaccine” or “screening”, and “cost-effectiveness” or “timing” or “timeline” or “elimination”. Three studies were identified. One study from China estimated that if girls aged 9–15 years (seven birth cohorts) in 2006 were left unvaccinated due to the delayed program, approximately 381,000 preventable cervical cancer cases and 212,000 related deaths could occur in these cohorts. One global study estimated that a ten-year (2020–2029) HPV vaccination among 15-year-old girls could prevent 622,000 cervical cancer cases in China. Both studies suggest delay in national HPV vaccination could result in excess morbidity and mortality. However, neither study considered the herd effects of vaccination, which could further enhance its benefits, nor the effects of future changes in screening coverage and performance. The third study evaluated cases and deaths caused by delayed screening with visual inspection with acetic acid (VIA) or HPV testing in India, but it did not consider cytological screening widely used in China. None of these three studies quantified the economic impact of the delay to large-scale vaccination and screening programs, which is needed for policymakers to understand the value of early initiation of the programs.

**Added value of this study**

This analysis incorporates a transmission dynamic model to assess the lifetime health benefits, costs, and timeline to cervical cancer elimination of alternative scenarios combining vaccination initiated in different years or never initiated, with screening in different modalities and coverage increase rates. Our study demonstrated that the no-delay scenario with immediate national vaccination initiated in 2022 and rapid scale-up of HPV-based screening was both cost-saving and health-improving and thus dominated all alternatives. This scenario would not only allow China to eliminate cervical cancer by 2059–2063 but also save $21.7–27.7 billion costs in China compared to the status quo. Compared with the no-delay scenario, any of the three scenarios respectively involving short delays to the initiation of national vaccination, no switch of screening method to high-performance HPV testing, and slow increases in screening coverage, would result in increased disease burden and net costs, as well as delay the timeline to cervical cancer elimination. Notably, the negative health and economic impacts of delayed vaccination were larger under scenarios where screening uptake and modalities were suboptimal.

**Implications of all the available evidence**

Our findings highlight the importance of early implementation of large-scale HPV vaccination and HPV-based screening. Incorporating national HPV vaccination and cervical screening into a comprehensive cancer prevention and control program is necessary to attain national elimination of cervical cancer. These estimates strengthen the evidence base for the government's commitment to expediting national vaccine rollout and screening improvement. Our findings also emphasise the urgency of initiating a national HPV program while cervical screening is still suboptimal or gradually being improved. This may provide evidence for decisions around HPV vaccination in other countries where screening programs are limited.

## Introduction

Cervical cancer is one of the leading causes of cancer incidence and mortality among women worldwide.^1^ Human papillomavirus (HPV) vaccines are highly effective against persistent infection with HPV types that cause the majority of cervical cancer cases,^2^ while timely screening can detect precancerous lesions and enable treatment to prevent their progression to cancer. The World Health Organization (WHO) has developed a global strategy toward the elimination of cervical cancer, proposing a 2030 target of 90% of girls being vaccinated by age 15 and 70% of women being screened with a high-performance test.^3^ By 2020, more than 85% of high-income and 55% of upper-middle-income countries had introduced the HPV vaccine into their national immunisation programs (NIP).^3^ Some high-income countries, like Australia and the United States, have dramatically reduced cervical cancer incidence through population-wide screening programs, so combined with high uptake of vaccination are on track to eliminate cervical cancer in one to two decades.^4^^,^^5^ Nonetheless, in many low- and middle-income countries, national vaccination and screening programs are being implemented more slowly, and morbidity and mortality due to cervical cancer continue to increase, leading to widening disparities.

Approximately 18% of the global burden of cervical cancer in 2020 occurred in China,^6^ and its incidence in China increased by 8.5% per year during 2000–2016.^7^ However, China has not yet introduced the HPV vaccine into its NIP.^8^ As a result, HPV vaccines are only available on the private market and the prices are high, leading to current relatively low HPV vaccine coverage.^9^ For WHO's recommended primary target population (girls aged 9–14),^10^ vaccination coverage is less than 1%.^11^ If a large-scale vaccination program is implemented, the vaccines will be purchased by the government and provided for free to target population, and thus the vaccine uptake is likely to increase. Furthermore, although government-funded screening has been available in China since 2009, population screening coverage is only 21.4%.^12^ A national well-organised screening system for cervical cancer has not been established.

The current delay in large-scale vaccination and screening programs is expected to increase morbidity and delay cervical cancer elimination.^13^, ^14^, ^15^ To date, only two studies have estimated the cases in vaccine-eligible girls that could be prevented by large-scale vaccination programs in China.^13^^,^^15^ However, without using dynamic models, these two studies were not able to quantify any indirect effects on unvaccinated women or the effects of future changes in screening coverage and performance. More importantly, to our knowledge, no study has evaluated the economic impacts associated with HPV vaccination and screening delays in China. Since vaccination and screening programs require investment of scarce healthcare resources, policymakers need to understand both the expected health and economic benefits of the program. Evaluation of the health and economic impacts of the delay provides evidence to health authorities about the urgency of initiating and scaling up population-wide vaccination and screening programs and reinforces their confidence in investment.

We aimed to quantify the health and economic impacts of delayed versus immediate large-scale HPV vaccination and screening implementation in China in terms of cervical cancer cases and deaths, time to cervical cancer elimination, costs, and cost-effectiveness using a dynamic model.

## Methods

The results were reported following the HPV-FRAME checklist and CHEERS checklist.

In this modelling study, we estimated lifetime health and economic outcomes of several HPV vaccination and screening scenarios under projected aging, urbanisation, and sexual activity trends. All individuals were simulated until the end of their lives (aged 85). We considered all women living or projected to be born in China during 2022–2100, including birth cohorts born between 1937 (the cohort aged 85 in 2022) and 2100. The population was stratified by area of residence (urban and rural), gender, sexual activity (high, low, none), and age group (0–84 per year, and ≥85). The proportion of the population in each residence area and sexual activity category was assumed to change over time according to projected trends (appendix p6).

### Model

We used a hybrid model consisting of a dynamic model and a natural history model to estimate the health outcomes and costs of different scenarios. The details of the model have been presented previously^16^ and are summarised in the appendix (pp 2–3 and Fig. S1). Briefly, the deterministic age-structured compartmental dynamic model was built to simulate HPV transmission between males and females and to project the number of HPV infections. The natural history model was then used to simulate the natural history of cervical cancer and its precursors to estimate the number of cervical cancer cases and deaths. The model has been previously calibrated using Chinese epidemiological data on high-risk HPV type prevalence, cervical cancer incidence and mortality in 2015, and HPV type distribution in women with normal cervical cytology, low-grade cervical precancerous lesions, high-grade cervical precancerous lesions, and invasive cervical cancer.^16^

### Inputs and assumptions

We used parameters obtained from published literature and government-released online datasets (Table S1). Sources of demographic and epidemiological data are shown in the appendix (p 6). Four types of vaccines licensed in China were included in our analysis: domestically manufactured 2vHPV (Cecolin®), as well as imported 2vHPV (Cervarix®), 4vHPV (Gardasil®), and 9vHPV vaccines (Gardasil-9®). We assumed that infection-acquired immunity would wane over time at a rate calibrated to Chinese data (Table S1), whereas vaccine-acquired immunity would be lifelong.^16^

For the base-case analyses, HPV vaccination was assumed to provide full protection against vaccine-target HPV types. For the imported 2vHPV vaccine, we assumed 77.1%, 43.1%, and 79.0% cross-protection against HPV types 31, 33, and 45, and for the 4vHPV vaccine, we assumed a 46.2% cross-protection against HPV type 31 according to the findings of a meta-analysis.^17^ For the domestic 2vHPV vaccine, we assumed the same cross-protection as the 4vHPV vaccine, because both vaccines were formulated with aluminum adjuvant and clinical trials of the domestic 2vHPV vaccine suggested some cross-protection against non-vaccine types of HPV.^2^

We derived utility values that were specific to patient health and treatment states from our hospital-based prospective study in China^18^ and data published elsewhere.^19^ The costs of each scenario included the direct and indirect costs that occurred in vaccination, screening, and treatment. The 4vHPV vaccine was assumed to cost $10.48 per dose based on the price paid by the Pan American Health Organization (PAHO) Revolving Fund.^20^ Domestic 2vHPV, imported 2vHPV, and 9vHPV vaccines were estimated to cost $4.32, $7.62, and $17.05 per dose, respectively, using the 4vHPV vaccine price as reference and applying the ratio of prices for each vaccine in the private market in China. Vaccine service costs were set to $4.12 per dose.^21^ Cervical screening costs using liquid-based cytology (LBC) and HPV DNA testing were estimated at $10.38 and $16.29 per screen in urban areas and $7.54 and $13.98 per screen in rural areas, respectively.^16^^,^^22^ The treatment costs for cervical intraepithelial neoplasia (CIN) and cervical cancer were collected in our nationwide multicenter cross-sectional, hospital-based survey conducted from August 2020 to June 2021. Detailed information on the cost valuation was available in the appendix (p 3) and previous study.^16^^,^^22^ All unit costs were converted to 2021 US dollars using China's Consumer Price Index for health care and average US dollar to Chinese Yuan exchange rate in 2021 (1.00 US dollar = 6.5 Chinese yuan).

### Alternative scenarios

In the “status quo scenario”, we assumed the current vaccination (no vaccination) and screening (3-yearly cytology-based screening with coverage of 26.6% in urban areas and 19.3% in rural areas^12^) situation in China was maintained. Current age-specific screening coverage is reported in Fig. S2. 70 alternative scenarios were assessed in our study, consisting of the combination of 10 vaccination initiation scenarios with seven screening scenarios (Table S2). We included alternative scenarios for the initiation year of a large-scale HPV vaccination program (ranging from 2022 to 2030), as well as a scenario where a large-scale program is never initiated. Scenarios starting in 2023–2030 imply one- to eight-year delays compared with starting in 2022. HPV vaccination was assumed to be delivered via a two-dose routine program among 12-year-old girls at 90% coverage.^3^

In contrast to vaccination, which in pilot programs was shown to be possible to scale up to 90% coverage rapidly within one year,^23^ screening may take longer to scale up for lack of a well-organised national screening system.^8^ Therefore, to reflect uncertainty around future uptake and modalities of screening, we included seven alternative screening scenarios which either maintained the current program or improved the program over time. Six improved scenarios included increases in coverage to 70% for the target population of women aged 35–64 years (based on the WHO 2030 target), with the scenarios differing by time taken to reach 70% coverage and whether there was a switch to HPV testing. We also assumed that screening coverage would continue to increase after the WHO 2030 target is reached, until reaching 90%; for example, the coverage in the United States already exceeds 70%.^24^ The “HPV 2030”, “HPV 2050”, and “HPV 2070” screening scenarios represented switching to HPV-based screening at 5-year intervals in 2022, with linearly increasing age-specific uptake from status quo in 2021, to 70% in 2030 (rapid), 2050 (moderate), and 2070 (gradual), respectively, followed by a 1% increase every year till 90% is reached. “LBC 2030”, “LBC 2050”, and “LBC 2070” screening scenarios represented maintaining LBC-based screening at 3-year intervals, with the corresponding rapid, moderate, and gradual increase in age-specific uptake (appendix pp 6–7 and Fig. S3). Screening coverage for the non-targeted population at ages 21–34 and over 65 years was assumed to remain the same as the status quo in all scenarios.

In these intervention scenarios, the most optimistic scenario was defined as the “no-delay scenario”, where vaccination was initiated in 2022 and screening switched to HPV testing in 2022, screening coverage linearly increasing to 70% by 2030, followed by a 1% increase every year till 90% is reached.

### Main analysis

For health outcomes, we estimated the cervical cancer cases and deaths averted for each intervention scenario compared with the status quo scenario. We also estimated the excess cases and deaths caused by delayed vaccination and gradually improved screening scenarios compared with the no-delay scenario. We measured the indirect effects of the vaccination scenarios by examining the excess disease burden that occurred in cohorts that missed out on vaccination due to the delayed program, as well as the burden that occurred in other cohorts. Additionally, we estimated age-standardised annual cervical incidence during 2022–2100 and the year in which cervical cancer is eliminated using Segi's world standard population. Elimination was defined using the WHO definition^3^ as the first year when the age-standardised incidence is below 4/100,000 women.

For the cost-effectiveness analysis, we estimated the incremental costs and health outcomes of each intervention scenario compared with the status quo scenario. We also estimated the excess costs and health outcomes for delayed vaccination and gradually improved screening scenarios compared with no-delay scenario. The costs associated with each scenario were estimated from a societal perspective, including direct costs and indirect costs, with separate costs associated with vaccination, screening, CIN treatment, and invasive cervical cancer treatment. The health impact of each scenario was evaluated using quality-adjusted life-years (QALYs), taking into account health state utility weights. The time horizon of the analysis was 2022–2100, and all cohorts born during those years were followed up until the end of their lives. Both costs and QALYs were discounted to 2021 at an annual rate of 3%. Incremental cost-effectiveness ratios (ICERs) were calculated as incremental cost per additional QALY gained of a scenario compared to the next most costly non-dominated scenario, to identify the optimal scenario. We applied the Chinese gross domestic product (GDP) per capita in 2021 ($12,458) as the cost-effectiveness threshold. All analyses were performed in R software (version 4.2.0).

### Sensitivity analysis

The actual price paid for vaccines at the regional or country level is often not available due to the confidential tender negotiations.^25^ We varied vaccine prices from base case to current market prices in the cost-effectiveness analysis. In one-way deterministic sensitivity analyses, we varied key parameters in the model over their plausible ranges to quantify the impact of uncertainty in individual input parameters on the results. Parameters altered consisted of the vaccine efficacy against target types; vaccine cross-protection; the achievable coverage of vaccination; the maximum achievable coverage of screening; screening sensitivity; precancerous lesions management; treatment efficacy; the cost of the vaccine, screening, and treatment; the discount rate; utilities; and the projected trends in fertility and urbanisation (Table S1). Probabilistic sensitivity analysis was conducted by performing 1000 Monte Carlo simulations to sample parameter values from their distributions, including vaccine efficacy, screening sensitivity, follow-up and treatment, costs, and utilities assumptions, and to estimate outcomes. The results of the probabilistic sensitivity analysis were used to calculate the 10th and 90th percentiles (80% uncertainty interval) of model results.

### Role of the funding source

The funder of the study had no role in study design, data collection, data analysis

## Results

**Fig. 1:**
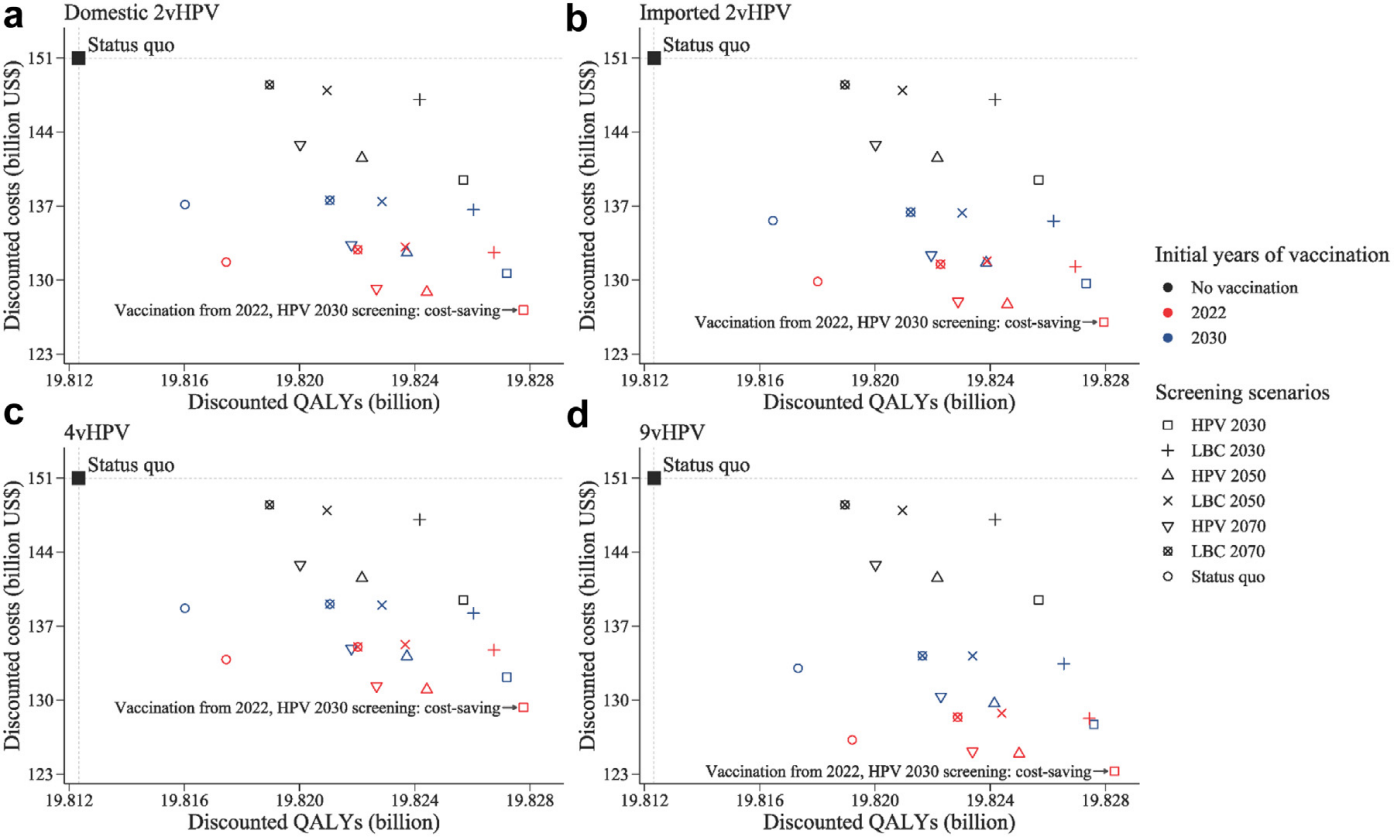
**Cost-effectiveness for different scenarios compared with status quo scenario, with 3% discounting.** (a) Domestic 2vHPV; (b) imported 2vHPV; (c) 4vHPV; and (d) 9vHPV vaccines. The black squares indicate the reference scenario, which is the status quo scenario with no vaccination and status quo screening. The scenarios in the upper left quadrant are dominated by the scenarios in the lower right quadrant. The labels represent the vaccination initiation years and the screening scenarios of the dominant scenarios. Because the dominant scenarios are cost-saving, the ICERs are negative and not shown. Different shaped points indicate seven screening scenarios with different modalities and rates of increase in coverage for target population of women aged 35–64 years. “HPV 2030”, “HPV 2050”, and “HPV 2070” screening scenarios represent switching to HPV-based screening at 5-year intervals in 2022, with linearly increasing age-specific uptake from status quo in 2021, to 70% in 2030 (rapid), 2050 (moderate), and 2070 (gradual), respectively, followed by a 1% increase every year till 90% is reached. “LBC 2030”, “LBC 2050”, and “LBC 2070” screening scenarios represent maintaining LBC-based screening at 3-year intervals, with the corresponding rapid, moderate, and gradual increase in age-specific uptake. Status quo represents maintaining LBC-based screening with current coverage. HPV, human papillomavirus; LBC, liquid-based cytology; QALY, quality-adjusted life-years; ICER, incremental cost-effectiveness ratio.

Overall, in the base case, all vaccination scenarios with vaccination initiation dates ranging from 2022 to 2030 combined with different scale-up screening scenarios were cost-saving compared with status quo scenario. Regardless of vaccine type, immediate vaccination initiated in 2022 and rapid scale-up of HPV-based screening to achieve 70% coverage in 2030 (i.e., the no-delay scenario) was both the least costly and the most effective and thus dominated all alternatives (Fig. 1). Compared with the status quo scenario, the no-delay scenario would avert 14,800,000–15,800,000 cervical cancer cases and 5,750,000–6,110,000 deaths, save $21,700–27,700 million net costs, and gain 15,400,000–16,000,000 QALYs over the lifetime of people who lived in 2022–2100, depending on the vaccine type (Tables S3–S9).

**Fig. 2:**
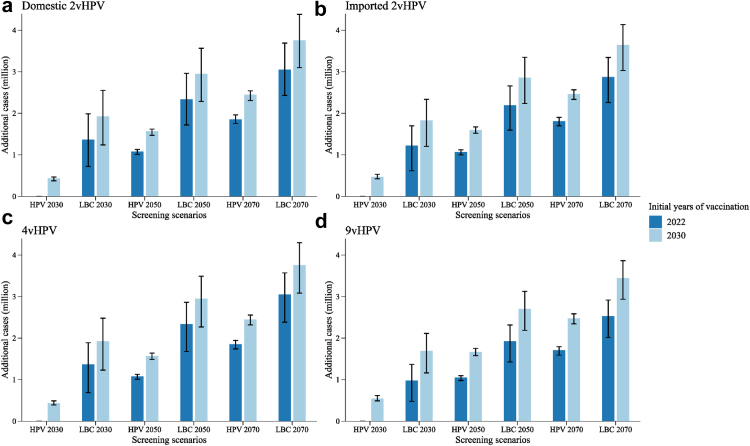
**Additional cervical cancer cases from delaying large-scale vaccination and screening program compared with no-delay scenario (vaccination initiated in 2022 and HPV 2030 screening scenario).** (a) Domestic 2vHPV; (b) imported 2vHPV; (c) 4vHPV; and (d) 9vHPV vaccines. Error bars represent the 80% uncertainty intervals of additional cases. The dark and light blue bars indicate the additional cervical cancer cases of scenarios with large-scale vaccination initiated in 2022 and 2030, respectively. “HPV 2030”, “HPV 2050”, and “HPV 2070” screening scenarios represent switching to HPV-based screening at 5-year intervals in 2022, with linearly increasing age-specific uptake from status quo in 2021, to 70% in 2030 (rapid), 2050 (moderate), and 2070 (gradual), respectively, followed by a 1% increase every year till 90% is reached. “LBC 2030”, “LBC 2050”, and “LBC 2070” screening scenarios represent maintaining LBC-based screening at 3-year intervals, with the corresponding rapid, moderate, and gradual increase in age-specific uptake. HPV, human papillomavirus; LBC, liquid-based cytology.

**Fig. 3:**
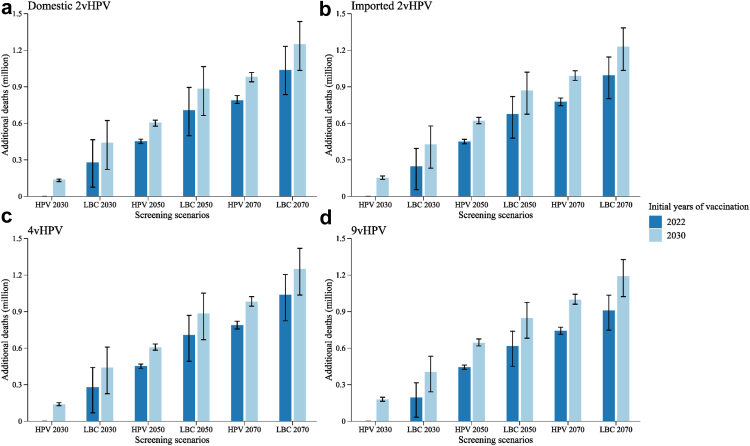
**Additional cervical cancer deaths from delaying large-scale vaccination and screening program compared with no-delay scenario (vaccination initiated in 2022 and HPV 2030 screening scenario).** (a) Domestic 2vHPV; (b) imported 2vHPV; (c) 4vHPV; and (d) 9vHPV vaccines. Error bars represent the 80% uncertainty intervals of additional deaths. The dark and light blue bars indicate the additional cervical cancer deaths of scenarios with large-scale vaccination initiated in 2022 and 2030, respectively. “HPV 2030”, “HPV 2050”, and “HPV 2070” screening scenarios represent switching to HPV-based screening at 5-year intervals in 2022, with linearly increasing age-specific uptake from status quo in 2021, to 70% in 2030 (rapid), 2050 (moderate), and 2070 (gradual), respectively, followed by a 1% increase every year till 90% is reached. “LBC 2030”, “LBC 2050”, and “LBC 2070” screening scenarios represent maintaining LBC-based screening at 3-year intervals, with the corresponding rapid, moderate, and gradual increase in age-specific uptake. HPV, human papillomavirus; LBC, liquid-based cytology.

**Fig. 4:**
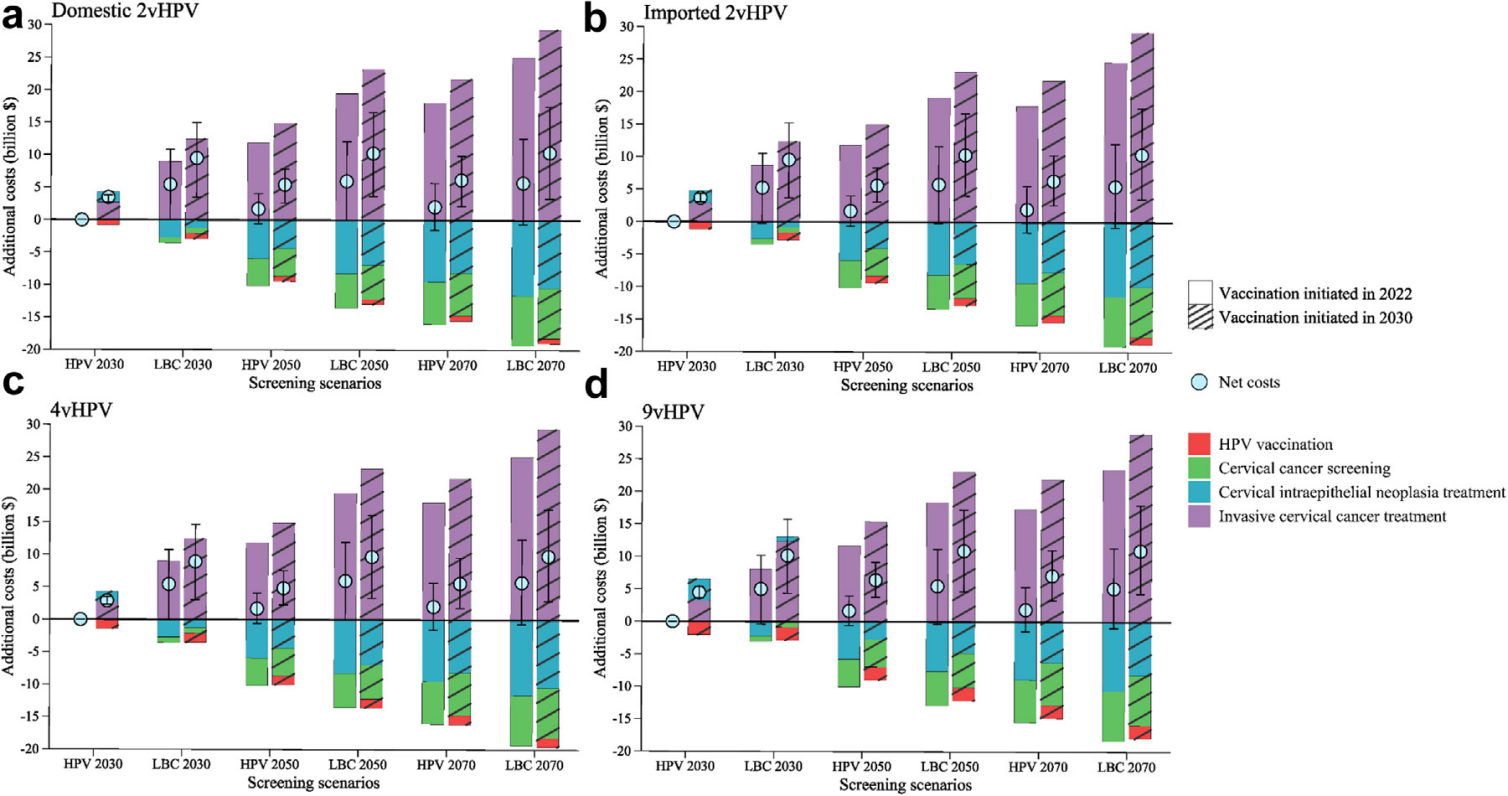
**Incremental costs from delaying large-scale vaccination and screening program compared with no-delay scenario (vaccination initiated in 2022 and HPV 2030 screening scenario), with 3% discounting.** Net costs and cost components of cervical cancer prevention and treatment are for vaccination with (a) domestic 2vHPV; (b) imported 2vHPV; (c) 4vHPV; and (d) 9vHPV vaccines. Positive values indicate increased costs and negative values indicate reduced costs. The different colored bars indicate additional costs for each component, and the blue points indicate total additional costs. Error bars represent the 80% uncertainty intervals of total additional costs. “HPV 2030”, “HPV 2050”, and “HPV 2070” screening scenarios represent switching to HPV-based screening at 5-year intervals in 2022, with linearly increasing age-specific uptake from status quo in 2021, to 70% in 2030 (rapid), 2050 (moderate), and 2070 (gradual), respectively, followed by a 1% increase every year till 90% is reached. “LBC 2030”, “LBC 2050”, and “LBC 2070” screening scenarios represent maintaining LBC-based screening at 3-year intervals, with the corresponding rapid, moderate, and gradual increase in age-specific uptake. HPV, human papillomavirus; LBC, liquid-based cytology.

Compared with the no-delay scenario, delaying vaccination by eight years but still rapidly scaling up HPV screening would result in 434,000–543,000 additional cervical cancer cases (2.94–3.44% reduction in cases averted), 138,000–178,000 deaths (2.40–2.91% reduction in deaths averted), $2,863–4,437 million net costs (13.21–16.02% reduction in costs saved), and 578,000–715,000 fewer QALYs (3.74–4.47% reduction in QALYs gained) (Fig. 2, Fig. 3, Fig. 4 and Tables S3–S9). The additional health and economic burden was spread evenly over the eight years of vaccination delays. 68.8–88.7% of these additional cases and deaths occurred in age-cohorts who missed out on vaccination due to delayed implementation, and the rest occurred in other age-cohorts who would be protected through herd effects (Fig. S4). The additional costs associated with vaccination delay were mainly due to the increased health expenditures on CIN and cervical cancer treatment ($4,272–6,486 million), which were 3.0–5.3 times the costs of vaccination over eight years (Fig. 4). Additionally, regardless of screening scenario, domestic 2vHPV vaccination initiated in 2022 would cost less and gain a few more QALYs than 9vHPV vaccination initiated in 2030 (Fig. 1, Tables S6 and S9).

**Fig. 5:**
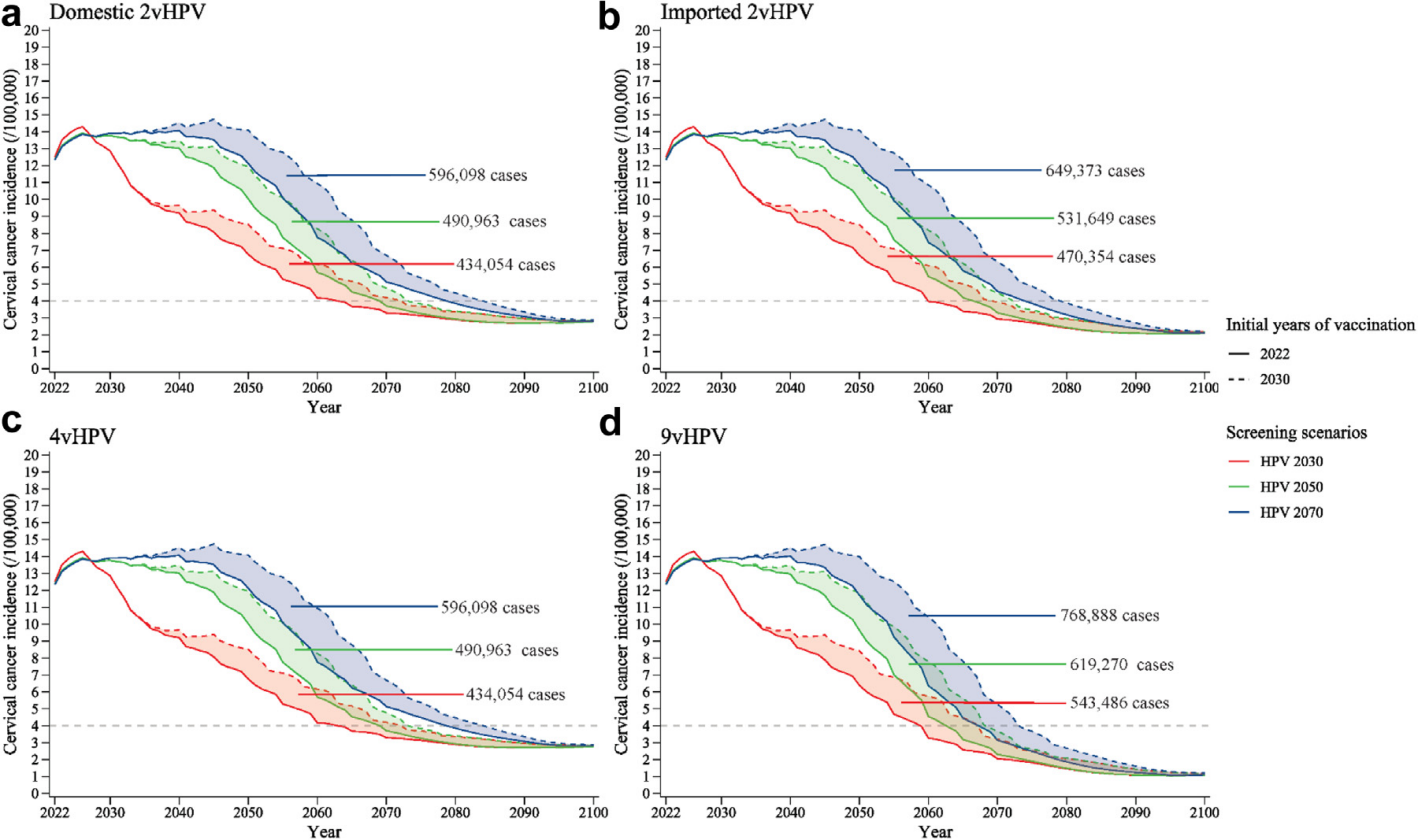
**Age-standardised cervical cancer incidence with large-scale vaccination initiated in 2022 and 2030 under HPV-based screening scenarios.** (a) Domestic 2vHPV; (b) imported 2vHPV; (c) 4vHPV; and (d) 9vHPV vaccines. The shadows represent the incidence difference between the two vaccination initiation dates and the values represent additional cases caused by the delay in HPV vaccination from 2022 to 2030 (8-year delay) under varied HPV-based screening scenarios in China. “HPV 2030”, “HPV 2050”, and “HPV 2070” screening scenarios represent switching to HPV-based screening at 5-year intervals in 2022, with linearly increasing age-specific uptake from status quo in 2021, to 70% in 2030, 2050, and 2070, respectively, followed by a 1% increase every year till 90% is reached. HPV, human papillomavirus.

**Fig. 6:**
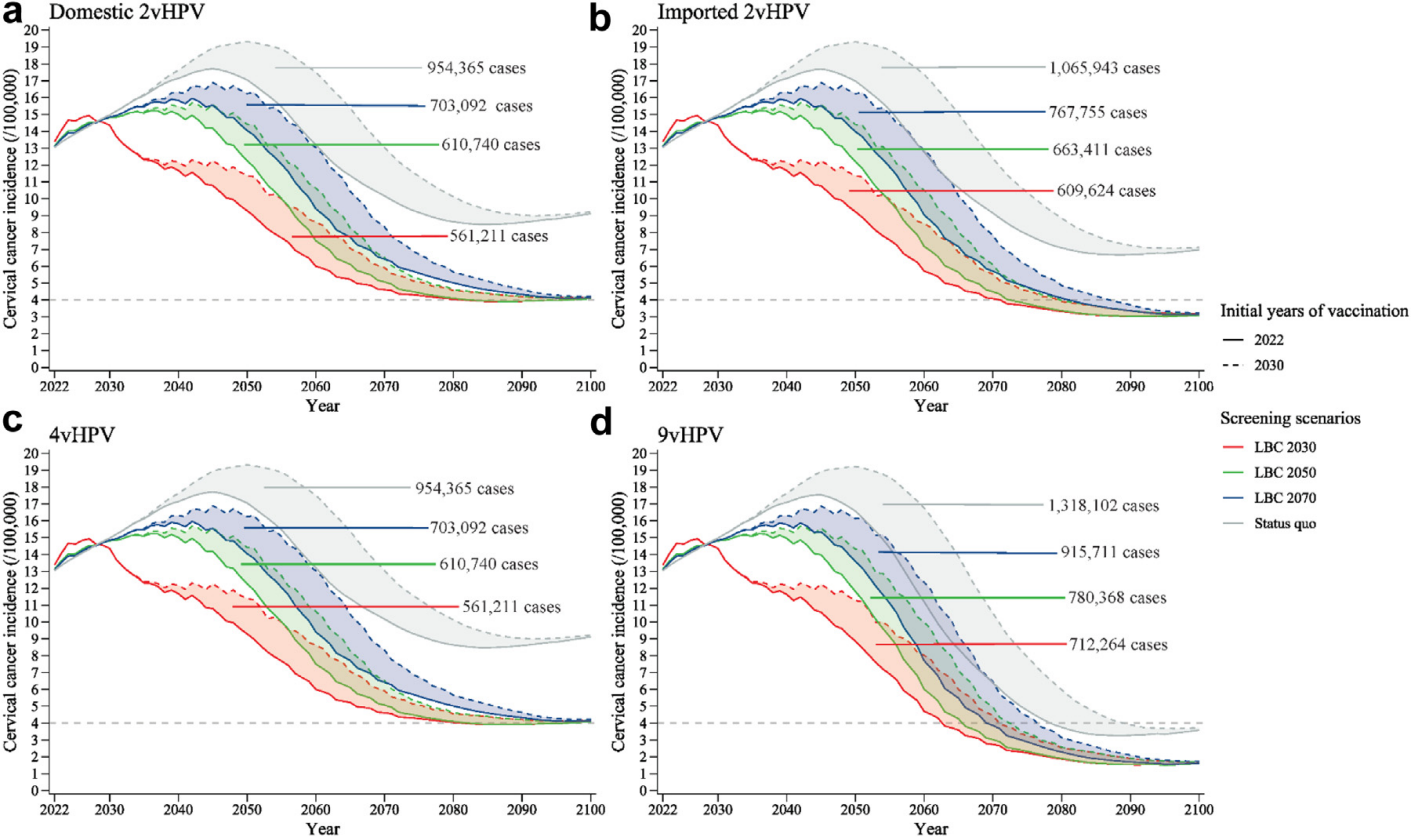
**Age-standardised cervical cancer incidence with large-scale vaccination initiated in 2022 and 2030 under LBC-based screening scenarios.** (a) Domestic 2vHPV; (b) imported 2vHPV; (c) 4vHPV; and (d) 9vHPV vaccines. The shadows represent the incidence difference between the two vaccination initiation dates and the values represent additional cases caused by the delay in HPV vaccination from 2022 to 2030 (8-year delay) under varied LBC-based screening scenarios in China. “LBC 2030”, “LBC 2050”, and “LBC 2070” screening scenarios represent maintaining LBC-based screening at 3-year intervals, with linearly increasing age-specific uptake from status quo in 2021, to 70% in 2030, 2050, and 2070, respectively, followed by a 1% increase every year till 90% is reached. Status quo represents maintaining LBC-based screening with current coverage. HPV, human papillomavirus; LBC, liquid-based cytology.

The choice of screening scenario modified the impact of vaccination delays. Under screening scenarios with lower performance or coverage, the effect of immediate large-scale vaccination was higher in terms of cases, deaths, and net costs averted (e.g., 7,570,000–10,880,000 cases averted under status quo screening vs. 3,420,000–4,430,000 cases averted under HPV 2030 screening, Tables S3–S5), and the health and economic losses associated with eight-year delayed vaccination became higher (e.g., 954,000–1,320,000 cases under status quo screening vs. 434,000–543,000 cases under HPV 2030 screening, Fig. 5, Fig. 6 and Tables S3–S5).

Not switching screening to HPV testing, or moderate or gradual scale-up of screening also increased cervical cancer cases, deaths, net costs, and QALYs lost compared with the no-delay scenario (Fig. 2, Fig. 3, Fig. 4 and Tables S3–S9). Immediate vaccination and gradual scale-up of screening without switching to HPV testing would result in 2,530,000–3,060,000 additional cervical cancer cases (16.01–20.67% reduction in cases averted), 909,000–1,040,000 deaths (14.89–18.06% reduction in deaths averted), $5,098–5,714 million net costs (18.41–26.36% reduction in costs saved), and 5,440,000–5,750,000 fewer QALYs (34.07–37.26% reduction in QALYs gained) compared with the no-delay scenario. The additional costs associated with the above screening delay were due to increased health expenditures on cervical cancer treatment exceeding reduced costs for screening and CIN treatment (Fig. 4). Also, delaying vaccination by eight years combined with gradual scale-up of LBC-based screening would result in 3,440,000–3,760,000 additional cervical cancer cases (21.81–25.43% reduction in cases averted), 1,190,000–1,250,000 deaths (19.49–21.73% reduction in deaths averted), $9,770–10,900 million net costs (39.43–45.07% reduction in costs saved), and 6,670,000–6,720,000 fewer QALYs (41.73–43.53% reduction in QALYs gained). These net costs would be negative in 2022–2032 compared to the no-delay scenario, while from 2033 to 2100, annual net costs would become positive, due to increases in the cost of invasive cervical cancer treatment (Figs. S5–S8).

In addition, under the no-delay scenario, all four types of vaccines could eliminate cervical cancer by 2059–2063, depending on the vaccine type (Fig. 5 and Table S10). Delaying vaccination by eight years but rapidly scaling up HPV screening would achieve elimination by 2068–2073 (9–10 years later compared with the no-delay scenario). However, if large-scale vaccination was never initiated, even with rapid scale-up of HPV-based screening, cervical cancer elimination would not be achieved. Moderate scale-up of HPV-based screening with immediate vaccination would achieve elimination by 2063–2070 (4–7 years later), and if scale-up was gradual, elimination would be achieved by 2068–2079 (9–16 years later). If screening was rapidly scaled up but not switched to HPV testing, immediate introduction of imported 2vHPV and 9vHPV vaccination could eliminate cervical cancer by 2072 (12 years later) and 2063 (4 years later), respectively, whereas domestic 2vHPV and 4vHPV could not lead to elimination, with age-standardised incidence of 4.09–4.21 cases per 100,000 woman in 2100. Moreover, if cervical cancer screening in China was unchanged from the status quo, only 9vHPV routine vaccination could eliminate cervical cancer, with the elimination year of 2079 if large-scale vaccination was initiated in 2022 and 2090 if the vaccination was initiated in 2030.

### Sensitivity analysis

We assessed the effect of varying vaccination costs on the results of the cost-effectiveness analysis. When assuming current market vaccine prices, 4vHPV and 9vHPV vaccination combined with screening was not cost-effective using GDP per capita as the threshold, thus the optimal scenario was rapid scale-up of HPV-based screening alone with 70% coverage reached by 2030. The no-delay scenario was otherwise always the optimal intervention scenario, and often saved costs and dominated all alternatives, regardless of vaccine prices (Figs. S9–S12). Deterministic sensitivity analysis showed that the model was sensitive to treatment costs, discount rate, screening sensitivity, screening costs, and maximum achievable screening coverage (Figs. S13–S16). However, the no-delay scenario always remained cost-saving compared with status quo. In the probabilistic sensitivity analysis, the uncertainty ranges of parameters had a small effect on the number of additional cases, deaths, and costs associated with delayed large-scale vaccination programs, as well as the elimination year. However, these parameters had a little higher effect on the additional number associated with delays in large-scale screening programs (Fig. 2, Fig. 3, Fig. 4 and Tables S10). This effect may be mainly due to the uncertainty in screening sensitivity and treatment costs, based on the results of deterministic sensitivity analysis. The no-delay scenario remained more effective than status quo, though several model runs entailing sample parameter values for the scenario showed a slight increase in costs compared with status quo (Fig. S17).

## Discussion

Our study demonstrated that if a large-scale HPV vaccination and screening program was initiated in 2022 with a switch of screening method to HPV testing and a rapid increase in screening coverage to 70% by 2030, this would cost less and gain more QALYs compared to delaying the programs by several years. This suggests that HPV vaccination and screening programs should be implemented as early as possible. The immediate program can avert 14.8–15.8 million cases and 5.75–6.11 million deaths and save $21.7–27.7 billion costs in China compared to the status quo intervention, and achieve cervical cancer elimination by 2060s. We found that short delays in the initiation of large-scale vaccination, no switch of screening to HPV testing, or slower increases in screening coverage would increase disease burden and costs, as well as delay the timeline for cervical cancer elimination in China. The negative health and economic impacts of delayed vaccination were larger under scenarios where screening uptake and modalities were suboptimal. Also, if the vaccination was never initiated, China would not achieve cervical cancer elimination by the end of the century even if the screening target was reached in 2030. If the current screening uptake was maintained or screening was not switched to HPV testing, neither domestic 2vHPV nor 4vHPV vaccination would allow China to achieve the elimination.

Most modelling studies show HPV vaccination to be a highly cost-effective intervention in China.^26^^,^^27^ Immediate vaccine implementation is likely to bring greater economic returns than delaying the decision. However, few studies have explicitly quantified the cost of delay. Our study demonstrated that compared with the no-delay scenario, the delayed scenario with an eight-year delay in vaccination and a gradual scale-up of LBC-based screening could lead to about 40% decrease in costs saved and QALYs gained. While large-scale vaccination and screening programs will eventually reduce costs, there will be a delay before these economic gains are realised due to the lag between primary prevention and cancer. This implies that to swiftly implement large-scale vaccination and screening programs, China needs to increase investment in cervical cancer prevention in the first few years. Vaccine price was a key determinant for the cost-effectiveness of HPV vaccination. For the base case analysis, we assumed lower NIP vaccine prices because the Chinese government will be able to negotiate prices when initiating a large-scale program and purchasing large volumes per year. The negotiated price was benchmarked to PAHO Revolving Fund prices given that China is at a similar stage of economic development to many large PAHO countries,^28^ and has a potential market size that exceeds that of all PAHO countries combined. Domestic and imported 2vHPV vaccines, which were already cost-effective at the private market price in the sensitivity analysis and in previous studies,^26^ would be cost-saving at the potentially lower negotiated price. In contrast, 4vHPV and 9vHPV vaccines were not cost-effective at the private market price but changed to cost-saving at the negotiated price, which suggested the importance of vaccine price reduction.

Our study quantified both the direct and indirect health effects of delayed vaccination in China. High-coverage HPV vaccination programs have significantly reduced vaccine-type HPV prevalence for both vaccinated and unvaccinated women and the incidence of related precancerous lesions in several countries.^29^^,^^30^ Although previous studies^13^^,^^15^ have shown that delayed vaccination policies would lead to a considerable health burden in China, their estimates were relatively conservative as they did not consider indirect effects or future changes in sexual behavior. Our study addressed these limitations, showing that each year of delay in the initiation of a large-scale vaccination program could result in an average of more than 119 thousand cervical cancer cases and 42 thousand deaths over the lifetime of women under status quo screening. Delayed vaccination would also substantially delay cervical cancer elimination, despite having little effect on the yearly incidence by the end of the century. Because of the trends of population aging and increased sexual activity in China, cervical cancer incidence in China is projected to continue to rise under current cervical cancer prevention strategies.^22^ Consequently, we found that if screening was not changed, each year of delay to vaccine initiation could result in longer than one year of delay to achieving elimination.

Even if a large-scale high-coverage HPV vaccination program is immediately implemented, large-scale HPV-based cervical screening is still needed for cervical cancer control in China. The current proportion of screened women who receive an HPV test is still low. For instance, more than 90% of women in the National Cervical Cancer Screening Program in Rural Areas (NCCSPRA) still used low-performance cytology or visual inspection with acetic acid (VIA) in 2018.^31^ Consistent with a previous randomised controlled trial in primary healthcare settings in China,^32^ our modelling study showed that a large-scale screening program with a switch to HPV testing was most effective and least costly. Notably, if screening was not switched to HPV testing, domestic 2vHPV and 4vHPV vaccination could not lead to elimination. Considering that domestic vaccines are most likely to be introduced into China's NIP due to their low prices and more secure supplies, the findings indicate the necessity of HPV-based screening as well as a future switch to a domestic 9vHPV vaccine still under development. In addition, population screening coverage is only 21.4% in China,^12^ and slowing down the increase in screening coverage will also reduce health and economic benefits. Our study made the assumption that although the screening coverage increases gradually in the delayed program scenarios, it will eventually reach the WHO target of 70% and then continue to increase further to 90%. If the screening target is not reached, low screening coverage could result in greater losses than what we have estimated here.

Our study indicated that health and economic losses associated with delayed vaccination were greatest when screening performance was unfavourable. This is because women who miss out on vaccination due to delays in vaccination initiation may also miss out on secondary cancer prevention due to suboptimal screening. Consistent with our findings, global analyses have suggested that vaccination is the most cost-effective in settings where screening programs are limited.^33^^,^^34^ These findings emphasise the urgency of initiating a large-scale HPV program while cervical screening is gradually improved, since the latter is likely to take more time. Given China's large population and significant variations in healthcare resources between urban and rural regions, it will be a challenge to achieve national screening goals in a short period of time.^35^ Earlier initiation of large-scale HPV vaccination could help to relieve pressure on secondary and tertiary prevention. Furthermore, earlier initiation of vaccination may also benefit health equity, since it would narrow geographical disparities in cervical cancer burden resulting from varied screening participation and quality, as well as differential uptake of vaccination in the private sector. A previous study^36^ also demonstrated that HPV vaccination programs would provide higher financial protection to women in poor households and contribute to more equitable health.

The main challenges to national vaccination and screening programs in China are limited resources, including vaccine supply shortages and budget constraints.^8^ However, these are not insurmountable barriers. We assumed immediate scaling up of single-age cohort vaccination to 90% coverage, which is achievable through adequate resource allocation by the government, because the total number of doses of the four vaccines purchased privately in 2020 (15 million)^37^ exceeded the doses needed to vaccinate 12-year-old girls (approximately 7 million girls, 90% coverage, 2 doses per girl). Indeed, these requirements could be further lowered given the latest WHO recommendation that a single HPV dose may be sufficient for 12-year-old girls.^38^ So far, five HPV vaccines have been approved in China for preventing cervical cancer, including the four vaccines examined in this study and an additional domestically produced 2vHPV vaccine (Walrinvax™) approved in 2022. With future increases in production capacity for domestic vaccines and the development of a domestic low-cost 9vHPV vaccine, vaccine shortages will likely ease, and vaccine prices may decrease further, especially if vaccines can be purchased as a part of a national tender.

There are several strengths to our study. To our knowledge, this is the first analysis incorporating a transmission dynamic model to comprehensively assess the health and economic impacts of delaying large-scale HPV vaccination and screening programs in China. Our study not only assessed the impact of implementing large-scale vaccination and screening programs but also explored the impact of the programs' initiation time and scale-up pace. Our results provide further evidence to support the government's commitments to timely implementation of vaccination and screening programs and to address financial and attitudinal barriers to uptake. Additionally, we considered different outcomes relevant to policy-making, including effectiveness, cost-effectiveness, and time to cervical cancer elimination, providing policymakers the comprehensive evidence. Furthermore, we incorporated demographic trends in aging, urbanisation, and sexual activity, which may increase cervical cancer incidence in the future.^7^^,^^22^

Our study also has several limitations. First, the impact of delay in HPV vaccination and screening program in this study may be underestimated since other diseases related to HPV beyond cervical cancer were not considered. In particular, we may have underestimated the cost-effectiveness of 4vHPV and 9vHPV since we did not consider the benefit of preventing anogenital warts in both females and males. Second, we assumed homogeneous vaccine coverage throughout China. Due to China's wide geographic span and heterogeneous economic development levels, it may not be possible to achieve 90% vaccination coverage in all regions rapidly and simultaneously. If the coverage target is achieved slowly in less socioeconomically developed areas, delay in vaccination may result in greater losses than we estimated. Third, we did not include the scenarios of male vaccination or catch-up vaccination, because according to WHO's global strategy for cervical cancer elimination,^3^ the primary target is vaccinating young girls who could directly benefit from it. WHO's Strategic Advisory Group of Experts on Immunisation (SAGE) has recommended that vaccinating boys or older women (>15 years) should be delayed until current vaccine supply constraints are alleviated.^39^ If the vaccine shortage is alleviated in the future, vaccination of boys and older women may accelerate the decline in incidence. Fourth, our model did not account for the effect of HIV or other sexually transmitted infections (STIs), which might increase the risk of HPV acquisition and cervical cancer progression. Hence, the effect of HPV vaccination and screening on cervical cancer burden in women living with STIs might be overestimated. Furthermore, we did not consider other interventions which may affect acquisition of HPV, including sex safety education, barrier contraception such as condoms, antimicrobial prophylaxis for STIs, and the potential vaccine development for other STIs. However, this may have little impact on our findings because our study focused on the general population, where the prevalence of HIV and other related STIs prevalence is low.^40^

### Conclusion

In conclusion, our study shows that large-scale HPV vaccination and HPV-based screening programs are both necessary to eliminate cervical cancer in China. Delaying national scale-up of HPV vaccination and/or high-performance screening in China has detrimental consequences in terms of morbidity, mortality, and economic costs related to cervical cancer, as well as the timeline to elimination. These findings should spur health authorities to expedite national vaccine rollout and screening improvement to maximise the health and economic benefits. Large-scale vaccination programs are particularly important in regions where screening is still suboptimal and can only be gradually improved.

## Contributors

FZ, CW, MG, and SH conceived and designed the study. FZ and MJ contributed to funding acquisition of the study. MG and TY accessed and verified all reported data. MG, SH, XZ, and TY contributed to the analysis and visualization of the study. MG drafted the manuscript. MJ, YL, and YQ critically revised the manuscript for intellectual content. All authors approved the final version of the manuscript. All authors had full access to all the data in the study and had final responsibility for the decision to submit for publication.

## Data sharing statement

This study does not involve any patient data or participant data. Readers can access the data used in this study from the links to public domain resources provided in the Methods. The code used to generate the reported estimates is sensitive, interested parties should contact the corresponding author for more information.

## Declaration of interests

YQ and FZ report grants from GlaxoSmithKline Biologicals, Merck & Co, and Xiamen Innovax Biotech to their institution, to undertake clinical trials on the human papillomavirus (HPV) vaccine. MJ has previously received research grants (unrelated to this paper) from the Bill & Melinda Gates Foundation, the National Institute for Health Research, Research Councils UK, Gavi, the Vaccine Alliance, the European Commission, and Wellcome Trust. Other co-authors declare no competing interests.
