# Supplementary material for: Health and economic impact of delaying large-scale HPV vaccination and screening implementation on cervical cancer in China: a modelling study
Source: Lancet Reg Health West Pac. 2023 Apr 20;36:100768. doi: 10.1016/j.lanwpc.2023.100768 (PMC10398607; doi:10.1016/j.lanwpc.2023.100768)
Supplement: Supplementary material [file mmc1.docx]

**Health and economic impact of delaying large-scale HPV vaccination and screening implementation on cervical cancer in China: a modelling study**

**Supplementary appendix**

Contents

[1. Methods 2](#_Toc120832404)

[1.1 Model structure 2](#_Toc120832405)

[1.2 Model assumptions and inputs 3](#_Toc120832406)

[1.2.1 Vaccine prices 3](#_Toc120832407)

[1.2.2 Treatment costs 3](#_Toc120832408)

[1.3 Demographic and epidemiological data 6](#_Toc120832409)

[1.4 Alternative scenarios 6](#_Toc120832410)

[1.4.1 Screening scenarios 6](#_Toc120832411)

[2. Results 12](#_Toc120832412)

[2.1 Main results 12](#_Toc120832413)

[2.2 Sensitivity analysis 43](#_Toc120832414)

# **1. Methods**

## **1.1 Model structure**

The transmission dynamic model in this study consists of a dynamic model and a natural history model, and the model structure is shown in Figure S1. The dynamic model was used to simulate the human papillomavirus (HPV) transmission between males and females, whereas the natural history model was employed to simulate the natural history of cervical cancer and to obtain the number of cervical cancer cases and deaths associated with HPV infections.

In the dynamic model, the sexual mixing matrices for each stratum were calculated by use of partner acquisition rates and assortativity of age, area, and sexual activity.^1^ Partner acquisition rates were adjusted to maintain the number of male-female sexual partnerships.^1^ We incorporated 13 HPV strains (types 16, 18, 31, 33, 35, 39, 45, 51, 52, 56, 58, 59, and 68), which were high-risk for the development of cervical cancer and its precursor lesions. The force of infection (FOI) and prevalence for every high-risk HPV type were simulated separately, because of the potential variations in transmission probability between every sexual pair and the substantial differences in vaccine efficacy for each high-risk HPV type. Natural immunity would be acquired for a period of time after an HPV type was cleared, however, this infection-acquired immunity would wane over time and only protected against reinfection with the same HPV type, without cross-protection against other HPV types.

In the natural history model, neonates are assumed to be in the uninfected state at birth, and all-cause mortality was present in all states. Each individual was entered into the model at their birth year if they were born after 2015 or at their age in 2015, and they were then randomly allocated to a new state within the model based on the transition probabilities. Individuals were transitioned between states representing no current or previous infection or vaccine (in which they were considered susceptible to infections); immunity (including infection-acquired immunity and vaccine-acquired immunity); infection; development of cervical intraepithelial neoplasia (CIN) grade 1, CIN2, or CIN3 disease (representing mild, moderate, and severe dysplasia); effective detection and treatment; and cervical cancer likely to cause death or to extend until the simulation end point.^1^ Local cervical cancer without symptoms may become symptomatic or progress to more advanced stages of cervical cancer without symptoms. In the absence of screening, cervical cancer is diagnosed only when symptoms develop in which the patient is immediately treated. Females with symptomatic cervical cancer are subjected to the age-specific death and cure probability of cervical cancer. Cancer patients who remain alive 5 years after cancer diagnosis are moved to the health state “cured” and are removed from the model in the rest of simulations.^2^

Vaccination and screening interventions were embedded into the model at different simulation stages. If HPV vaccination is available, girls aged 12 years were considered to be vaccinated at predefined coverage in the dynamic model, and vaccinated women acquired lifelong immunity based on vaccine efficacy. If cervical cancer screening is available, women at the target age for screening and without a diagnosis of cervical cancer were considered to be screened at predefined coverage in the natural history model. HPV infection, CIN, and cervical cancer will be detected based on the sensitivity of the screening scenario.


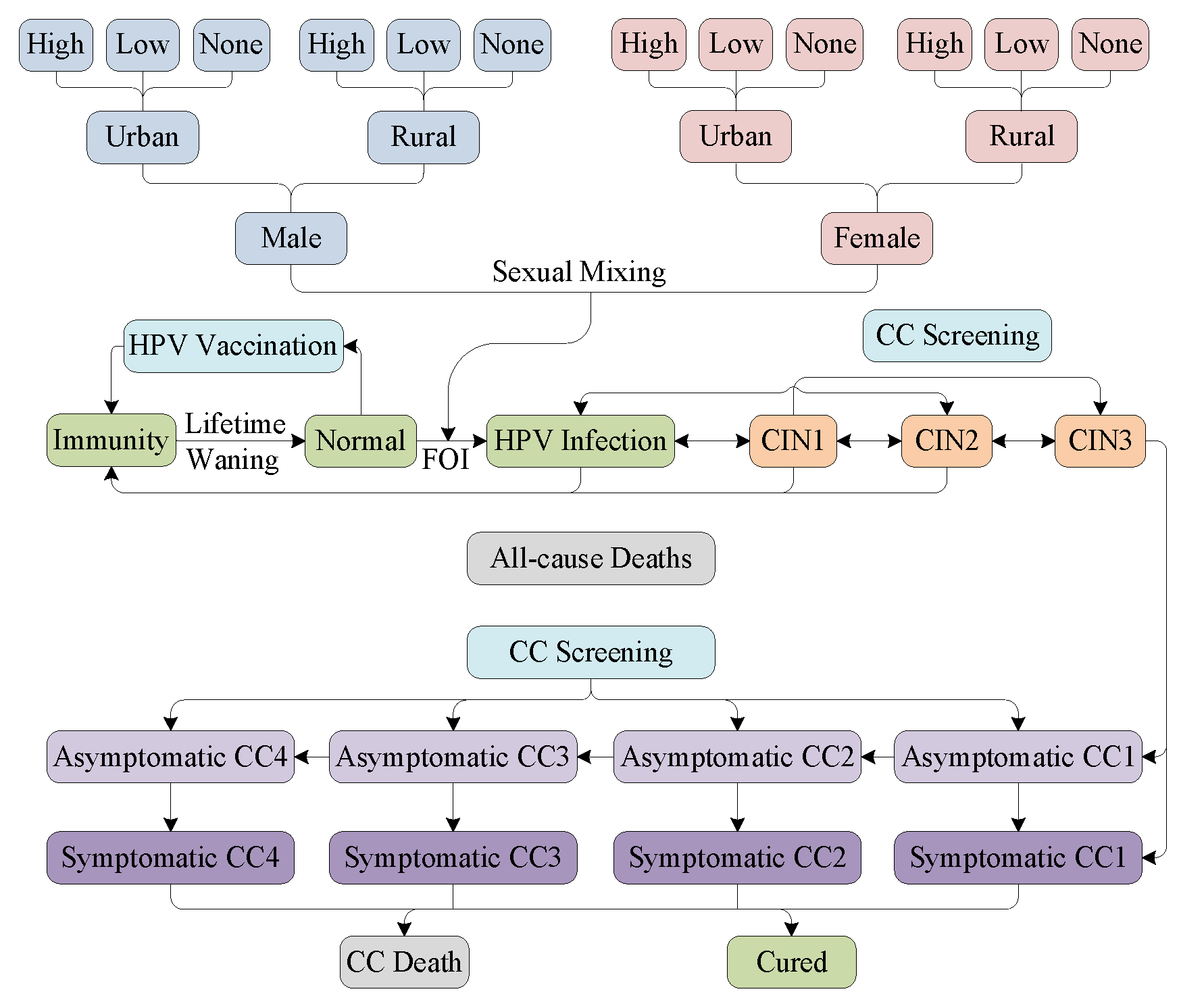


**Figure S1. Model structure**

Individuals with multiple sexual partners are regarded as having high sexual activity level, while individuals with only one sexual partner are regarded as having low sexual activity level.

Abbreviations: HPV, human papillomavirus; FOI, force of infection; CIN, cervical intraepithelial neoplasia; CC, cervical cancer.

## **1.2 Model assumptions and inputs**

## **1.2.1 Vaccine prices**

The current private market prices of HPV vaccines in China are $50·62, $89·23, $122·77, and $199·69 per dose for domestic 2vHPV, imported 2vHPV, 4vHPV, and 9vHPV vaccines, respectively. In the large-scale vaccination program, China could likely negotiate lower public sector prices as part of a government tender. We assumed the base case price for 4vHPV vaccine based on the price paid by the Pan American Health Organization (PAHO) Revolving Fund in 2022,^3^ given that China is at a similar stage of economic development to many large PAHO countries^4^ and has bargaining power from a large potential market size. Due to the limited price information of the other three vaccine types, we calculate them using the 4vHPV vaccine base case price ($10·48 per dose) as reference and applying the ratio of prices for each vaccine in the current private market in China. Therefore, domestic 2vHPV, imported 2vHPV, and 9vHPV vaccines were estimated to cost $4·32, $7·62, and $17·05 per dose, respectively.

## **1.2.2 Treatment costs**

The treatment cost for CIN and cervical cancer cases were collected in our nationwide multicenter cross-sectional, hospital-based survey. The study was conducted from August 2020 to June 2021, including 26 hospitals in seven administrative regions of China. All components of direct medical costs, direct non-medical costs, and indirect costs (productivity loss) for women diagnosed with CIN and invasive cervical cancer were included.

Model parameters were obtained from published literature and government-released online datasets, which were summarised in Table S1. The range of values tested in the sensitivity analysis for each parameter was either based on the reported 95% confidence intervals in the referenced studies or determined by assuming a 25% change from the base-case value.

**Table S1. Model inputs**

| **Parameter** | **Base case** | **Range** | **Distribution** | **Source** |  |
| --- | --- | --- | --- | --- | --- |
| **Probability of HPV clearance** | |  |  |  | |
| 12-24 years | 0.7188 | 0.6463-0.7830 | β | ^5-8^ |  |
| 25-29 years | 0.6984 | 0.5898-0.7952 | β | ^5-8^ |  |
| 30-39 years | 0.3503 | 0.2860-0.4188 | β | ^5-8^ |  |
| 40-49 years | 0.2048 | 0.1118-0.3022 | β | ^5-9^ |  |
| >50 years | 0.1004 | 0.0546-0.1567 | β | ^5-8^ |  |
| **Probability of progression and regression** | |  |  |  | |
| Infection to CIN1 | 0.075 | 0.056-0.1154 | β | ^5,10^ |  |
| CIN1 to CIN2 | 0.2240 | 0.1608-0.2972 | β | ^5-8^ |  |
| CIN2 to CIN3 | 0.3498 | 0.0904-0.8654 | β | ^5-8^ |  |
| CIN3 to CC Ⅰ | 0.1019 | 0.0189-0.2906 | β | ^5-8^ |  |
| CIN2 to CIN1 | 0.2494 | 0.1994-0.2992 | β | ^5-8^ |  |
| CIN3 to CIN2 | 0.0135 | ±25% | β | ^5^ |  |
| CIN2 to infection/immunity | 0.1901 | ±25% | β | ^5,11^ |  |
| CIN1 to CIN3 | 0.0464 | 0.0098-0.1297 | β | ^5-8^ |  |
| CC Ⅰ to CC Ⅱ | 0.4376587 | ±25% | β | ^10,12^ |  |
| CC Ⅱ to CC Ⅲ | 0.5358411 | ±25% | β | ^10,12^ |  |
| CC Ⅲ to CC Ⅳ | 0.6837722 | ±25% | β | ^10,12^ |  |
| Waning of natural immunity | 0.021 | 0.015-0.027 | β | ^13^ |  |
| **Cancer symptoms** |  |  |  |  |  |
| CC Ⅰ | 0.15 | ±25% | β | ^10,12^ |  |
| CC Ⅱ | 0.225 | ±25% | β | ^10,12^ |  |
| CC Ⅲ | 0.6 | ±25% | β | ^10,12^ |  |
| CC Ⅳ | 0.9 | ±25% | β | ^10,12^ |  |
| **Vaccine efficacy** |  |  |  |  |  |
| Target types | 1 | 0.8-1.0 | Binomial | Assumed^14-16^ |  |
| **Cross protection** |  |  |  |  |  |
| Domestic 2vHPV vaccine against type 31 | 0.462 | 0.153-0.664 | β | ^17^ |  |
| Imported 2vHPV vaccine against type 31 | 0.771 | 0.672-0.844 | β | ^17^ |  |
| Imported 2vHPV vaccine against type 33 | 0.431 | 0.193-0.602 | β | ^17^ |  |
| Imported 2vHPV vaccine against type 45 | 0.790 | 0.613-0.894 | β | ^17^ |  |
| 4vHPV vaccine against type 31 | 0.462 | 0.153-0.664 | β | ^17^ |  |
| **Screening sensitivity** |  |  |  |  |  |
| **Liquid-based cytology** |  |  |  |  |  |
| CIN1 | 0.47 | ±25% | β | ^18^ |  |
| CIN2/3 | 0.53 | 0.486-0.574 | β | ^18^ |  |
| CC Ⅰ | 0.90 | ±25% | β | Assumed |  |
| CC Ⅱ | 0.92 | ±25% | β | Assumed |  |
| CC Ⅲ | 0.95 | ±25% | β | Assumed |  |
| CC Ⅳ | 0.99 | ±25% | β | Assumed |  |
| **HPV test** |  |  |  |  |  |
| CIN1 | 0.80 | ±25% | β | Assumed |  |
| CIN2 | 0.90 | ±25% | β | Assumed^16^ |  |
| CIN3 | 0.94 | ±25% | β | Assumed^16^ |  |
| CC Ⅰ-Ⅳ | 1.00 | .. | .. | Assumed |  |
| **Precancerous lesions management** | |  |  |  | |
| **Urban** |  |  |  |  |  |
| Follow-up of CIN1 | 0.4631 | 0.4168-0.5094 | β | ^1^ |  |
| Treatment of CIN1 | 0.4421 | 0.3979-0.4863 | β | ^1^ |  |
| Treatment of CIN2/3 | 0.9533 | 0.8580-1.0000 | β | ^1^ |  |
| **Rural** |  |  |  |  |  |
| Follow-up of CIN1 | 0.3871 | 0.3484-0.4258 | β | ^1^ |  |
| Treatment of CIN1 | 0.4535 | 0.4082-0.4989 | β | ^1^ |  |
| Treatment of CIN2/3 | 0.8946 | 0.8051-0.9841 | β | ^1^ |  |
| **Treatment efficacy** |  |  |  |  |  |
| **Urban** |  |  |  |  |  |
| CIN1 treatment efficacy | 1.0000 | 0.9956-1.0000 | .. | ^1^ |  |
| CIN2/3 treatment efficacy | 0.9367 | 0.9278-0.9455 | β | ^1^ |  |
| **Rural** |  |  |  |  |  |
| CIN1 treatment efficacy | 0.9978 | 0.9943-1.0000 | β | ^1^ |  |
| CIN2/3 treatment efficacy | 0.9000 | 0.8846-0.9154 | β | ^1^ |  |
| **Costs (2021 US$)** |  |  |  |  |  |
| **Vaccine price per dose** |  |  |  |  |  |
| Domestic 2vHPV vaccine | 4.32 | ±25% | γ | Assumed |  |
| Imported 2vHPV vaccine | 7.62 | ±25% | γ | Assumed |  |
| 4vHPV vaccine | 10.48 | ±25% | γ | Assumed^3^ |  |
| 9vHPV vaccine | 17.05 | ±25% | γ | Assumed |  |
| **Vaccination service cost** | 4.12 | ±25% | γ | ^19^ |  |
| **Screening and treatment costs** |  |  |  |  |  |
| **Urban** |  |  |  |  |  |
| LBC-based screening | 10.38 | ±25% | γ | ^1^ |  |
| HPV-based screening | 16.29 | ±25% | γ | ^2^ |  |
| CIN1 treatment | 1797.6 | 1008.99-2913.43 | γ | See context |  |
| CIN2/3 treatment | 2648.47 | 1668.01-4099.76 | γ | See context |  |
| CC Ⅰ treatment | 18789.69 | 13662.98-26314.34 | γ | See context |  |
| CC Ⅱ treatment | 27040.1 | 18538.16-41649.1 | γ | See context |  |
| CC Ⅲ treatment | 29579.14 | 19090.04-48599.27 | γ | See context |  |
| CC Ⅳ treatment | 29579.14 | 19090.04-48599.27 | γ | See context |  |
| **Rural** |  |  |  |  |  |
| LBC-based screening | 7.54 | ±25% | γ | ^1^ |  |
| HPV-based screening | 13.98 | ±25% | γ | ^2^ |  |
| CIN1 treatment | 444.06 | 157.91-1436.56 | γ | See context |  |
| CIN2/3 treatment | 1249.82 | 514.48-2107.96 | γ | See context |  |
| CC Ⅰ treatment | 18789.69 | 13662.98-26314.34 | γ | See context |  |
| CC Ⅱ treatment | 27040.1 | 18538.16-41649.1 | γ | See context |  |
| CC Ⅲ treatment | 29579.14 | 19090.04-48599.27 | γ | See context |  |
| CC Ⅳ treatment | 29579.14 | 19090.04-48599.27 | γ | See context |  |
| **Utilities (quality of life)** | |  |  |  | |
| 6 month posttreatment of CIN1 | 0.97 | ±25% | β | ^20^ |  |
| pretreatment of CIN2/3 | 0.90 | 0.8734-0.9266 | β | ^21^ |  |
| 1 month posttreatment of CIN2/3 | 0.96 | 0.9445-0.9755 | β | ^21^ |  |
| 3 month posttreatment of CIN2/3 | 0.98 | 0.9445-0.9944 | β | ^21^ |  |
| 6 month posttreatment of CIN2/3 | 0.99 | 0.9856-0.9944 | β | ^21^ |  |
| pretreatment of CC Ⅰ-Ⅱa | 0.83 | 0.7875-0.8725 | β | ^21^ |  |
| 1 month posttreatment of CC Ⅰ-Ⅱa | 0.77 | 0.7232-0.8168 | β | ^21^ |  |
| 3 month posttreatment of CC Ⅰ-Ⅱa | 0.84 | 0.7975-0.8825 | β | ^21^ |  |
| 6 month posttreatment of CC Ⅰ-Ⅱa | 0.94 | 0.9145-0.9655 | β | ^21^ |  |
| pretreatment of CCⅡb-Ⅳ | 0.84 | 0.7731-0.9069 | β | ^21^ |  |
| 1 month posttreatment of CC Ⅱb-Ⅳ | 0.68 | 0.5674-0.7926 | β | ^21^ |  |
| 3 month posttreatment of CC Ⅱb-Ⅳ | 0.75 | 0.6409-0.8591 | β | ^21^ |  |
| 6 month posttreatment of CC Ⅱb-Ⅳ | 0.86 | 0.8213-0.8987 | β | ^21^ |  |
| terminal care | 0.288 | ±25% | β | ^20^ |  |
| **Achievable coverage of vaccination** | 0.90 | 0.70-0.95 | Binomial | Assumed^22^ |  |
| **Achievable coverage of screening** | 0.90 | 0.70-0.90 | Binomial | ^22,23^ |  |
| **Discount rate** | 0.03 | 0-0.05 | Binomial | ^24^ |  |

Abbreviations: CIN, cervical intraepithelial neoplasia; CC, cervical cancer; LBC, liquid-based cytology; HPV, Human Papillomavirus.

Urban population refers to all people residing in urban areas, while rural population refers to people residing in rural areas.

## **1.3 Demographic and epidemiological data**

Demographic and epidemiological data were obtained from open-source publications or government-released online datasets^25-32^ and were reported in the previous study.^2^ Population size in 2015 was obtained from National Bureau of Statistics of China.^25,26^ The all-cause mortality rate was obtained from National Health Commission of China.^27^ Age-specific incidence and mortality of cervical cancer in 2015 were obtained from Chinese Cancer Registry Report released by National Cancer Center of China.^28^ In addition, the data sources and analytical methods of HPV prevalence, fertility rate, sexual activity, and screening coverage were reported in the previous study.^1^ The projected fertility rate of women aged 15-49 years^29^ and urbanisation data^30,31^ were from the UN Population Division. The patterns in sexual activity in China were projected with historical data from the USA as a reference and were adjusted for the Human Development Index.^32^

## **1.4 Alternative scenarios**

The intervention scenarios include a combination of 10 vaccination initiation scenarios and seven screening scenarios, which are summarised in Table S2. The averted numbers of cases, deaths, costs, and quality-adjusted life-years (QALYs) were estimated compared with the status quo scenario where large-scale vaccination was not initiated and maintaining the current screening. The additional numbers of cases, deaths, costs, and QALYs associated with vaccination and screening delay were estimated compared with the no-delay scenario where large-scale vaccination was initiated in 2022 and HPV-based screening was rapidly scaled up to 70% coverage in 2030, followed by a 1% increase every year till 90% is reached.

## **1.4.1 Screening scenarios**

The screening scenarios were assumed to involve liquid-based cytology (LBC)-based and HPV-based screening. For LBC-based screening scenarios, women with atypical squamous cell of undetermined significance (ASC-US+) will be referred to colposcopy. Women with negative cytology will recall for routine screening at 3-year intervals. The sensitivity of LBC-based screening scenario was 0.47 to detect CIN1, and 0.53 to detect CIN2/3 (Table S1). HPV-based screening scenario used HPV DNA testing as primary screening (with genotyping) and cytology triage, namely women positive for HPV16/18 will be referred to colposcopy and women positive for other 11 oncogenic types will be triaged with LBC. Women negative for HPV test will recall for routine screening at 5-year intervals. The sensitivity of HPV-based screening scenario was 0.80, 0.90, and 0.94 to detect CIN1, CIN2, and CIN3, respectively (Table S1).

The proportion of screening women in compliance with routine cervical screening is estimated from the national wide expert panel review. The results suggested that about 95.33% of urban women and 89.46% of rural women will receive treatment after the diagnosis of CIN2/3. As to CIN1, about 44.21% of urban women, and 45.35% of rural women will receive treatment (Table S1). Among the women who receive treatment, about 100% and 93.70% of urban women, and 99.80% and 90.00% of rural women were successfully treated after the treatment of CIN1 and CIN2/3, respectively (Table S1).

The current age-specific screening coverage was derived from a nationwide survey (Figure S2).^33,34^ The overall screening coverage over time for target population of status quo screening and improved screening scenarios is shown in Figure S3. These seven screening scenarios are as follows: (1) HPV 2030: switching to 5-yearly HPV-based screening in 2022, with a rapid linear increase in age-specific uptake from status quo in 2021, to 70% in 2030, followed by a 1% increase every year till 90% is reached; (2) LBC 2030: maintaining 3-yearly LBC-based screening, with a rapid linear increase in age-specific uptake from status quo in 2021, to 70% in 2030, followed by a 1% increase every year till 90% is reached; (3) HPV 2050: switching to 5-yearly HPV-based screening in 2022, with a rapid linear increase in age-specific uptake from status quo in 2021, to 70% in 2050, followed by a 1% increase every year till 90% is reached; (4) LBC 2050: maintaining 3-yearly LBC-based screening, with a rapid linear increase in age-specific uptake from status quo in 2021, to 70% in 2050, followed by a 1% increase every year till 90% is reached; (5) HPV 2070: switching to 5-yearly HPV-based screening in 2022, with a rapid linear increase in age-specific uptake from status quo in 2021, to 70% in 2070, followed by a 1% increase every year till 90% is reached; (6) LBC 2070: maintaining 3-yearly LBC-based screening, with a rapid linear increase in age-specific uptake from status quo in 2021, to 70% in 2070, followed by a 1% increase every year till 90% is reached; (7) Status quo: cytology-based screening with coverage of 26.6% in urban areas and 19.3% in rural areas according to the nationally representative survey.

**Table S2. Vaccination and screening scenarios**

| Scenario | Vaccination start year | Vaccination coverage | Screening scenario | Modality of screening from 2022 | Year when 70% screening coverage first achieved | Year when 90% screening coverage first achieved | Comparator (additional numbers) | Comparator (averted numbers) |
| --- | --- | --- | --- | --- | --- | --- | --- | --- |
| 0a (status quo) | No vaccination | 0% | Status quo | 3-yearly LBC-based | maintain status quo | maintain status quo | 1b | .. |
| 1a | 2022 | 90% | Status quo | 3-yearly LBC-based | maintain status quo | maintain status quo | 1b | 0a |
| 2a | 2023 | 90% | Status quo | 3-yearly LBC-based | maintain status quo | maintain status quo | 1b | 0a |
| 3a | 2024 | 90% | Status quo | 3-yearly LBC-based | maintain status quo | maintain status quo | 1b | 0a |
| 4a | 2025 | 90% | Status quo | 3-yearly LBC-based | maintain status quo | maintain status quo | 1b | 0a |
| 5a | 2026 | 90% | Status quo | 3-yearly LBC-based | maintain status quo | maintain status quo | 1b | 0a |
| 6a | 2027 | 90% | Status quo | 3-yearly LBC-based | maintain status quo | maintain status quo | 1b | 0a |
| 7a | 2028 | 90% | Status quo | 3-yearly LBC-based | maintain status quo | maintain status quo | 1b | 0a |
| 8a | 2029 | 90% | Status quo | 3-yearly LBC-based | maintain status quo | maintain status quo | 1b | 0a |
| 9a | 2030 | 90% | Status quo | 3-yearly LBC-based | maintain status quo | maintain status quo | 1b | 0a |
| 0b | No vaccination | 0% | HPV 2030 | 5-yearly HPV-based | 2030 | 2050 | 1b | 0a |
| 1b (no-delay) | 2022 | 90% | HPV 2030 | 5-yearly HPV-based | 2030 | 2050 | .. | 0a |
| 2b | 2023 | 90% | HPV 2030 | 5-yearly HPV-based | 2030 | 2050 | 1b | 0a |
| 3b | 2024 | 90% | HPV 2030 | 5-yearly HPV-based | 2030 | 2050 | 1b | 0a |
| 4b | 2025 | 90% | HPV 2030 | 5-yearly HPV-based | 2030 | 2050 | 1b | 0a |
| 5b | 2026 | 90% | HPV 2030 | 5-yearly HPV-based | 2030 | 2050 | 1b | 0a |
| 6b | 2027 | 90% | HPV 2030 | 5-yearly HPV-based | 2030 | 2050 | 1b | 0a |
| 7b | 2028 | 90% | HPV 2030 | 5-yearly HPV-based | 2030 | 2050 | 1b | 0a |
| 8b | 2029 | 90% | HPV 2030 | 5-yearly HPV-based | 2030 | 2050 | 1b | 0a |
| 9b | 2030 | 90% | HPV 2030 | 5-yearly HPV-based | 2030 | 2050 | 1b | 0a |
| 0c | No vaccination | 0% | LBC 2030 | 3-yearly LBC-based | 2030 | 2050 | 1b | 0a |
| 1c | 2022 | 90% | LBC 2030 | 3-yearly LBC-based | 2030 | 2050 | 1b | 0a |
| 2c | 2023 | 90% | LBC 2030 | 3-yearly LBC-based | 2030 | 2050 | 1b | 0a |
| 3c | 2024 | 90% | LBC 2030 | 3-yearly LBC-based | 2030 | 2050 | 1b | 0a |
| 4c | 2025 | 90% | LBC 2030 | 3-yearly LBC-based | 2030 | 2050 | 1b | 0a |
| 5c | 2026 | 90% | LBC 2030 | 3-yearly LBC-based | 2030 | 2050 | 1b | 0a |
| 6c | 2027 | 90% | LBC 2030 | 3-yearly LBC-based | 2030 | 2050 | 1b | 0a |
| 7c | 2028 | 90% | LBC 2030 | 3-yearly LBC-based | 2030 | 2050 | 1b | 0a |
| 8c | 2029 | 90% | LBC 2030 | 3-yearly LBC-based | 2030 | 2050 | 1b | 0a |
| 9c | 2030 | 90% | LBC 2030 | 3-yearly LBC-based | 2030 | 2050 | 1b | 0a |
| 0d | No vaccination | 0% | HPV 2050 | 5-yearly HPV-based | 2050 | 2070 | 1b | 0a |
| 1d | 2022 | 90% | HPV 2050 | 5-yearly HPV-based | 2050 | 2070 | 1b | 0a |
| 2d | 2023 | 90% | HPV 2050 | 5-yearly HPV-based | 2050 | 2070 | 1b | 0a |
| 3d | 2024 | 90% | HPV 2050 | 5-yearly HPV-based | 2050 | 2070 | 1b | 0a |
| 4d | 2025 | 90% | HPV 2050 | 5-yearly HPV-based | 2050 | 2070 | 1b | 0a |
| 5d | 2026 | 90% | HPV 2050 | 5-yearly HPV-based | 2050 | 2070 | 1b | 0a |
| 6d | 2027 | 90% | HPV 2050 | 5-yearly HPV-based | 2050 | 2070 | 1b | 0a |
| 7d | 2028 | 90% | HPV 2050 | 5-yearly HPV-based | 2050 | 2070 | 1b | 0a |
| 8d | 2029 | 90% | HPV 2050 | 5-yearly HPV-based | 2050 | 2070 | 1b | 0a |
| 9d | 2030 | 90% | HPV 2050 | 5-yearly HPV-based | 2050 | 2070 | 1b | 0a |
| 0e | No vaccination | 0% | LBC 2050 | 3-yearly LBC-based | 2050 | 2070 | 1b | 0a |
| 1e | 2022 | 90% | LBC 2050 | 3-yearly LBC-based | 2050 | 2070 | 1b | 0a |
| 2e | 2023 | 90% | LBC 2050 | 3-yearly LBC-based | 2050 | 2070 | 1b | 0a |
| 3e | 2024 | 90% | LBC 2050 | 3-yearly LBC-based | 2050 | 2070 | 1b | 0a |
| 4e | 2025 | 90% | LBC 2050 | 3-yearly LBC-based | 2050 | 2070 | 1b | 0a |
| 5e | 2026 | 90% | LBC 2050 | 3-yearly LBC-based | 2050 | 2070 | 1b | 0a |
| 6e | 2027 | 90% | LBC 2050 | 3-yearly LBC-based | 2050 | 2070 | 1b | 0a |
| 7e | 2028 | 90% | LBC 2050 | 3-yearly LBC-based | 2050 | 2070 | 1b | 0a |
| 8e | 2029 | 90% | LBC 2050 | 3-yearly LBC-based | 2050 | 2070 | 1b | 0a |
| 9e | 2030 | 90% | LBC 2050 | 3-yearly LBC-based | 2050 | 2070 | 1b | 0a |
| 0f | No vaccination | 0% | HPV 2070 | 5-yearly HPV-based | 2070 | 2090 | 1b | 0a |
| 1f | 2022 | 90% | HPV 2070 | 5-yearly HPV-based | 2070 | 2090 | 1b | 0a |
| 2f | 2023 | 90% | HPV 2070 | 5-yearly HPV-based | 2070 | 2090 | 1b | 0a |
| 3f | 2024 | 90% | HPV 2070 | 5-yearly HPV-based | 2070 | 2090 | 1b | 0a |
| 4f | 2025 | 90% | HPV 2070 | 5-yearly HPV-based | 2070 | 2090 | 1b | 0a |
| 5f | 2026 | 90% | HPV 2070 | 5-yearly HPV-based | 2070 | 2090 | 1b | 0a |
| 6f | 2027 | 90% | HPV 2070 | 5-yearly HPV-based | 2070 | 2090 | 1b | 0a |
| 7f | 2028 | 90% | HPV 2070 | 5-yearly HPV-based | 2070 | 2090 | 1b | 0a |
| 8f | 2029 | 90% | HPV 2070 | 5-yearly HPV-based | 2070 | 2090 | 1b | 0a |
| 9f | 2030 | 90% | HPV 2070 | 5-yearly HPV-based | 2070 | 2090 | 1b | 0a |
| 0g | No vaccination | 0% | LBC 2070 | 3-yearly LBC-based | 2070 | 2090 | 1b | 0a |
| 1g | 2022 | 90% | LBC 2070 | 3-yearly LBC-based | 2070 | 2090 | 1b | 0a |
| 2g | 2023 | 90% | LBC 2070 | 3-yearly LBC-based | 2070 | 2090 | 1b | 0a |
| 3g | 2024 | 90% | LBC 2070 | 3-yearly LBC-based | 2070 | 2090 | 1b | 0a |
| 4g | 2025 | 90% | LBC 2070 | 3-yearly LBC-based | 2070 | 2090 | 1b | 0a |
| 5g | 2026 | 90% | LBC 2070 | 3-yearly LBC-based | 2070 | 2090 | 1b | 0a |
| 6g | 2027 | 90% | LBC 2070 | 3-yearly LBC-based | 2070 | 2090 | 1b | 0a |
| 7g | 2028 | 90% | LBC 2070 | 3-yearly LBC-based | 2070 | 2090 | 1b | 0a |
| 8g | 2029 | 90% | LBC 2070 | 3-yearly LBC-based | 2070 | 2090 | 1b | 0a |
| 9g | 2030 | 90% | LBC 2070 | 3-yearly LBC-based | 2070 | 2090 | 1b | 0a |

Abbreviations: LBC, liquid-based cytology; HPV, human papillomavirus.


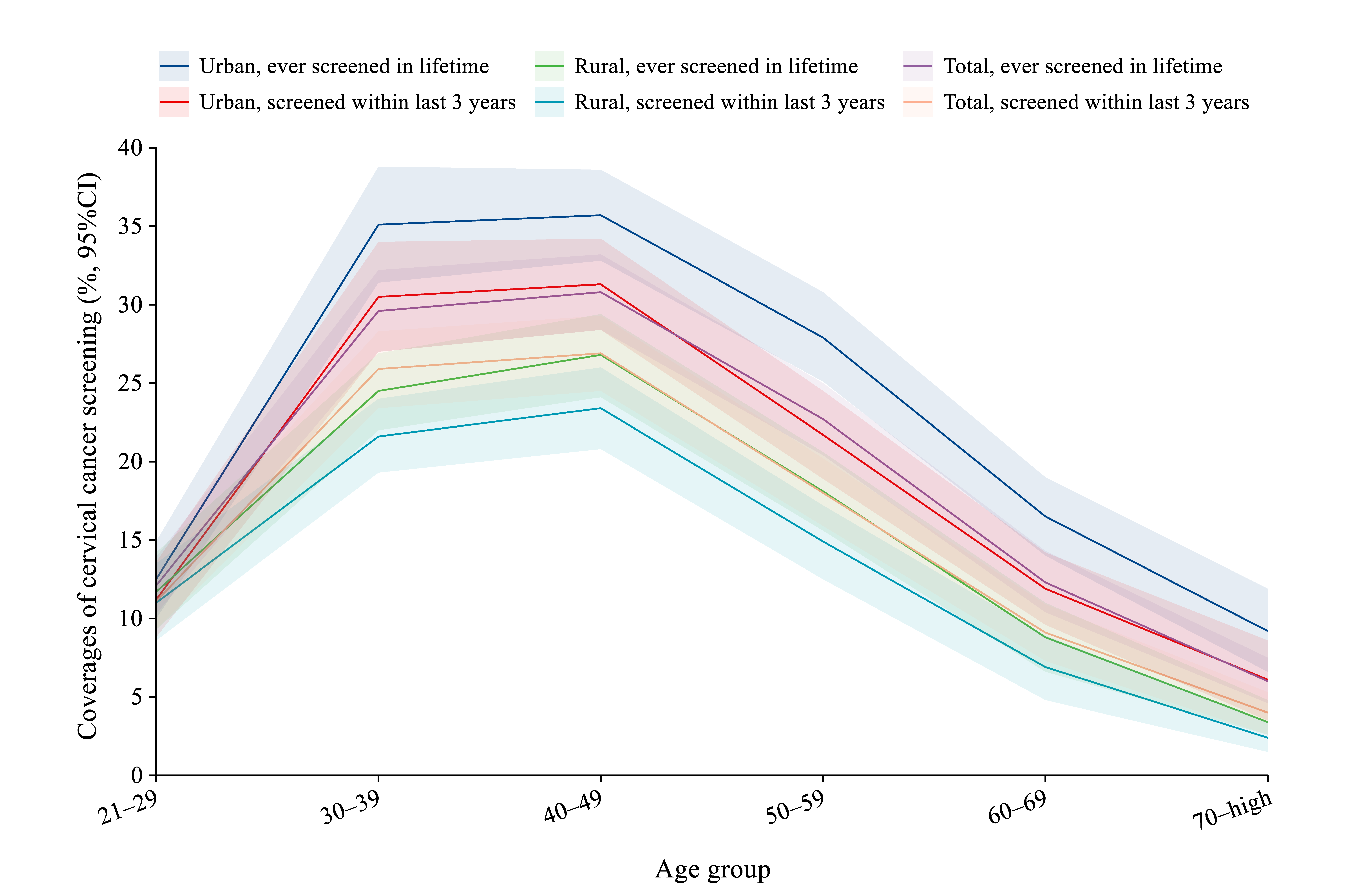


**Figure S2. Current cervical cancer screening coverage**


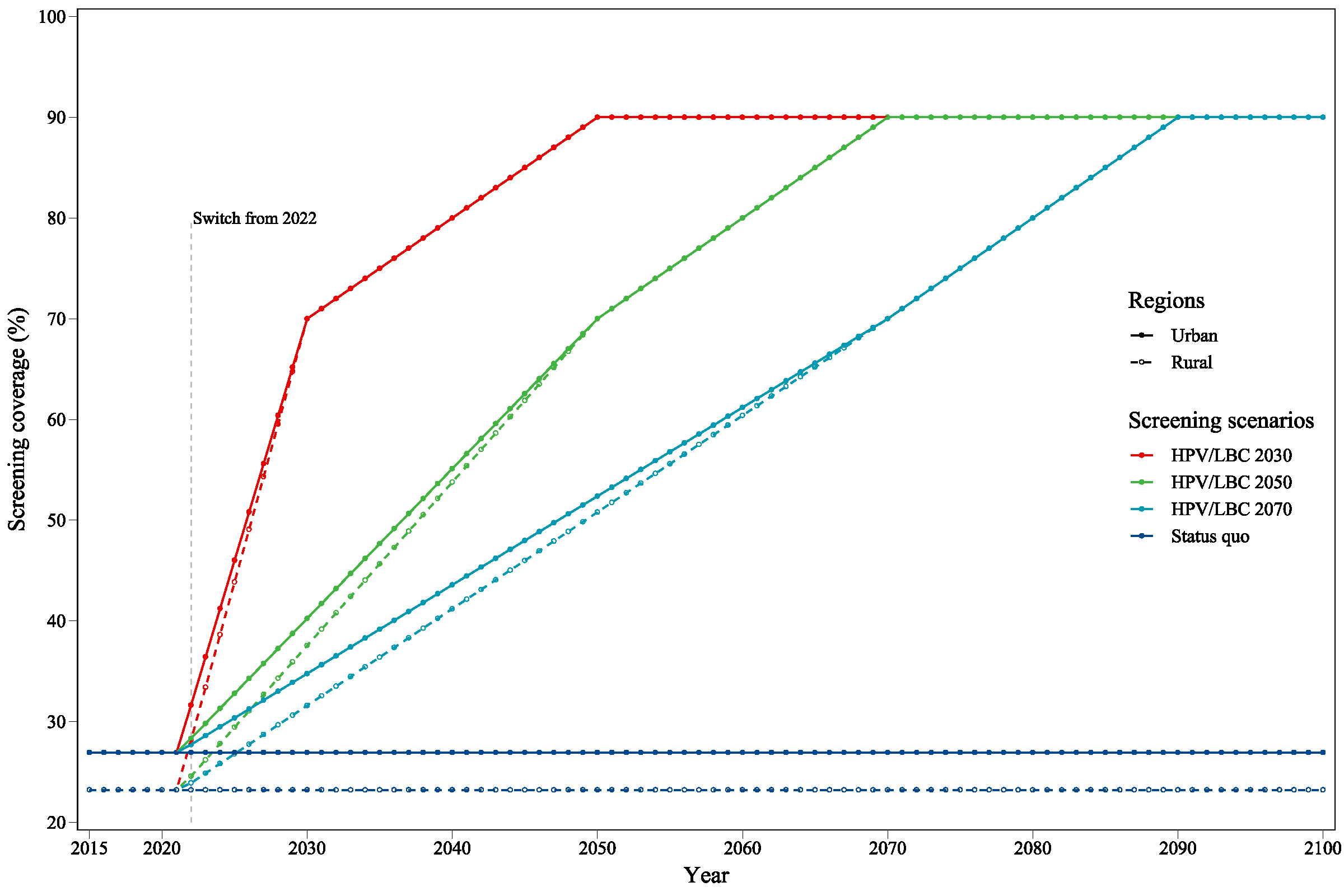


**Figure S3. Assumed coverage of HPV- and LBC-based cervical cancer screening among women at age 35-64 years in seven screening scenarios**

Abbreviations: HPV, human papillomavirus; LBC, liquid-based cytology.

Seven screening scenarios with different modalities and rates of increase in coverage for target population of women aged 35-64 years. “HPV 2030”, “HPV 2050”, and “HPV 2070” screening scenarios represent switching to HPV-based screening at 5-year intervals in 2022, with linearly increasing age-specific uptake from status quo in 2021, to 70% in 2030 (rapid), 2050 (moderate), and 2070 (gradual), respectively, followed by a 1% increase every year till 90% is reached. “LBC 2030”, “LBC 2050”, and “LBC 2070” screening scenarios represent maintaining LBC-based screening at 3-year intervals, with the corresponding rapid, moderate, and gradual increase in age-specific uptake. Status quo represents maintaining LBC-based screening with current coverage.

# **2. Results**

## **2.1 Main results**

The details of averted cases, deaths, costs, and effectiveness in different vaccination initiations compared with no vaccination are shown in Tables S3-S9. Because the domestic 2vHPV vaccine was assumed to have the same efficacy and cost-protection as the 4vHPV vaccine, the numbers of cases and deaths averted by both vaccines were the same. The additional cases and deaths are stratified by cohorts of whether or not they missed the vaccination due to delayed implementation (Figure S4). The cervical cancer elimination year of each scenario was shown in Table S10. The incremental costs of each scenario compared with no-delay scenario from each component of cervical cancer prevention and treatment was shown in Figure S5.

**Table S3. Estimated numbers of cases and deaths averted of each scenario with domestic 2vHPV and 4vHPV vaccines compared with the status quo scenario**

| **Screening scenarios** | **Initial years of vaccination** |  | **Cases** |  |  |  | **Deaths** |  |
| --- | --- | --- | --- | --- | --- | --- | --- | --- |
|  |  | **Averted vs. status quo** | **Additional vs. no-delay** | **Reduction in averted (%)** |  | **Averted vs. status quo** | **Additional vs. no-delay** | **Reduction in averted (%)** |
| **Domestic 2vHPV or 4vHPV** | | | | | | | | |
| HPV 2030 | No vaccination | 11,362,842 | 3,415,070 | 23.11 |  | 4,677,871 | 1,068,473 | 18.59 |
|  | 2022 (no-delay) | 14,777,912 | 0 | 0.00 |  | 5,746,344 | 0 | 0.00 |
|  | 2023 | 14,724,540 | 53,371 | 0.36 |  | 5,729,239 | 17,104 | 0.30 |
|  | 2024 | 14,668,788 | 109,124 | 0.74 |  | 5,711,497 | 34,847 | 0.61 |
|  | 2025 | 14,610,411 | 167,501 | 1.13 |  | 5,693,023 | 53,321 | 0.93 |
|  | 2026 | 14,556,226 | 221,685 | 1.50 |  | 5,675,800 | 70,543 | 1.23 |
|  | 2027 | 14,504,573 | 273,339 | 1.85 |  | 5,659,362 | 86,981 | 1.51 |
|  | 2028 | 14,450,890 | 327,021 | 2.21 |  | 5,642,385 | 103,959 | 1.81 |
|  | 2029 | 14,396,817 | 381,095 | 2.58 |  | 5,625,332 | 121,012 | 2.11 |
|  | 2030 | 14,343,857 | 434,054 | 2.94 |  | 5,608,634 | 137,710 | 2.40 |
|  | Each year of delay* | -54,257 | .. | .. |  | -17,214 | .. | .. |
| LBC 2030 | No vaccination | 8,982,327 | 5,795,584 | 39.22 |  | 4,209,099 | 1,537,244 | 26.75 |
|  | 2022 | 13,412,961 | 1,364,950 | 9.24 |  | 5,467,854 | 278,490 | 4.85 |
|  | 2023 | 13,344,686 | 1,433,225 | 9.70 |  | 5,447,999 | 298,345 | 5.19 |
|  | 2024 | 13,272,935 | 1,504,977 | 10.18 |  | 5,427,266 | 319,077 | 5.55 |
|  | 2025 | 13,197,626 | 1,580,286 | 10.69 |  | 5,405,622 | 340,722 | 5.93 |
|  | 2026 | 13,127,630 | 1,650,282 | 11.17 |  | 5,385,426 | 360,918 | 6.28 |
|  | 2027 | 13,060,713 | 1,717,198 | 11.62 |  | 5,366,099 | 380,245 | 6.62 |
|  | 2028 | 12,990,977 | 1,786,935 | 12.09 |  | 5,346,075 | 400,268 | 6.97 |
|  | 2029 | 12,920,658 | 1,857,253 | 12.57 |  | 5,325,937 | 420,407 | 7.32 |
|  | 2030 | 12,851,750 | 1,926,162 | 13.03 |  | 5,306,208 | 440,135 | 7.66 |
|  | Each year of delay* | -70,151 | .. | .. |  | -20,206 | .. | .. |
| HPV 2050 | No vaccination | 10,201,662 | 4,576,250 | 30.97 |  | 4,201,284 | 1,545,060 | 26.89 |
|  | 2022 | 13,701,359 | 1,076,552 | 7.28 |  | 5,294,742 | 451,602 | 7.86 |
|  | 2023 | 13,639,410 | 1,138,501 | 7.70 |  | 5,275,055 | 471,289 | 8.20 |
|  | 2024 | 13,575,073 | 1,202,839 | 8.14 |  | 5,254,730 | 491,614 | 8.56 |
|  | 2025 | 13,508,224 | 1,269,688 | 8.59 |  | 5,233,719 | 512,625 | 8.92 |
|  | 2026 | 13,446,694 | 1,331,218 | 9.01 |  | 5,214,307 | 532,037 | 9.26 |
|  | 2027 | 13,388,498 | 1,389,414 | 9.40 |  | 5,195,926 | 550,418 | 9.58 |
|  | 2028 | 13,328,506 | 1,449,405 | 9.81 |  | 5,177,083 | 569,261 | 9.91 |
|  | 2029 | 13,268,585 | 1,509,327 | 10.21 |  | 5,158,304 | 588,040 | 10.23 |
|  | 2030 | 13,210,396 | 1,567,516 | 10.61 |  | 5,140,069 | 606,275 | 10.55 |
|  | Each year of delay* | -61,370 | .. | .. |  | -19,334 | .. | .. |
| LBC 2050 | No vaccination | 7,934,952 | 6,842,960 | 46.31 |  | 3,758,403 | 1,987,941 | 34.59 |
|  | 2022 | 12,438,607 | 2,339,304 | 15.83 |  | 5,038,574 | 707,770 | 12.32 |
|  | 2023 | 12,362,844 | 2,415,068 | 16.34 |  | 5,016,466 | 729,878 | 12.70 |
|  | 2024 | 12,283,611 | 2,494,301 | 16.88 |  | 4,993,494 | 752,849 | 13.10 |
|  | 2025 | 12,200,904 | 2,577,008 | 17.44 |  | 4,969,647 | 776,697 | 13.52 |
|  | 2026 | 12,124,506 | 2,653,406 | 17.96 |  | 4,947,556 | 798,788 | 13.90 |
|  | 2027 | 12,051,901 | 2,726,010 | 18.45 |  | 4,926,552 | 819,792 | 14.27 |
|  | 2028 | 11,976,669 | 2,801,242 | 18.96 |  | 4,904,913 | 841,431 | 14.64 |
|  | 2029 | 11,901,281 | 2,876,631 | 19.47 |  | 4,883,290 | 863,053 | 15.02 |
|  | 2030 | 11,827,867 | 2,950,045 | 19.96 |  | 4,862,248 | 884,096 | 15.39 |
|  | Each year of delay* | -76,343 | .. | .. |  | -22,041 | .. | .. |
| HPV 2070 | No vaccination | 9,144,242 | 5,633,670 | 38.12 |  | 3,770,447 | 1,975,897 | 34.39 |
|  | 2022 | 12,924,637 | 1,853,274 | 12.54 |  | 4,957,901 | 788,443 | 13.72 |
|  | 2023 | 12,849,875 | 1,928,037 | 13.05 |  | 4,933,342 | 813,002 | 14.15 |
|  | 2024 | 12,771,975 | 2,005,936 | 13.57 |  | 4,907,937 | 838,407 | 14.59 |
|  | 2025 | 12,690,893 | 2,087,019 | 14.12 |  | 4,881,657 | 864,687 | 15.05 |
|  | 2026 | 12,616,183 | 2,161,729 | 14.63 |  | 4,857,414 | 888,930 | 15.47 |
|  | 2027 | 12,545,403 | 2,232,509 | 15.11 |  | 4,834,471 | 911,872 | 15.87 |
|  | 2028 | 12,472,326 | 2,305,586 | 15.60 |  | 4,810,940 | 935,404 | 16.28 |
|  | 2029 | 12,399,356 | 2,378,556 | 16.10 |  | 4,787,535 | 958,809 | 16.69 |
|  | 2030 | 12,328,539 | 2,449,373 | 16.57 |  | 4,764,867 | 981,477 | 17.08 |
|  | Each year of delay* | -74,512 | .. | .. |  | -24,129 | .. | .. |
| LBC 2070 | No vaccination | 6,973,181 | 7,804,730 | 52.81 |  | 3,344,802 | 2,401,542 | 41.79 |
|  | 2022 | 11,722,708 | 3,055,203 | 20.67 |  | 4,708,393 | 1,037,950 | 18.06 |
|  | 2023 | 11,635,728 | 3,142,183 | 21.26 |  | 4,681,890 | 1,064,454 | 18.52 |
|  | 2024 | 11,544,583 | 3,233,328 | 21.88 |  | 4,654,326 | 1,092,018 | 19.00 |
|  | 2025 | 11,449,362 | 3,328,550 | 22.52 |  | 4,625,720 | 1,120,624 | 19.50 |
|  | 2026 | 11,361,374 | 3,416,537 | 23.12 |  | 4,599,272 | 1,147,072 | 19.96 |
|  | 2027 | 11,277,704 | 3,500,208 | 23.69 |  | 4,574,160 | 1,172,184 | 20.40 |
|  | 2028 | 11,190,998 | 3,586,914 | 24.27 |  | 4,548,318 | 1,198,026 | 20.85 |
|  | 2029 | 11,104,147 | 3,673,764 | 24.86 |  | 4,522,547 | 1,223,796 | 21.30 |
|  | 2030 | 11,019,617 | 3,758,295 | 25.43 |  | 4,497,529 | 1,248,815 | 21.73 |
|  | Each year of delay* | -87,886 | .. | .. |  | -26,358 | .. | .. |
| Status quo | No vaccination (status quo) | 0 | 14,777,912 | 100.00 |  | 0 | 5,746,344 | 100.00 |
|  | 2022 | 7,571,605 | 7,206,307 | 48.76 |  | 2,682,227 | 3,064,116 | 53.32 |
|  | 2023 | 7,456,628 | 7,321,283 | 49.54 |  | 2,640,963 | 3,105,381 | 54.04 |
|  | 2024 | 7,335,097 | 7,442,815 | 50.36 |  | 2,597,594 | 3,148,749 | 54.80 |
|  | 2025 | 7,207,233 | 7,570,679 | 51.23 |  | 2,552,130 | 3,194,214 | 55.59 |
|  | 2026 | 7,088,191 | 7,689,721 | 52.04 |  | 2,509,699 | 3,236,645 | 56.33 |
|  | 2027 | 6,974,086 | 7,803,825 | 52.81 |  | 2,469,019 | 3,277,325 | 57.03 |
|  | 2028 | 6,855,019 | 7,922,892 | 53.61 |  | 2,426,731 | 3,319,613 | 57.77 |
|  | 2029 | 6,734,936 | 8,042,976 | 54.43 |  | 2,384,145 | 3,362,198 | 58.51 |
|  | 2030 | 6,617,240 | 8,160,671 | 55.22 |  | 2,342,406 | 3,403,938 | 59.24 |
|  | Each year of delay* | -119,296 | .. | .. |  | -42,478 | .. | .. |

Abbreviations: HPV, human papillomavirus; LBC, liquid-based cytology.

*The mean annual impact of an eight-year delay in large-scale vaccination was calculated under each screening scenario from figures for vaccination initiated in 2030 compared to vaccination initiated in 2022 divided by eight.

Cases and deaths averted in each scenario were estimated as compared with status quo scenario (no vaccination and status quo screening). Additional cases and deaths in each scenario were estimated as compared with no-delay scenario (vaccination initiated in 2022 and HPV 2030 screening). Reduction in averted (%) referred to the percentage reduction in cases and deaths averted for each scenario compared with the no-delay scenario. Seven screening scenarios with different modalities and rates of increase in coverage for target population of women aged 35-64 years. “HPV 2030”, “HPV 2050”, and “HPV 2070” screening scenarios represent switching to HPV-based screening at 5-year intervals in 2022, with linearly increasing age-specific uptake from status quo in 2021, to 70% in 2030 (rapid), 2050 (moderate), and 2070 (gradual), respectively, followed by a 1% increase every year till 90% is reached. “LBC 2030”, “LBC 2050”, and “LBC 2070” screening scenarios represent maintaining LBC-based screening at 3-year intervals, with the corresponding rapid, moderate, and gradual increase in age-specific uptake. Status quo represents maintaining LBC-based screening with current coverage.

**Table S4. Estimated numbers of cases and deaths averted of each scenario with imported 2vHPV vaccine compared with the status quo scenario**

| **Screening scenarios** | **Initial years of vaccination** |  | **Cases** |  |  |  | **Deaths** |  |
| --- | --- | --- | --- | --- | --- | --- | --- | --- |
|  |  | **Averted vs. status quo** | **Additional vs. no-delay** | **Reduction in averted (%)** |  | **Averted vs. status quo** | **Additional vs. no-delay** | **Reduction in averted (%)** |
| **Imported 2vHPV** |  |  |  |  |  |  |  |  |
| HPV 2030 | No vaccination | 11,362,842 | 3,830,272 | 25.21 |  | 4,677,871 | 1,222,036 | 20.71 |
|  | 2022 (no-delay) | 15,193,114 | 0 | 0.00 |  | 5,899,907 | 0 | 0.00 |
|  | 2023 | 15,135,525 | 57,589 | 0.38 |  | 5,881,160 | 18,747 | 0.32 |
|  | 2024 | 15,075,349 | 117,764 | 0.78 |  | 5,861,702 | 38,205 | 0.65 |
|  | 2025 | 15,012,311 | 180,803 | 1.19 |  | 5,841,427 | 58,480 | 0.99 |
|  | 2026 | 14,953,619 | 239,495 | 1.58 |  | 5,822,453 | 77,454 | 1.31 |
|  | 2027 | 14,897,538 | 295,576 | 1.95 |  | 5,804,290 | 95,617 | 1.62 |
|  | 2028 | 14,839,234 | 353,879 | 2.33 |  | 5,785,521 | 114,386 | 1.94 |
|  | 2029 | 14,780,441 | 412,673 | 2.72 |  | 5,766,642 | 133,266 | 2.26 |
|  | 2030 | 14,722,760 | 470,354 | 3.10 |  | 5,748,116 | 151,791 | 2.57 |
|  | Each year of delay* | -58,794 | .. | .. |  | -18,974 | .. | .. |
| LBC 2030 | No vaccination | 8,982,327 | 6,210,787 | 40.88 |  | 4,209,099 | 1,690,808 | 28.66 |
|  | 2022 | 13,970,148 | 1,222,966 | 8.05 |  | 5,652,645 | 247,262 | 4.19 |
|  | 2023 | 13,896,308 | 1,296,806 | 8.54 |  | 5,630,838 | 269,069 | 4.56 |
|  | 2024 | 13,818,683 | 1,374,431 | 9.05 |  | 5,608,056 | 291,851 | 4.95 |
|  | 2025 | 13,737,168 | 1,455,946 | 9.58 |  | 5,584,256 | 315,651 | 5.35 |
|  | 2026 | 13,661,167 | 1,531,947 | 10.08 |  | 5,561,964 | 337,943 | 5.73 |
|  | 2027 | 13,588,337 | 1,604,777 | 10.56 |  | 5,540,569 | 359,338 | 6.09 |
|  | 2028 | 13,512,412 | 1,680,702 | 11.06 |  | 5,518,392 | 381,515 | 6.47 |
|  | 2029 | 13,435,766 | 1,757,348 | 11.57 |  | 5,496,057 | 403,850 | 6.85 |
|  | 2030 | 13,360,524 | 1,832,590 | 12.06 |  | 5,474,130 | 425,777 | 7.22 |
|  | Each year of delay* | -76,203 | .. | .. |  | -22,314 | .. | .. |
| HPV 2050 | No vaccination | 10,201,662 | 4,991,452 | 32.85 |  | 4,201,284 | 1,698,624 | 28.79 |
|  | 2022 | 14,122,955 | 1,070,159 | 7.04 |  | 5,449,995 | 449,913 | 7.63 |
|  | 2023 | 14,056,125 | 1,136,988 | 7.48 |  | 5,428,480 | 471,427 | 7.99 |
|  | 2024 | 13,986,704 | 1,206,410 | 7.94 |  | 5,406,259 | 493,648 | 8.37 |
|  | 2025 | 13,914,545 | 1,278,569 | 8.42 |  | 5,383,270 | 516,637 | 8.76 |
|  | 2026 | 13,847,939 | 1,345,175 | 8.85 |  | 5,361,954 | 537,953 | 9.12 |
|  | 2027 | 13,784,806 | 1,408,308 | 9.27 |  | 5,341,713 | 558,194 | 9.46 |
|  | 2028 | 13,719,708 | 1,473,406 | 9.70 |  | 5,320,950 | 578,957 | 9.81 |
|  | 2029 | 13,654,617 | 1,538,497 | 10.13 |  | 5,300,228 | 599,679 | 10.16 |
|  | 2030 | 13,591,306 | 1,601,808 | 10.54 |  | 5,280,060 | 619,847 | 10.51 |
|  | Each year of delay* | -66,456 | .. | .. |  | -21,242 | .. | .. |
| LBC 2050 | No vaccination | 7,934,952 | 7,258,162 | 47.77 |  | 3,758,403 | 2,141,504 | 36.30 |
|  | 2022 | 13,001,972 | 2,191,141 | 14.42 |  | 5,224,951 | 674,956 | 11.44 |
|  | 2023 | 12,920,000 | 2,273,113 | 14.96 |  | 5,200,715 | 699,192 | 11.85 |
|  | 2024 | 12,834,253 | 2,358,861 | 15.53 |  | 5,175,523 | 724,384 | 12.28 |
|  | 2025 | 12,744,711 | 2,448,403 | 16.12 |  | 5,149,353 | 750,554 | 12.72 |
|  | 2026 | 12,661,755 | 2,531,358 | 16.66 |  | 5,125,021 | 774,887 | 13.13 |
|  | 2027 | 12,582,743 | 2,610,371 | 17.18 |  | 5,101,820 | 798,087 | 13.53 |
|  | 2028 | 12,500,848 | 2,692,266 | 17.72 |  | 5,077,907 | 822,000 | 13.93 |
|  | 2029 | 12,418,696 | 2,774,418 | 18.26 |  | 5,053,976 | 845,931 | 14.34 |
|  | 2030 | 12,338,561 | 2,854,553 | 18.79 |  | 5,030,638 | 869,269 | 14.73 |
|  | Each year of delay* | -82,926 | .. | .. |  | -24,289 | .. | .. |
| HPV 2070 | No vaccination | 9,144,242 | 6,048,872 | 39.81 |  | 3,770,447 | 2,129,460 | 36.09 |
|  | 2022 | 13,379,903 | 1,813,211 | 11.93 |  | 5,124,023 | 775,884 | 13.15 |
|  | 2023 | 13,298,753 | 1,894,361 | 12.47 |  | 5,097,064 | 802,843 | 13.61 |
|  | 2024 | 13,214,181 | 1,978,933 | 13.03 |  | 5,069,170 | 830,738 | 14.08 |
|  | 2025 | 13,126,125 | 2,066,989 | 13.60 |  | 5,040,300 | 859,607 | 14.57 |
|  | 2026 | 13,044,759 | 2,148,355 | 14.14 |  | 5,013,578 | 886,330 | 15.02 |
|  | 2027 | 12,967,510 | 2,225,604 | 14.65 |  | 4,988,224 | 911,683 | 15.45 |
|  | 2028 | 12,887,743 | 2,305,371 | 15.17 |  | 4,962,212 | 937,695 | 15.89 |
|  | 2029 | 12,808,019 | 2,385,095 | 15.70 |  | 4,936,310 | 963,598 | 16.33 |
|  | 2030 | 12,730,530 | 2,462,583 | 16.21 |  | 4,911,176 | 988,732 | 16.76 |
|  | Each year of delay* | -81,172 | .. | .. |  | -26,606 | .. | .. |
| LBC 2070 | No vaccination | 6,973,181 | 8,219,933 | 54.10 |  | 3,344,802 | 2,555,105 | 43.31 |
|  | 2022 | 12,318,329 | 2,874,785 | 18.92 |  | 4,905,103 | 994,804 | 16.86 |
|  | 2023 | 12,223,715 | 2,969,399 | 19.54 |  | 4,875,926 | 1,023,981 | 17.36 |
|  | 2024 | 12,124,546 | 3,068,568 | 20.20 |  | 4,845,574 | 1,054,333 | 17.87 |
|  | 2025 | 12,020,906 | 3,172,208 | 20.88 |  | 4,814,060 | 1,085,847 | 18.40 |
|  | 2026 | 11,924,856 | 3,268,258 | 21.51 |  | 4,784,820 | 1,115,087 | 18.90 |
|  | 2027 | 11,833,316 | 3,359,798 | 22.11 |  | 4,756,985 | 1,142,923 | 19.37 |
|  | 2028 | 11,738,438 | 3,454,676 | 22.74 |  | 4,728,332 | 1,171,575 | 19.86 |
|  | 2029 | 11,643,308 | 3,549,806 | 23.36 |  | 4,699,724 | 1,200,183 | 20.34 |
|  | 2030 | 11,550,573 | 3,642,541 | 23.97 |  | 4,671,898 | 1,228,009 | 20.81 |
|  | Each year of delay* | -95,969 | .. | .. |  | -29,151 | .. | .. |
| Status quo | No vaccination (status quo) | 0 | 15,193,114 | 100.00 |  | 0 | 5,899,907 | 100.00 |
|  | 2022 | 8,835,808 | 6,357,305 | 41.84 |  | 3,185,516 | 2,714,391 | 46.01 |
|  | 2023 | 8,708,173 | 6,484,941 | 42.68 |  | 3,139,053 | 2,760,854 | 46.79 |
|  | 2024 | 8,573,171 | 6,619,943 | 43.57 |  | 3,090,186 | 2,809,721 | 47.62 |
|  | 2025 | 8,431,025 | 6,762,089 | 44.51 |  | 3,038,911 | 2,860,996 | 48.49 |
|  | 2026 | 8,298,152 | 6,894,962 | 45.38 |  | 2,990,835 | 2,909,072 | 49.31 |
|  | 2027 | 8,170,386 | 7,022,728 | 46.22 |  | 2,944,578 | 2,955,329 | 50.09 |
|  | 2028 | 8,036,987 | 7,156,127 | 47.10 |  | 2,896,459 | 3,003,448 | 50.91 |
|  | 2029 | 7,902,238 | 7,290,876 | 47.99 |  | 2,847,914 | 3,051,993 | 51.73 |
|  | 2030 | 7,769,865 | 7,423,249 | 48.86 |  | 2,800,209 | 3,099,698 | 52.54 |
|  | Each year of delay* | -133,243 | .. | .. |  | -48,163 | .. | .. |

Abbreviations: HPV, human papillomavirus; LBC, liquid-based cytology.

*The mean annual impact of an eight-year delay in large-scale vaccination was calculated under each screening scenario from figures for vaccination initiated in 2030 compared to vaccination initiated in 2022 divided by eight.

Cases and deaths averted in each scenario were estimated as compared with status quo scenario (no vaccination and status quo screening). Additional cases and deaths in each scenario were estimated as compared with no-delay scenario (vaccination initiated in 2022 and HPV 2030 screening). Reduction in averted (%) referred to the percentage reduction in cases and deaths averted for each scenario compared with the no-delay scenario. Seven screening scenarios with different modalities and rates of increase in coverage for target population of women aged 35-64 years. “HPV 2030”, “HPV 2050”, and “HPV 2070” screening scenarios represent switching to HPV-based screening at 5-year intervals in 2022, with linearly increasing age-specific uptake from status quo in 2021, to 70% in 2030 (rapid), 2050 (moderate), and 2070 (gradual), respectively, followed by a 1% increase every year till 90% is reached. “LBC 2030”, “LBC 2050”, and “LBC 2070” screening scenarios represent maintaining LBC-based screening at 3-year intervals, with the corresponding rapid, moderate, and gradual increase in age-specific uptake. Status quo represents maintaining LBC-based screening with current coverage.

**Table S5. Estimated numbers of cases and deaths averted of each scenario with 9vHPV vaccine compared with the status quo scenario**

| **Screening scenarios** | **Initial years of vaccination** |  | **Cases** |  |  |  | **Deaths** |  |
| --- | --- | --- | --- | --- | --- | --- | --- | --- |
|  |  | **Averted vs. status quo** | **Additional vs. no-delay** | **Reduction in averted (%)** |  | **Averted vs. status quo** | **Additional vs. no-delay** | **Reduction in averted (%)** |
| **9vHPV** |  |  |  |  |  |  |  |  |
| HPV 2030 | No vaccination | 11,362,842 | 4,426,152 | 28.03 |  | 4,677,871 | 1,427,930 | 23.39 |
|  | 2022 (no-delay) | 15,788,994 | 0 | 0.00 |  | 6,105,801 | 0 | 0.00 |
|  | 2023 | 15,722,655 | 66,339 | 0.42 |  | 6,083,924 | 21,877 | 0.36 |
|  | 2024 | 15,653,222 | 135,772 | 0.86 |  | 6,061,177 | 44,624 | 0.73 |
|  | 2025 | 15,580,311 | 208,683 | 1.32 |  | 6,037,415 | 68,386 | 1.12 |
|  | 2026 | 15,512,514 | 276,480 | 1.75 |  | 6,015,201 | 90,600 | 1.48 |
|  | 2027 | 15,447,801 | 341,193 | 2.16 |  | 5,993,957 | 111,844 | 1.83 |
|  | 2028 | 15,380,388 | 408,606 | 2.59 |  | 5,971,960 | 133,841 | 2.19 |
|  | 2029 | 15,312,319 | 476,674 | 3.02 |  | 5,949,803 | 155,998 | 2.55 |
|  | 2030 | 15,245,507 | 543,486 | 3.44 |  | 5,928,051 | 177,750 | 2.91 |
|  | Each year of delay* | -67,936 | .. | .. |  | -22,219 | .. | .. |
| LBC 2030 | No vaccination | 8,982,327 | 6,806,666 | 43.11 |  | 4,209,099 | 1,896,701 | 31.06 |
|  | 2022 | 14,810,599 | 978,395 | 6.20 |  | 5,912,046 | 193,755 | 3.17 |
|  | 2023 | 14,724,699 | 1,064,295 | 6.74 |  | 5,886,375 | 219,426 | 3.59 |
|  | 2024 | 14,634,173 | 1,154,821 | 7.31 |  | 5,859,494 | 246,307 | 4.03 |
|  | 2025 | 14,538,847 | 1,250,147 | 7.92 |  | 5,831,331 | 274,470 | 4.50 |
|  | 2026 | 14,450,092 | 1,338,902 | 8.48 |  | 5,804,985 | 300,816 | 4.93 |
|  | 2027 | 14,365,115 | 1,423,878 | 9.02 |  | 5,779,720 | 326,081 | 5.34 |
|  | 2028 | 14,276,309 | 1,512,684 | 9.58 |  | 5,753,471 | 352,330 | 5.77 |
|  | 2029 | 14,186,523 | 1,602,471 | 10.15 |  | 5,726,995 | 378,806 | 6.20 |
|  | 2030 | 14,098,335 | 1,690,659 | 10.71 |  | 5,700,988 | 404,812 | 6.63 |
|  | Each year of delay* | -89,033 | .. | .. |  | -26,382 | .. | .. |
| HPV 2050 | No vaccination | 10,201,662 | 5,587,332 | 35.39 |  | 4,201,284 | 1,904,517 | 31.19 |
|  | 2022 | 14,740,301 | 1,048,693 | 6.64 |  | 5,661,691 | 444,110 | 7.27 |
|  | 2023 | 14,662,560 | 1,126,434 | 7.13 |  | 5,636,436 | 469,365 | 7.69 |
|  | 2024 | 14,581,699 | 1,207,295 | 7.65 |  | 5,610,315 | 495,486 | 8.11 |
|  | 2025 | 14,497,497 | 1,291,497 | 8.18 |  | 5,583,239 | 522,562 | 8.56 |
|  | 2026 | 14,419,914 | 1,369,080 | 8.67 |  | 5,558,169 | 547,632 | 8.97 |
|  | 2027 | 14,346,489 | 1,442,505 | 9.14 |  | 5,534,394 | 571,407 | 9.36 |
|  | 2028 | 14,270,676 | 1,518,318 | 9.62 |  | 5,509,970 | 595,831 | 9.76 |
|  | 2029 | 14,194,813 | 1,594,181 | 10.10 |  | 5,485,569 | 620,232 | 10.16 |
|  | 2030 | 14,121,030 | 1,667,963 | 10.56 |  | 5,461,818 | 643,983 | 10.55 |
|  | Each year of delay* | -77,409 | .. | .. |  | -24,984 | .. | .. |
| LBC 2050 | No vaccination | 7,934,952 | 7,854,042 | 49.74 |  | 3,758,403 | 2,347,398 | 38.45 |
|  | 2022 | 13,863,490 | 1,925,504 | 12.20 |  | 5,489,962 | 615,838 | 10.09 |
|  | 2023 | 13,767,323 | 2,021,671 | 12.80 |  | 5,461,261 | 644,540 | 10.56 |
|  | 2024 | 13,666,521 | 2,122,473 | 13.44 |  | 5,431,371 | 674,429 | 11.05 |
|  | 2025 | 13,561,018 | 2,227,976 | 14.11 |  | 5,400,250 | 705,551 | 11.56 |
|  | 2026 | 13,463,458 | 2,325,535 | 14.73 |  | 5,371,361 | 734,440 | 12.03 |
|  | 2027 | 13,370,659 | 2,418,335 | 15.32 |  | 5,343,851 | 761,950 | 12.48 |
|  | 2028 | 13,274,287 | 2,514,706 | 15.93 |  | 5,315,440 | 790,361 | 12.94 |
|  | 2029 | 13,177,517 | 2,611,477 | 16.54 |  | 5,286,978 | 818,823 | 13.41 |
|  | 2030 | 13,083,122 | 2,705,872 | 17.14 |  | 5,259,216 | 846,585 | 13.87 |
|  | Each year of delay* | -97,546 | .. | .. |  | -28,843 | .. | .. |
| HPV 2070 | No vaccination | 9,144,242 | 6,644,752 | 42.08 |  | 3,770,447 | 2,335,354 | 38.25 |
|  | 2022 | 14,079,809 | 1,709,185 | 10.83 |  | 5,362,837 | 742,964 | 12.17 |
|  | 2023 | 13,983,909 | 1,805,085 | 11.43 |  | 5,330,656 | 775,145 | 12.70 |
|  | 2024 | 13,883,787 | 1,905,206 | 12.07 |  | 5,297,308 | 808,493 | 13.24 |
|  | 2025 | 13,779,326 | 2,009,668 | 12.73 |  | 5,262,728 | 843,073 | 13.81 |
|  | 2026 | 13,682,989 | 2,106,005 | 13.34 |  | 5,230,778 | 875,023 | 14.33 |
|  | 2027 | 13,591,664 | 2,197,330 | 13.92 |  | 5,200,512 | 905,289 | 14.83 |
|  | 2028 | 13,497,208 | 2,291,786 | 14.52 |  | 5,169,416 | 936,385 | 15.34 |
|  | 2029 | 13,402,731 | 2,386,262 | 15.11 |  | 5,138,429 | 967,372 | 15.84 |
|  | 2030 | 13,310,921 | 2,478,072 | 15.69 |  | 5,108,368 | 997,433 | 16.34 |
|  | Each year of delay* | -96,111 | .. | .. |  | -31,809 | .. | .. |
| LBC 2070 | No vaccination | 6,973,181 | 8,815,812 | 55.84 |  | 3,344,802 | 2,760,999 | 45.22 |
|  | 2022 | 13,260,615 | 2,528,379 | 16.01 |  | 5,196,736 | 909,064 | 14.89 |
|  | 2023 | 13,148,119 | 2,640,874 | 16.73 |  | 5,161,649 | 944,152 | 15.46 |
|  | 2024 | 13,029,919 | 2,759,075 | 17.47 |  | 5,125,074 | 980,726 | 16.06 |
|  | 2025 | 12,906,084 | 2,882,910 | 18.26 |  | 5,087,013 | 1,018,788 | 16.69 |
|  | 2026 | 12,791,547 | 2,997,447 | 18.98 |  | 5,051,765 | 1,054,035 | 17.26 |
|  | 2027 | 12,682,532 | 3,106,462 | 19.67 |  | 5,018,259 | 1,087,542 | 17.81 |
|  | 2028 | 12,569,310 | 3,219,684 | 20.39 |  | 4,983,709 | 1,122,092 | 18.38 |
|  | 2029 | 12,455,675 | 3,333,319 | 21.11 |  | 4,949,183 | 1,156,618 | 18.94 |
|  | 2030 | 12,344,904 | 3,444,090 | 21.81 |  | 4,915,605 | 1,190,196 | 19.49 |
|  | Each year of delay* | -114,464 | .. | .. |  | -35,141 | .. | .. |
| Status quo | No vaccination (status quo) | 0 | 15,788,994 | 100.00 |  | 0 | 6,105,801 | 100.00 |
|  | 2022 | 10,883,532 | 4,905,462 | 31.07 |  | 3,984,208 | 2,121,593 | 34.75 |
|  | 2023 | 10,726,865 | 5,062,128 | 32.06 |  | 3,926,189 | 2,179,611 | 35.70 |
|  | 2024 | 10,560,387 | 5,228,606 | 33.12 |  | 3,864,899 | 2,240,902 | 36.70 |
|  | 2025 | 10,384,365 | 5,404,629 | 34.23 |  | 3,800,314 | 2,305,486 | 37.76 |
|  | 2026 | 10,220,179 | 5,568,814 | 35.27 |  | 3,739,884 | 2,365,917 | 38.75 |
|  | 2027 | 10,062,460 | 5,726,533 | 36.27 |  | 3,681,798 | 2,424,003 | 39.70 |
|  | 2028 | 9,897,177 | 5,891,817 | 37.32 |  | 3,621,155 | 2,484,646 | 40.69 |
|  | 2029 | 9,729,876 | 6,059,118 | 38.38 |  | 3,559,847 | 2,545,954 | 41.70 |
|  | 2030 | 9,565,430 | 6,223,564 | 39.42 |  | 3,499,561 | 2,606,239 | 42.68 |
|  | Each year of delay* | -164,763 | .. | .. |  | -60,581 | .. | .. |

Abbreviations: HPV, human papillomavirus; LBC, liquid-based cytology.

*The mean annual impact of an eight-year delay in large-scale vaccination was calculated under each screening scenario from figures for vaccination initiated in 2030 compared to vaccination initiated in 2022 divided by eight.

Cases and deaths averted in each scenario were estimated as compared with status quo scenario (no vaccination and status quo screening). Additional cases and deaths in each scenario were estimated as compared with no-delay scenario (vaccination initiated in 2022 and HPV 2030 screening). Reduction in averted (%) referred to the percentage reduction in cases and deaths averted for each scenario compared with the no-delay scenario. Seven screening scenarios with different modalities and rates of increase in coverage for target population of women aged 35-64 years. “HPV 2030”, “HPV 2050”, and “HPV 2070” screening scenarios represent switching to HPV-based screening at 5-year intervals in 2022, with linearly increasing age-specific uptake from status quo in 2021, to 70% in 2030 (rapid), 2050 (moderate), and 2070 (gradual), respectively, followed by a 1% increase every year till 90% is reached. “LBC 2030”, “LBC 2050”, and “LBC 2070” screening scenarios represent maintaining LBC-based screening at 3-year intervals, with the corresponding rapid, moderate, and gradual increase in age-specific uptake. Status quo represents maintaining LBC-based screening with current coverage.

**Table S6. Estimated numbers of net costs saved and QALYs gained of each scenario with domestic 2vHPV vaccine compared with the status quo scenario.**

| **Screening scenarios** | **Initial years of vaccination** | **Costs (million US$), 3% discount rate** | | | | |  | | **QALYs, 3% discount rate** | | | | | |  | | **Costs (million US$), 0% discount rate** | | | | | |  | | **QALYs, 0% discount rate** | | | | | |  |
| --- | --- | --- | --- | --- | --- | --- | --- | --- | --- | --- | --- | --- | --- | --- | --- | --- | --- | --- | --- | --- | --- | --- | --- | --- | --- | --- | --- | --- | --- | --- | --- |
|  |  | **Saved vs. status quo** | **Additional vs. no-delay** | | **Reduction in saved (%)** | |  | | **Gained vs. status quo** | **Reduced vs. no-delay** | | | **Reduction in gained (%)** | |  | | **Saved vs. status quo** | **Additional vs. no-delay** | | | **Reduction in saved (%)** | |  | | **Gained vs. status quo** | **Reduced vs. no-delay** | | | **Reduction in gained (%)** | |  |
| **Domestic 2vHPV** | |  | |  | |  | |  | | |  |  | |  | |  | | |  |  | |  | |  | | |  |  | |  | |
| HPV 2030 | No vaccination | 11,519 | 12,295 | | 51.63 | |  | | 13,347,085 | 2,090,496 | | | 13.54 | |  | | 111,448 | 104,529 | | | 48.40 | |  | | 100,084,279 | 24,597,995 | | | 19.73 | |  |
|  | 2022 (no-delay) | 23,814 | 0 | | 0.00 | |  | | 15,437,581 | 0 | | | 0.00 | |  | | 215,977 | 0 | | | 0.00 | |  | | 124,682,273 | 0 | | | 0.00 | |  |
|  | 2023 | 23,371 | 443 | | 1.86 | |  | | 15,360,755 | 76,826 | | | 0.50 | |  | | 214,407 | 1,570 | | | 0.73 | |  | | 124,313,217 | 369,056 | | | 0.30 | |  |
|  | 2024 | 22,906 | 908 | | 3.81 | |  | | 15,282,068 | 155,513 | | | 1.01 | |  | | 212,724 | 3,253 | | | 1.51 | |  | | 123,925,677 | 756,597 | | | 0.61 | |  |
|  | 2025 | 22,427 | 1,387 | | 5.82 | |  | | 15,201,406 | 236,176 | | | 1.53 | |  | | 210,949 | 5,028 | | | 2.33 | |  | | 123,517,998 | 1,164,275 | | | 0.93 | |  |
|  | 2026 | 21,991 | 1,823 | | 7.66 | |  | | 15,128,635 | 308,946 | | | 2.00 | |  | | 209,292 | 6,685 | | | 3.10 | |  | | 123,139,843 | 1,542,430 | | | 1.24 | |  |
|  | 2027 | 21,580 | 2,234 | | 9.38 | |  | | 15,061,062 | 376,519 | | | 2.44 | |  | | 207,695 | 8,283 | | | 3.83 | |  | | 122,779,025 | 1,903,249 | | | 1.53 | |  |
|  | 2028 | 21,162 | 2,652 | | 11.14 | |  | | 14,992,305 | 445,276 | | | 2.88 | |  | | 206,026 | 9,951 | | | 4.61 | |  | | 122,402,182 | 2,280,091 | | | 1.83 | |  |
|  | 2029 | 20,751 | 3,063 | | 12.86 | |  | | 14,924,614 | 512,967 | | | 3.32 | |  | | 204,343 | 11,635 | | | 5.39 | |  | | 122,021,114 | 2,661,160 | | | 2.13 | |  |
|  | 2030 | 20,359 | 3,455 | | 14.51 | |  | | 14,859,940 | 577,641 | | | 3.74 | |  | | 202,692 | 13,286 | | | 6.15 | |  | | 121,646,950 | 3,035,323 | | | 2.43 | |  |
|  | Each year of delay* | -432 | .. | | .. | |  | | -72,205 | .. | | | .. | |  | | -1,661 | .. | | | .. | |  | | -379,415 | .. | | | .. | |  |
| LBC 2030 | No vaccination | 3,919 | 19,895 | | 83.54 | |  | | 11,835,195 | 3,602,386 | | | 23.34 | |  | | 76,820 | 139,157 | | | 64.43 | |  | | 87,945,501 | 36,736,773 | | | 29.46 | |  |
|  | 2022 | 18,386 | 5,428 | | 22.79 | |  | | 14,414,585 | 1,022,997 | | | 6.63 | |  | | 198,275 | 17,703 | | | 8.20 | |  | | 118,516,249 | 6,166,024 | | | 4.95 | |  |
|  | 2023 | 17,863 | 5,951 | | 24.99 | |  | | 14,320,798 | 1,116,783 | | | 7.23 | |  | | 196,452 | 19,525 | | | 9.04 | |  | | 118,063,328 | 6,618,945 | | | 5.31 | |  |
|  | 2024 | 17,315 | 6,499 | | 27.29 | |  | | 14,224,088 | 1,213,494 | | | 7.86 | |  | | 194,502 | 21,476 | | | 9.94 | |  | | 117,584,226 | 7,098,047 | | | 5.69 | |  |
|  | 2025 | 16,752 | 7,062 | | 29.66 | |  | | 14,124,742 | 1,312,839 | | | 8.50 | |  | | 192,444 | 23,533 | | | 10.90 | |  | | 117,079,017 | 7,603,256 | | | 6.10 | |  |
|  | 2026 | 16,238 | 7,576 | | 31.81 | |  | | 14,034,945 | 1,402,637 | | | 9.09 | |  | | 190,524 | 25,453 | | | 11.79 | |  | | 116,609,429 | 8,072,844 | | | 6.47 | |  |
|  | 2027 | 15,755 | 8,059 | | 33.84 | |  | | 13,951,262 | 1,486,320 | | | 9.63 | |  | | 188,674 | 27,304 | | | 12.64 | |  | | 116,159,653 | 8,522,620 | | | 6.84 | |  |
|  | 2028 | 15,264 | 8,550 | | 35.90 | |  | | 13,865,874 | 1,571,707 | | | 10.18 | |  | | 186,740 | 29,238 | | | 13.54 | |  | | 115,688,449 | 8,993,824 | | | 7.21 | |  |
|  | 2029 | 14,782 | 9,032 | | 37.93 | |  | | 13,781,756 | 1,655,826 | | | 10.73 | |  | | 184,788 | 31,189 | | | 14.44 | |  | | 115,211,542 | 9,470,731 | | | 7.60 | |  |
|  | 2030 | 14,321 | 9,493 | | 39.86 | |  | | 13,701,371 | 1,736,210 | | | 11.25 | |  | | 182,874 | 33,103 | | | 15.33 | |  | | 114,743,095 | 9,939,178 | | | 7.97 | |  |
|  | Each year of delay* | -508 | .. | | .. | |  | | -89,152 | .. | | | .. | |  | | -1,925 | .. | | | .. | |  | | -471,644 | .. | | | .. | |  |
| HPV 2050 | No vaccination | 9,445 | 14,369 | | 60.34 | |  | | 9,831,116 | 5,606,465 | | | 36.32 | |  | | 101,440 | 114,538 | | | 53.03 | |  | | 89,189,024 | 35,493,249 | | | 28.47 | |  |
|  | 2022 | 22,126 | 1,688 | | 7.09 | |  | | 12,082,285 | 3,355,296 | | | 21.73 | |  | | 207,405 | 8,573 | | | 3.97 | |  | | 114,700,743 | 9,981,530 | | | 8.01 | |  |
|  | 2023 | 21,637 | 2,177 | | 9.14 | |  | | 11,987,452 | 3,450,129 | | | 22.35 | |  | | 205,685 | 10,292 | | | 4.77 | |  | | 114,242,675 | 10,439,598 | | | 8.37 | |  |
|  | 2024 | 21,129 | 2,685 | | 11.28 | |  | | 11,890,986 | 3,546,595 | | | 22.97 | |  | | 203,855 | 12,122 | | | 5.61 | |  | | 113,765,070 | 10,917,203 | | | 8.76 | |  |
|  | 2025 | 20,608 | 3,206 | | 13.46 | |  | | 11,793,090 | 3,644,491 | | | 23.61 | |  | | 201,936 | 14,042 | | | 6.50 | |  | | 113,267,913 | 11,414,360 | | | 9.15 | |  |
|  | 2026 | 20,136 | 3,678 | | 15.44 | |  | | 11,705,660 | 3,731,922 | | | 24.17 | |  | | 200,154 | 15,823 | | | 7.33 | |  | | 112,811,841 | 11,870,432 | | | 9.52 | |  |
|  | 2027 | 19,694 | 4,120 | | 17.30 | |  | | 11,625,233 | 3,812,348 | | | 24.70 | |  | | 198,446 | 17,531 | | | 8.12 | |  | | 112,381,105 | 12,301,168 | | | 9.87 | |  |
|  | 2028 | 19,248 | 4,566 | | 19.18 | |  | | 11,544,263 | 3,893,318 | | | 25.22 | |  | | 196,671 | 19,306 | | | 8.94 | |  | | 111,936,231 | 12,746,042 | | | 10.22 | |  |
|  | 2029 | 18,811 | 5,003 | | 21.01 | |  | | 11,465,476 | 3,972,105 | | | 25.73 | |  | | 194,889 | 21,088 | | | 9.76 | |  | | 111,491,791 | 13,190,482 | | | 10.58 | |  |
|  | 2030 | 18,396 | 5,418 | | 22.75 | |  | | 11,391,079 | 4,046,502 | | | 26.21 | |  | | 193,151 | 22,826 | | | 10.57 | |  | | 111,060,725 | 13,621,549 | | | 10.93 | |  |
|  | Each year of delay* | -466 | .. | | .. | |  | | -86,401 | .. | | | .. | |  | | -1,782 | .. | | | .. | |  | | -455,002 | .. | | | .. | |  |
| LBC 2050 | No vaccination | 3,060 | 20,754 | | 87.15 | |  | | 8,618,794 | 6,818,787 | | | 44.17 | |  | | 68,995 | 146,982 | | | 68.05 | |  | | 78,063,484 | 46,618,789 | | | 37.39 | |  |
|  | 2022 | 17,854 | 5,960 | | 25.03 | |  | | 11,333,455 | 4,104,126 | | | 26.59 | |  | | 191,698 | 24,280 | | | 11.24 | |  | | 109,405,700 | 15,276,574 | | | 12.25 | |  |
|  | 2023 | 17,291 | 6,523 | | 27.39 | |  | | 11,224,315 | 4,213,266 | | | 27.29 | |  | | 189,744 | 26,234 | | | 12.15 | |  | | 108,876,501 | 15,805,773 | | | 12.68 | |  |
|  | 2024 | 16,707 | 7,107 | | 29.85 | |  | | 11,112,463 | 4,325,118 | | | 28.02 | |  | | 187,664 | 28,313 | | | 13.11 | |  | | 108,320,360 | 16,361,914 | | | 13.12 | |  |
|  | 2025 | 16,108 | 7,706 | | 32.36 | |  | | 10,998,419 | 4,439,163 | | | 28.76 | |  | | 185,481 | 30,497 | | | 14.12 | |  | | 107,738,496 | 16,943,778 | | | 13.59 | |  |
|  | 2026 | 15,564 | 8,250 | | 34.64 | |  | | 10,896,152 | 4,541,429 | | | 29.42 | |  | | 183,451 | 32,527 | | | 15.06 | |  | | 107,202,338 | 17,479,935 | | | 14.02 | |  |
|  | 2027 | 15,055 | 8,759 | | 36.78 | |  | | 10,801,574 | 4,636,007 | | | 30.03 | |  | | 181,504 | 34,474 | | | 15.96 | |  | | 106,693,016 | 17,989,257 | | | 14.43 | |  |
|  | 2028 | 14,539 | 9,275 | | 38.95 | |  | | 10,705,848 | 4,731,734 | | | 30.65 | |  | | 179,477 | 36,501 | | | 16.90 | |  | | 106,163,886 | 18,518,388 | | | 14.85 | |  |
|  | 2029 | 14,034 | 9,780 | | 41.07 | |  | | 10,612,373 | 4,825,208 | | | 31.26 | |  | | 177,439 | 38,538 | | | 17.84 | |  | | 105,633,255 | 19,049,019 | | | 15.28 | |  |
|  | 2030 | 13,554 | 10,260 | | 43.08 | |  | | 10,523,827 | 4,913,754 | | | 31.83 | |  | | 175,449 | 40,528 | | | 18.77 | |  | | 105,116,812 | 19,565,461 | | | 15.69 | |  |
|  | Each year of delay* | -537 | .. | | .. | |  | | -101,204 | .. | | | .. | |  | | -2,031 | .. | | | .. | |  | | -536,111 | .. | | | .. | |  |
| HPV 2070 | No vaccination | 8,186 | 15,628 | | 65.63 | |  | | 7,685,815 | 7,751,766 | | | 50.21 | |  | | 92,477 | 123,500 | | | 57.18 | |  | | 79,353,585 | 45,328,688 | | | 36.36 | |  |
|  | 2022 | 21,788 | 2,026 | | 8.51 | |  | | 10,336,653 | 5,100,929 | | | 33.04 | |  | | 203,154 | 12,824 | | | 5.94 | |  | | 107,763,561 | 16,918,713 | | | 13.57 | |  |
|  | 2023 | 21,244 | 2,570 | | 10.79 | |  | | 10,218,087 | 5,219,494 | | | 33.81 | |  | | 201,214 | 14,764 | | | 6.84 | |  | | 107,176,230 | 17,506,044 | | | 14.04 | |  |
|  | 2024 | 20,680 | 3,134 | | 13.16 | |  | | 10,097,041 | 5,340,541 | | | 34.59 | |  | | 199,156 | 16,822 | | | 7.79 | |  | | 106,561,572 | 18,120,701 | | | 14.53 | |  |
|  | 2025 | 20,102 | 3,712 | | 15.59 | |  | | 9,973,937 | 5,463,644 | | | 35.39 | |  | | 196,998 | 18,979 | | | 8.79 | |  | | 105,920,368 | 18,761,905 | | | 15.05 | |  |
|  | 2026 | 19,579 | 4,235 | | 17.78 | |  | | 9,863,733 | 5,573,849 | | | 36.11 | |  | | 194,996 | 20,982 | | | 9.71 | |  | | 105,330,879 | 19,351,394 | | | 15.52 | |  |
|  | 2027 | 19,089 | 4,725 | | 19.84 | |  | | 9,762,072 | 5,675,509 | | | 36.76 | |  | | 193,079 | 22,898 | | | 10.60 | |  | | 104,772,652 | 19,909,621 | | | 15.97 | |  |
|  | 2028 | 18,594 | 5,220 | | 21.92 | |  | | 9,659,532 | 5,778,049 | | | 37.43 | |  | | 191,088 | 24,890 | | | 11.52 | |  | | 104,194,998 | 20,487,275 | | | 16.43 | |  |
|  | 2029 | 18,110 | 5,704 | | 23.95 | |  | | 9,559,736 | 5,877,845 | | | 38.07 | |  | | 189,091 | 26,887 | | | 12.45 | |  | | 103,617,940 | 21,064,334 | | | 16.89 | |  |
|  | 2030 | 17,651 | 6,163 | | 25.88 | |  | | 9,465,489 | 5,972,092 | | | 38.69 | |  | | 187,144 | 28,834 | | | 13.35 | |  | | 103,058,367 | 21,623,906 | | | 17.34 | |  |
|  | Each year of delay* | -517 | .. | | .. | |  | | -108,895 | .. | | | .. | |  | | -2,001 | .. | | | .. | |  | | -588,149 | .. | | | .. | |  |
| LBC 2070 | No vaccination | 2,517 | 21,297 | | 89.43 | |  | | 6,622,779 | 8,814,802 | | | 57.10 | |  | | 61,810 | 154,168 | | | 71.38 | |  | | 69,029,919 | 55,652,354 | | | 44.64 | |  |
|  | 2022 | 18,100 | 5,714 | | 24.00 | |  | | 9,686,067 | 5,751,515 | | | 37.26 | |  | | 188,662 | 27,315 | | | 12.65 | |  | | 102,899,899 | 21,782,374 | | | 17.47 | |  |
|  | 2023 | 17,490 | 6,324 | | 26.56 | |  | | 9,555,908 | 5,881,673 | | | 38.10 | |  | | 186,515 | 29,463 | | | 13.64 | |  | | 102,255,930 | 22,426,343 | | | 17.99 | |  |
|  | 2024 | 16,857 | 6,957 | | 29.21 | |  | | 9,422,281 | 6,015,301 | | | 38.97 | |  | | 184,234 | 31,744 | | | 14.70 | |  | | 101,578,021 | 23,104,252 | | | 18.53 | |  |
|  | 2025 | 16,209 | 7,605 | | 31.93 | |  | | 9,285,914 | 6,151,667 | | | 39.85 | |  | | 181,840 | 34,138 | | | 15.81 | |  | | 100,868,235 | 23,814,038 | | | 19.10 | |  |
|  | 2026 | 15,621 | 8,193 | | 34.40 | |  | | 9,163,523 | 6,274,059 | | | 40.64 | |  | | 179,615 | 36,362 | | | 16.84 | |  | | 100,213,808 | 24,468,466 | | | 19.62 | |  |
|  | 2027 | 15,071 | 8,743 | | 36.71 | |  | | 9,050,220 | 6,387,362 | | | 41.38 | |  | | 177,484 | 38,494 | | | 17.82 | |  | | 99,591,686 | 25,090,587 | | | 20.12 | |  |
|  | 2028 | 14,514 | 9,300 | | 39.05 | |  | | 8,935,542 | 6,502,040 | | | 42.12 | |  | | 175,266 | 40,711 | | | 18.85 | |  | | 98,945,515 | 25,736,758 | | | 20.64 | |  |
|  | 2029 | 13,969 | 9,845 | | 41.34 | |  | | 8,823,612 | 6,613,969 | | | 42.84 | |  | | 173,038 | 42,939 | | | 19.88 | |  | | 98,297,977 | 26,384,296 | | | 21.16 | |  |
|  | 2030 | 13,451 | 10,363 | | 43.52 | |  | | 8,717,629 | 6,719,952 | | | 43.53 | |  | | 170,864 | 45,114 | | | 20.89 | |  | | 97,668,226 | 27,014,047 | | | 21.67 | |  |
|  | Each year of delay* | -581 | .. | | .. | |  | | -121,055 | .. | | | .. | |  | | -2,225 | .. | | | .. | |  | | -653,959 | .. | | | .. | |  |
| Status quo | No vaccination (status quo) | 0 | 23,814 | | 100.00 | |  | | 0 | 15,437,581 | | | 100.00 | |  | | 0 | 215,977 | | | 100.00 | |  | | 0 | 124,682,273 | | | 100.00 | |  |
|  | 2022 | 19,275 | 4,539 | | 19.06 | |  | | 5,119,705 | 10,317,876 | | | 66.84 | |  | | 174,097 | 41,880 | | | 19.39 | |  | | 65,753,750 | 58,928,523 | | | 47.26 | |  |
|  | 2023 | 18,565 | 5,249 | | 22.04 | |  | | 4,932,198 | 10,505,384 | | | 68.05 | |  | | 171,473 | 44,505 | | | 20.61 | |  | | 64,777,649 | 59,904,625 | | | 48.05 | |  |
|  | 2024 | 17,830 | 5,984 | | 25.13 | |  | | 4,738,121 | 10,699,460 | | | 69.31 | |  | | 168,686 | 47,291 | | | 21.90 | |  | | 63,740,758 | 60,941,516 | | | 48.88 | |  |
|  | 2025 | 17,076 | 6,738 | | 28.29 | |  | | 4,538,585 | 10,898,996 | | | 70.60 | |  | | 165,751 | 50,226 | | | 23.26 | |  | | 62,645,736 | 62,036,537 | | | 49.76 | |  |
|  | 2026 | 16,391 | 7,423 | | 31.17 | |  | | 4,357,959 | 11,079,623 | | | 71.77 | |  | | 163,014 | 52,964 | | | 24.52 | |  | | 61,626,004 | 63,056,270 | | | 50.57 | |  |
|  | 2027 | 15,748 | 8,066 | | 33.87 | |  | | 4,189,325 | 11,248,256 | | | 72.86 | |  | | 160,383 | 55,595 | | | 25.74 | |  | | 60,647,114 | 64,035,159 | | | 51.36 | |  |
|  | 2028 | 15,096 | 8,718 | | 36.61 | |  | | 4,017,423 | 11,420,159 | | | 73.98 | |  | | 157,637 | 58,340 | | | 27.01 | |  | | 59,621,882 | 65,060,391 | | | 52.18 | |  |
|  | 2029 | 14,457 | 9,357 | | 39.29 | |  | | 3,848,392 | 11,589,189 | | | 75.07 | |  | | 154,868 | 61,109 | | | 28.29 | |  | | 58,585,505 | 66,096,768 | | | 53.01 | |  |
|  | 2030 | 13,846 | 9,968 | | 41.86 | |  | | 3,687,102 | 11,750,480 | | | 76.12 | |  | | 152,154 | 63,824 | | | 29.55 | |  | | 57,568,405 | 67,113,868 | | | 53.83 | |  |
|  | Each year of delay* | -679 | .. | | .. | |  | | -179,075 | .. | | | .. | |  | | -2,743 | .. | | | .. | |  | | -1,023,168 | .. | | | .. | |  |

Abbreviations: HPV, human papillomavirus; LBC, liquid-based cytology; QALY, quality-adjusted life-year.

*The mean annual impact of an eight-year delay in large-scale vaccination was calculated under each screening scenario from figures for vaccination initiated in 2030 compared to vaccination initiated in 2022 divided by eight.

Costs saved and QALYs gained in each scenario were estimated as compared with status quo scenario (no vaccination and status quo screening). Additional costs and reduced QALYs in each scenario were estimated as compared with no-delay scenario (vaccination initiated in 2022 and HPV 2030 screening). Reduction in saved/gained (%) referred to the percentage reduction in costs saved and QALYs gained for each scenario compared with the no-delay scenario. Seven screening scenarios with different modalities and rates of increase in coverage for target population of women aged 35-64 years. “HPV 2030”, “HPV 2050”, a nd “HPV 2070” screening scenarios represent switching to HPV-based screening at 5-year intervals in 2022, with linearly increasing age-specific uptake from status quo in 2021, to 70% in 2030 (rapid), 2050 (moderate), and 2070 (gradual), respectively, followed by a 1% increase every year till 90% is reached. “LBC 2030”, “LBC 2050”, and “LBC 2070” screening scenarios represent maintaining LBC-based screening at 3-year intervals, with the corresponding rapid, moderate, and gradual increase in age-specific uptake. Status quo represents maintaining LBC-based screening with current coverage.

**Table S7. Estimated numbers of net costs saved and QALYs gained of each scenario with imported 2vHPV vaccine compared with the status quo scenario.**

| **Screening scenarios** | **Initial years of vaccination** | **Costs (million US$), 3% discount rate** | | | | |  | | **QALYs, 3% discount rate** | | | | | |  | | **Costs (million US$), 0% discount rate** | | | | | |  | | **QALYs, 0% discount rate** | | | | | |  |
| --- | --- | --- | --- | --- | --- | --- | --- | --- | --- | --- | --- | --- | --- | --- | --- | --- | --- | --- | --- | --- | --- | --- | --- | --- | --- | --- | --- | --- | --- | --- | --- |
|  |  | **Saved vs. status quo** | **Additional vs. no-delay** | | **Reduction in saved (%)** | |  | | **Gained vs. status quo** | **Reduced vs. no-delay** | | | **Reduction in gained (%)** | |  | | **Saved vs. status quo** | **Additional vs. no-delay** | | | **Reduction in saved (%)** | |  | | **Gained vs. status quo** | **Reduced vs. no-delay** | | | **Reduction in gained (%)** | |  |
| **Imported 2vHPV** | |  | |  | |  | |  | | |  |  | |  | |  | | |  |  | |  | |  | | |  |  | |  | |
| HPV 2030 | No vaccination | 11,519 | 13,431 | | 53.83 | |  | | 13,347,085 | 2,258,384 | | | 14.47 | |  | | 111,448 | 122,529 | | | 52.37 | |  | | 100,084,279 | 27,140,884 | | | 21.33 | |  |
|  | 2022 (no-delay) | 24,951 | 0 | | 0.00 | |  | | 15,605,469 | 0 | | | 0.00 | |  | | 233,977 | 0 | | | 0.00 | |  | | 127,225,162 | 0 | | | 0.00 | |  |
|  | 2023 | 24,486 | 465 | | 1.86 | |  | | 15,524,133 | 81,335 | | | 0.52 | |  | | 232,228 | 1,749 | | | 0.75 | |  | | 126,831,258 | 393,905 | | | 0.31 | |  |
|  | 2024 | 23,997 | 953 | | 3.82 | |  | | 15,440,801 | 164,667 | | | 1.06 | |  | | 230,351 | 3,626 | | | 1.55 | |  | | 126,417,526 | 807,637 | | | 0.63 | |  |
|  | 2025 | 23,495 | 1,456 | | 5.83 | |  | | 15,355,352 | 250,117 | | | 1.60 | |  | | 228,370 | 5,607 | | | 2.40 | |  | | 125,982,161 | 1,243,001 | | | 0.98 | |  |
|  | 2026 | 23,033 | 1,918 | | 7.69 | |  | | 15,278,098 | 327,371 | | | 2.10 | |  | | 226,510 | 7,467 | | | 3.19 | |  | | 125,577,288 | 1,647,875 | | | 1.30 | |  |
|  | 2027 | 22,595 | 2,355 | | 9.44 | |  | | 15,206,240 | 399,228 | | | 2.56 | |  | | 224,710 | 9,268 | | | 3.96 | |  | | 125,190,212 | 2,034,951 | | | 1.60 | |  |
|  | 2028 | 22,151 | 2,800 | | 11.22 | |  | | 15,133,108 | 472,360 | | | 3.03 | |  | | 222,828 | 11,149 | | | 4.76 | |  | | 124,785,866 | 2,439,297 | | | 1.92 | |  |
|  | 2029 | 21,714 | 3,236 | | 12.97 | |  | | 15,061,058 | 544,411 | | | 3.49 | |  | | 220,927 | 13,050 | | | 5.58 | |  | | 124,376,651 | 2,848,511 | | | 2.24 | |  |
|  | 2030 | 21,296 | 3,655 | | 14.65 | |  | | 14,992,140 | 613,328 | | | 3.93 | |  | | 219,059 | 14,918 | | | 6.38 | |  | | 123,974,310 | 3,250,852 | | | 2.56 | |  |
|  | Each year of delay* | -457 | .. | | .. | |  | | -76,666 | .. | | | .. | |  | | -1,865 | .. | | | .. | |  | | -406,357 | .. | | | .. | |  |
| LBC 2030 | No vaccination | 3,919 | 21,031 | | 84.29 | |  | | 11,835,195 | 3,770,274 | | | 24.16 | |  | | 76,820 | 157,157 | | | 67.17 | |  | | 87,945,501 | 39,279,662 | | | 30.87 | |  |
|  | 2022 | 19,700 | 5,251 | | 21.05 | |  | | 14,628,118 | 977,351 | | | 6.26 | |  | | 218,460 | 15,517 | | | 6.63 | |  | | 121,797,120 | 5,428,043 | | | 4.27 | |  |
|  | 2023 | 19,150 | 5,801 | | 23.25 | |  | | 14,528,679 | 1,076,790 | | | 6.90 | |  | | 216,438 | 17,539 | | | 7.50 | |  | | 121,312,618 | 5,912,545 | | | 4.65 | |  |
|  | 2024 | 18,574 | 6,377 | | 25.56 | |  | | 14,426,109 | 1,179,360 | | | 7.56 | |  | | 214,272 | 19,705 | | | 8.42 | |  | | 120,800,006 | 6,425,156 | | | 5.05 | |  |
|  | 2025 | 17,982 | 6,969 | | 27.93 | |  | | 14,320,709 | 1,284,759 | | | 8.23 | |  | | 211,985 | 21,992 | | | 9.40 | |  | | 120,259,294 | 6,965,869 | | | 5.48 | |  |
|  | 2026 | 17,438 | 7,512 | | 30.11 | |  | | 14,225,235 | 1,380,234 | | | 8.84 | |  | | 209,840 | 24,137 | | | 10.32 | |  | | 119,755,399 | 7,469,763 | | | 5.87 | |  |
|  | 2027 | 16,925 | 8,026 | | 32.17 | |  | | 14,136,112 | 1,469,357 | | | 9.42 | |  | | 207,764 | 26,213 | | | 11.20 | |  | | 119,271,809 | 7,953,353 | | | 6.25 | |  |
|  | 2028 | 16,403 | 8,548 | | 34.26 | |  | | 14,045,152 | 1,560,316 | | | 10.00 | |  | | 205,594 | 28,383 | | | 12.13 | |  | | 118,765,093 | 8,460,070 | | | 6.65 | |  |
|  | 2029 | 15,890 | 9,061 | | 36.32 | |  | | 13,955,478 | 1,649,991 | | | 10.57 | |  | | 203,401 | 30,576 | | | 13.07 | |  | | 118,251,810 | 8,973,352 | | | 7.05 | |  |
|  | 2030 | 15,398 | 9,552 | | 38.28 | |  | | 13,869,682 | 1,735,786 | | | 11.12 | |  | | 201,246 | 32,732 | | | 13.99 | |  | | 117,746,931 | 9,478,231 | | | 7.45 | |  |
|  | Each year of delay* | -538 | .. | | .. | |  | | -94,804 | .. | | | .. | |  | | -2,152 | .. | | | .. | |  | | -506,274 | .. | | | .. | |  |
| HPV 2050 | No vaccination | 9,445 | 15,506 | | 62.15 | |  | | 9,831,116 | 5,774,353 | | | 37.00 | |  | | 101,440 | 132,538 | | | 56.65 | |  | | 89,189,024 | 38,036,138 | | | 29.90 | |  |
|  | 2022 | 23,278 | 1,672 | | 6.70 | |  | | 12,259,342 | 3,346,127 | | | 21.44 | |  | | 225,493 | 8,485 | | | 3.63 | |  | | 117,299,688 | 9,925,474 | | | 7.80 | |  |
|  | 2023 | 22,766 | 2,185 | | 8.76 | |  | | 12,158,966 | 3,446,503 | | | 22.09 | |  | | 223,585 | 10,392 | | | 4.44 | |  | | 116,811,199 | 10,413,963 | | | 8.19 | |  |
|  | 2024 | 22,232 | 2,718 | | 10.90 | |  | | 12,056,838 | 3,548,631 | | | 22.74 | |  | | 221,552 | 12,425 | | | 5.31 | |  | | 116,301,798 | 10,923,364 | | | 8.59 | |  |
|  | 2025 | 21,686 | 3,264 | | 13.08 | |  | | 11,953,171 | 3,652,298 | | | 23.40 | |  | | 219,418 | 14,560 | | | 6.22 | |  | | 115,771,416 | 11,453,746 | | | 9.00 | |  |
|  | 2026 | 21,187 | 3,763 | | 15.08 | |  | | 11,860,409 | 3,745,059 | | | 24.00 | |  | | 217,425 | 16,552 | | | 7.07 | |  | | 115,283,740 | 11,941,423 | | | 9.39 | |  |
|  | 2027 | 20,718 | 4,233 | | 16.96 | |  | | 11,774,950 | 3,830,519 | | | 24.55 | |  | | 215,507 | 18,470 | | | 7.89 | |  | | 114,822,339 | 12,402,824 | | | 9.75 | |  |
|  | 2028 | 20,243 | 4,707 | | 18.87 | |  | | 11,688,900 | 3,916,569 | | | 25.10 | |  | | 213,513 | 20,464 | | | 8.75 | |  | | 114,345,723 | 12,879,439 | | | 10.12 | |  |
|  | 2029 | 19,779 | 5,171 | | 20.73 | |  | | 11,605,112 | 4,000,357 | | | 25.63 | |  | | 211,508 | 22,469 | | | 9.60 | |  | | 113,869,193 | 13,355,970 | | | 10.50 | |  |
|  | 2030 | 19,337 | 5,613 | | 22.50 | |  | | 11,525,908 | 4,079,561 | | | 26.14 | |  | | 209,546 | 24,431 | | | 10.44 | |  | | 113,406,403 | 13,818,759 | | | 10.86 | |  |
|  | Each year of delay* | -493 | .. | | .. | |  | | -91,679 | .. | | | .. | |  | | -1,993 | .. | | | .. | |  | | -486,661 | .. | | | .. | |  |
| LBC 2050 | No vaccination | 3,060 | 21,891 | | 87.74 | |  | | 8,618,794 | 6,986,674 | | | 44.77 | |  | | 68,995 | 164,982 | | | 70.51 | |  | | 78,063,484 | 49,161,678 | | | 38.64 | |  |
|  | 2022 | 19,183 | 5,768 | | 23.12 | |  | | 11,555,124 | 4,050,345 | | | 25.95 | |  | | 211,970 | 22,007 | | | 9.41 | |  | | 112,736,764 | 14,488,398 | | | 11.39 | |  |
|  | 2023 | 18,592 | 6,358 | | 25.48 | |  | | 11,439,405 | 4,166,064 | | | 26.70 | |  | | 209,808 | 24,169 | | | 10.33 | |  | | 112,170,934 | 15,054,229 | | | 11.83 | |  |
|  | 2024 | 17,978 | 6,973 | | 27.95 | |  | | 11,320,783 | 4,284,686 | | | 27.46 | |  | | 207,504 | 26,473 | | | 11.31 | |  | | 111,576,217 | 15,648,945 | | | 12.30 | |  |
|  | 2025 | 17,348 | 7,602 | | 30.47 | |  | | 11,199,804 | 4,405,665 | | | 28.23 | |  | | 205,082 | 28,895 | | | 12.35 | |  | | 110,953,839 | 16,271,323 | | | 12.79 | |  |
|  | 2026 | 16,773 | 8,178 | | 32.78 | |  | | 11,091,103 | 4,514,366 | | | 28.93 | |  | | 202,820 | 31,158 | | | 13.32 | |  | | 110,378,964 | 16,846,198 | | | 13.24 | |  |
|  | 2027 | 16,232 | 8,719 | | 34.94 | |  | | 10,990,417 | 4,615,052 | | | 29.57 | |  | | 200,640 | 33,337 | | | 14.25 | |  | | 109,831,857 | 17,393,305 | | | 13.67 | |  |
|  | 2028 | 15,684 | 9,266 | | 37.14 | |  | | 10,888,489 | 4,716,980 | | | 30.23 | |  | | 198,371 | 35,607 | | | 15.22 | |  | | 109,263,383 | 17,961,780 | | | 14.12 | |  |
|  | 2029 | 15,148 | 9,803 | | 39.29 | |  | | 10,788,888 | 4,816,581 | | | 30.86 | |  | | 196,086 | 37,891 | | | 16.19 | |  | | 108,692,829 | 18,532,333 | | | 14.57 | |  |
|  | 2030 | 14,636 | 10,314 | | 41.34 | |  | | 10,694,433 | 4,911,036 | | | 31.47 | |  | | 193,848 | 40,129 | | | 17.15 | |  | | 108,136,790 | 19,088,372 | | | 15.00 | |  |
|  | Each year of delay* | -568 | .. | | .. | |  | | -107,586 | .. | | | .. | |  | | -2,265 | .. | | | .. | |  | | -574,997 | .. | | | .. | |  |
| HPV 2070 | No vaccination | 8,186 | 16,765 | | 67.19 | |  | | 7,685,815 | 7,919,654 | | | 50.75 | |  | | 92,477 | 141,500 | | | 60.48 | |  | | 79,353,585 | 47,871,577 | | | 37.63 | |  |
|  | 2022 | 23,000 | 1,951 | | 7.82 | |  | | 10,548,865 | 5,056,603 | | | 32.40 | |  | | 221,694 | 12,284 | | | 5.25 | |  | | 110,641,323 | 16,583,840 | | | 13.04 | |  |
|  | 2023 | 22,428 | 2,522 | | 10.11 | |  | | 10,422,787 | 5,182,682 | | | 33.21 | |  | | 219,545 | 14,432 | | | 6.17 | |  | | 110,011,601 | 17,213,561 | | | 13.53 | |  |
|  | 2024 | 21,835 | 3,115 | | 12.49 | |  | | 10,294,040 | 5,311,429 | | | 34.04 | |  | | 217,264 | 16,714 | | | 7.14 | |  | | 109,352,501 | 17,872,661 | | | 14.05 | |  |
|  | 2025 | 21,229 | 3,721 | | 14.91 | |  | | 10,163,074 | 5,442,395 | | | 34.87 | |  | | 214,870 | 19,107 | | | 8.17 | |  | | 108,664,801 | 18,560,361 | | | 14.59 | |  |
|  | 2026 | 20,676 | 4,275 | | 17.13 | |  | | 10,045,599 | 5,559,870 | | | 35.63 | |  | | 212,637 | 21,341 | | | 9.12 | |  | | 108,031,096 | 19,194,066 | | | 15.09 | |  |
|  | 2027 | 20,156 | 4,795 | | 19.22 | |  | | 9,937,066 | 5,668,402 | | | 36.32 | |  | | 210,490 | 23,487 | | | 10.04 | |  | | 107,429,954 | 19,795,208 | | | 15.56 | |  |
|  | 2028 | 19,630 | 5,320 | | 21.32 | |  | | 9,827,580 | 5,777,889 | | | 37.02 | |  | | 208,260 | 25,718 | | | 10.99 | |  | | 106,807,847 | 20,417,315 | | | 16.05 | |  |
|  | 2029 | 19,116 | 5,834 | | 23.38 | |  | | 9,720,954 | 5,884,515 | | | 37.71 | |  | | 206,019 | 27,959 | | | 11.95 | |  | | 106,185,938 | 21,039,225 | | | 16.54 | |  |
|  | 2030 | 18,627 | 6,324 | | 25.34 | |  | | 9,620,150 | 5,985,319 | | | 38.35 | |  | | 203,828 | 30,149 | | | 12.89 | |  | | 105,582,149 | 21,643,013 | | | 17.01 | |  |
|  | Each year of delay* | -547 | .. | | .. | |  | | -116,089 | .. | | | .. | |  | | -2,233 | .. | | | .. | |  | | -632,397 | .. | | | .. | |  |
| LBC 2070 | No vaccination | 2,517 | 22,434 | | 89.91 | |  | | 6,622,779 | 8,982,690 | | | 57.56 | |  | | 61,810 | 172,167 | | | 73.58 | |  | | 69,029,919 | 58,195,243 | | | 45.74 | |  |
|  | 2022 | 19,485 | 5,466 | | 21.91 | |  | | 9,939,560 | 5,665,908 | | | 36.31 | |  | | 209,373 | 24,605 | | | 10.52 | |  | | 106,484,917 | 20,740,246 | | | 16.30 | |  |
|  | 2023 | 18,844 | 6,107 | | 24.48 | |  | | 9,801,027 | 5,804,442 | | | 37.19 | |  | | 206,998 | 26,980 | | | 11.53 | |  | | 105,793,327 | 21,431,836 | | | 16.85 | |  |
|  | 2024 | 18,178 | 6,772 | | 27.14 | |  | | 9,658,767 | 5,946,702 | | | 38.11 | |  | | 204,473 | 29,505 | | | 12.61 | |  | | 105,065,207 | 22,159,955 | | | 17.42 | |  |
|  | 2025 | 17,497 | 7,454 | | 29.87 | |  | | 9,513,555 | 6,091,914 | | | 39.04 | |  | | 201,820 | 32,157 | | | 13.74 | |  | | 104,302,694 | 22,922,468 | | | 18.02 | |  |
|  | 2026 | 16,875 | 8,076 | | 32.37 | |  | | 9,382,958 | 6,222,510 | | | 39.87 | |  | | 199,343 | 34,634 | | | 14.80 | |  | | 103,597,953 | 23,627,210 | | | 18.57 | |  |
|  | 2027 | 16,290 | 8,660 | | 34.71 | |  | | 9,261,871 | 6,343,598 | | | 40.65 | |  | | 196,961 | 37,016 | | | 15.82 | |  | | 102,926,785 | 24,298,377 | | | 19.10 | |  |
|  | 2028 | 15,699 | 9,252 | | 37.08 | |  | | 9,139,293 | 6,466,176 | | | 41.44 | |  | | 194,481 | 39,496 | | | 16.88 | |  | | 102,229,615 | 24,995,548 | | | 19.65 | |  |
|  | 2029 | 15,119 | 9,831 | | 39.40 | |  | | 9,019,571 | 6,585,898 | | | 42.20 | |  | | 191,987 | 41,990 | | | 17.95 | |  | | 101,530,443 | 25,694,720 | | | 20.20 | |  |
|  | 2030 | 14,567 | 10,384 | | 41.62 | |  | | 8,906,084 | 6,699,384 | | | 42.93 | |  | | 189,546 | 44,431 | | | 18.99 | |  | | 100,849,619 | 26,375,543 | | | 20.73 | |  |
|  | Each year of delay* | -615 | .. | | .. | |  | | -129,185 | .. | | | .. | |  | | -2,478 | .. | | | .. | |  | | -704,412 | .. | | | .. | |  |
| Status quo | No vaccination (status quo) | 0 | 24,951 | | 100.00 | |  | | 0 | 15,605,469 | | | 100.00 | |  | | 0 | 233,977 | | | 100.00 | |  | | 0 | 127,225,162 | | | 100.00 | |  |
|  | 2022 | 21,125 | 3,826 | | 15.33 | |  | | 5,676,920 | 9,928,549 | | | 63.62 | |  | | 204,282 | 29,696 | | | 12.69 | |  | | 75,099,529 | 52,125,633 | | | 40.97 | |  |
|  | 2023 | 20,375 | 4,576 | | 18.34 | |  | | 5,474,747 | 10,130,722 | | | 64.92 | |  | | 201,363 | 32,614 | | | 13.94 | |  | | 74,033,550 | 53,191,613 | | | 41.81 | |  |
|  | 2024 | 19,599 | 5,352 | | 21.45 | |  | | 5,265,372 | 10,340,097 | | | 66.26 | |  | | 198,262 | 35,715 | | | 15.26 | |  | | 72,900,678 | 54,324,484 | | | 42.70 | |  |
|  | 2025 | 18,802 | 6,148 | | 24.64 | |  | | 5,049,976 | 10,555,493 | | | 67.64 | |  | | 194,994 | 38,983 | | | 16.66 | |  | | 71,703,645 | 55,521,517 | | | 43.64 | |  |
|  | 2026 | 18,073 | 6,877 | | 27.56 | |  | | 4,854,451 | 10,751,018 | | | 68.89 | |  | | 191,931 | 42,046 | | | 17.97 | |  | | 70,585,170 | 56,639,992 | | | 44.52 | |  |
|  | 2027 | 17,388 | 7,562 | | 30.31 | |  | | 4,671,503 | 10,933,966 | | | 70.06 | |  | | 188,975 | 45,002 | | | 19.23 | |  | | 69,508,683 | 57,716,479 | | | 45.37 | |  |
|  | 2028 | 16,693 | 8,257 | | 33.09 | |  | | 4,484,911 | 11,120,558 | | | 71.26 | |  | | 185,890 | 48,087 | | | 20.55 | |  | | 68,380,752 | 58,844,410 | | | 46.25 | |  |
|  | 2029 | 16,011 | 8,940 | | 35.83 | |  | | 4,301,223 | 11,304,246 | | | 72.44 | |  | | 182,774 | 51,204 | | | 21.88 | |  | | 67,239,106 | 59,986,056 | | | 47.15 | |  |
|  | 2030 | 15,358 | 9,593 | | 38.45 | |  | | 4,125,652 | 11,479,817 | | | 73.56 | |  | | 179,710 | 54,267 | | | 23.19 | |  | | 66,116,544 | 61,108,618 | | | 48.03 | |  |
|  | Each year of delay* | -721 | .. | | .. | |  | | -193,908 | .. | | | .. | |  | | -3,071 | .. | | | .. | |  | | -1,122,873 | .. | | | .. | |  |

Abbreviations: HPV, human papillomavirus; LBC, liquid-based cytology; QALY, quality-adjusted life-year.

*The mean annual impact of an eight-year delay in large-scale vaccination was calculated under each screening scenario from figures for vaccination initiated in 2030 compared to vaccination initiated in 2022 divided by eight.

Costs saved and QALYs gained in each scenario were estimated as compared with status quo scenario (no vaccination and status quo screening). Additional costs and reduced QALYs in each scenario were estimated as compared with no-delay scenario (vaccination initiated in 2022 and HPV 2030 screening). Reduction in saved/gained (%) referred to the percentage reduction in costs saved and QALYs gained for each scenario compared with the no-delay scenario. Seven screening scenarios with different modalities and rates of increase in coverage for target population of women aged 35-64 years. “HPV 2030”, “HPV 2050”, and “HPV 2070” screening scenarios represent switching to HPV-based screening at 5-year intervals in 2022, with linearly increasing age-specific uptake from status quo in 2021, to 70% in 2030 (rapid), 2050 (moderate), and 2070 (gradual), respectively, followed by a 1% increase every year till 90% is reached. “LBC 2030”, “LBC 2050”, and “LBC 2070” screening scenarios represent maintaining LBC-based screening at 3-year intervals, with the corresponding rapid, moderate, and gradual increase in age-specific uptake. Status quo represents maintaining LBC-based screening with current coverage.

**Table S8. Estimated numbers of net costs saved and QALYs gained of each scenario with 4vHPV vaccine compared with the status quo scenario.**

| **Screening scenarios** | **Initial years of vaccination** | **Costs (million US$), 3% discount rate** | | | | |  | | **QALYs, 3% discount rate** | | | | | |  | | **Costs (million US$), 0% discount rate** | | | | | |  | | **QALYs, 0% discount rate** | | | | | |  |
| --- | --- | --- | --- | --- | --- | --- | --- | --- | --- | --- | --- | --- | --- | --- | --- | --- | --- | --- | --- | --- | --- | --- | --- | --- | --- | --- | --- | --- | --- | --- | --- |
|  |  | **Saved vs. status quo** | **Additional vs. no-delay** | | **Reduction in saved (%)** | |  | | **Gained vs. status quo** | **Reduced vs. no-delay** | | | **Reduction in gained (%)** | |  | | **Saved vs. status quo** | **Additional vs. no-delay** | | | **Reduction in saved (%)** | |  | | **Gained vs. status quo** | **Reduced vs. no-delay** | | | **Reduction in gained (%)** | |  |
| **4vHPV** | |  | |  | |  | |  | | |  |  | |  | |  | | |  |  | |  | |  | | |  |  | |  | |
| HPV 2030 | No vaccination | 11,519 | 10,159 | | 46.86 | |  | | 13,347,085 | 2,090,496 | | | 13.54 | |  | | 111,448 | 99,011 | | | 47.05 | |  | | 100,084,279 | 24,597,995 | | | 19.73 | |  |
|  | 2022 (no-delay) | 21,678 | 0 | | 0.00 | |  | | 15,437,581 | 0 | | | 0.00 | |  | | 210,459 | 0 | | | 0.00 | |  | | 124,682,273 | 0 | | | 0.00 | |  |
|  | 2023 | 21,310 | 368 | | 1.70 | |  | | 15,360,755 | 76,826 | | | 0.50 | |  | | 208,966 | 1,493 | | | 0.71 | |  | | 124,313,217 | 369,056 | | | 0.30 | |  |
|  | 2024 | 20,924 | 754 | | 3.48 | |  | | 15,282,068 | 155,513 | | | 1.01 | |  | | 207,368 | 3,092 | | | 1.47 | |  | | 123,925,677 | 756,597 | | | 0.61 | |  |
|  | 2025 | 20,530 | 1,148 | | 5.30 | |  | | 15,201,406 | 236,176 | | | 1.53 | |  | | 205,684 | 4,775 | | | 2.27 | |  | | 123,517,998 | 1,164,275 | | | 0.93 | |  |
|  | 2026 | 20,167 | 1,511 | | 6.97 | |  | | 15,128,635 | 308,946 | | | 2.00 | |  | | 204,110 | 6,349 | | | 3.02 | |  | | 123,139,843 | 1,542,430 | | | 1.24 | |  |
|  | 2027 | 19,824 | 1,854 | | 8.55 | |  | | 15,061,062 | 376,519 | | | 2.44 | |  | | 202,592 | 7,867 | | | 3.74 | |  | | 122,779,025 | 1,903,249 | | | 1.53 | |  |
|  | 2028 | 19,478 | 2,200 | | 10.15 | |  | | 14,992,305 | 445,276 | | | 2.88 | |  | | 201,009 | 9,450 | | | 4.49 | |  | | 122,402,182 | 2,280,091 | | | 1.83 | |  |
|  | 2029 | 19,139 | 2,539 | | 11.71 | |  | | 14,924,614 | 512,967 | | | 3.32 | |  | | 199,414 | 11,046 | | | 5.25 | |  | | 122,021,114 | 2,661,160 | | | 2.13 | |  |
|  | 2030 | 18,815 | 2,863 | | 13.21 | |  | | 14,859,940 | 577,641 | | | 3.74 | |  | | 197,849 | 12,610 | | | 5.99 | |  | | 121,646,950 | 3,035,323 | | | 2.43 | |  |
|  | Each year of delay* | -358 | .. | | .. | |  | | -72,205 | .. | | | .. | |  | | -1,576 | .. | | | .. | |  | | -379,415 | .. | | | .. | |  |
| LBC 2030 | No vaccination | 3,919 | 17,759 | | 81.92 | |  | | 11,835,195 | 3,602,386 | | | 23.34 | |  | | 76,820 | 133,639 | | | 63.50 | |  | | 87,945,501 | 36,736,773 | | | 29.46 | |  |
|  | 2022 | 16,250 | 5,428 | | 25.04 | |  | | 14,414,585 | 1,022,997 | | | 6.63 | |  | | 192,757 | 17,703 | | | 8.41 | |  | | 118,516,249 | 6,166,024 | | | 4.95 | |  |
|  | 2023 | 15,801 | 5,877 | | 27.11 | |  | | 14,320,798 | 1,116,783 | | | 7.23 | |  | | 191,011 | 19,449 | | | 9.24 | |  | | 118,063,328 | 6,618,945 | | | 5.31 | |  |
|  | 2024 | 15,334 | 6,345 | | 29.27 | |  | | 14,224,088 | 1,213,494 | | | 7.86 | |  | | 189,145 | 21,314 | | | 10.13 | |  | | 117,584,226 | 7,098,047 | | | 5.69 | |  |
|  | 2025 | 14,854 | 6,824 | | 31.48 | |  | | 14,124,742 | 1,312,839 | | | 8.50 | |  | | 187,180 | 23,280 | | | 11.06 | |  | | 117,079,017 | 7,603,256 | | | 6.10 | |  |
|  | 2026 | 14,414 | 7,264 | | 33.51 | |  | | 14,034,945 | 1,402,637 | | | 9.09 | |  | | 185,342 | 25,117 | | | 11.93 | |  | | 116,609,429 | 8,072,844 | | | 6.47 | |  |
|  | 2027 | 14,000 | 7,678 | | 35.42 | |  | | 13,951,262 | 1,486,320 | | | 9.63 | |  | | 183,571 | 26,888 | | | 12.78 | |  | | 116,159,653 | 8,522,620 | | | 6.84 | |  |
|  | 2028 | 13,580 | 8,098 | | 37.35 | |  | | 13,865,874 | 1,571,707 | | | 10.18 | |  | | 181,723 | 28,736 | | | 13.65 | |  | | 115,688,449 | 8,993,824 | | | 7.21 | |  |
|  | 2029 | 13,169 | 8,509 | | 39.25 | |  | | 13,781,756 | 1,655,826 | | | 10.73 | |  | | 179,859 | 30,600 | | | 14.54 | |  | | 115,211,542 | 9,470,731 | | | 7.60 | |  |
|  | 2030 | 12,777 | 8,901 | | 41.06 | |  | | 13,701,371 | 1,736,210 | | | 11.25 | |  | | 178,032 | 32,428 | | | 15.41 | |  | | 114,743,095 | 9,939,178 | | | 7.97 | |  |
|  | Each year of delay* | -434 | .. | | .. | |  | | -89,152 | .. | | | .. | |  | | -1,841 | .. | | | .. | |  | | -471,644 | .. | | | .. | |  |
| HPV 2050 | No vaccination | 9,445 | 12,233 | | 56.43 | |  | | 9,831,116 | 5,606,465 | | | 36.32 | |  | | 101,440 | 109,020 | | | 51.80 | |  | | 89,189,024 | 35,493,249 | | | 28.47 | |  |
|  | 2022 | 19,990 | 1,688 | | 7.79 | |  | | 12,082,285 | 3,355,296 | | | 21.73 | |  | | 201,887 | 8,573 | | | 4.07 | |  | | 114,700,743 | 9,981,530 | | | 8.01 | |  |
|  | 2023 | 19,576 | 2,102 | | 9.70 | |  | | 11,987,452 | 3,450,129 | | | 22.35 | |  | | 200,244 | 10,216 | | | 4.85 | |  | | 114,242,675 | 10,439,598 | | | 8.37 | |  |
|  | 2024 | 19,147 | 2,531 | | 11.67 | |  | | 11,890,986 | 3,546,595 | | | 22.97 | |  | | 198,499 | 11,961 | | | 5.68 | |  | | 113,765,070 | 10,917,203 | | | 8.76 | |  |
|  | 2025 | 18,711 | 2,967 | | 13.69 | |  | | 11,793,090 | 3,644,491 | | | 23.61 | |  | | 196,671 | 13,788 | | | 6.55 | |  | | 113,267,913 | 11,414,360 | | | 9.15 | |  |
|  | 2026 | 18,312 | 3,366 | | 15.53 | |  | | 11,705,660 | 3,731,922 | | | 24.17 | |  | | 194,972 | 15,487 | | | 7.36 | |  | | 112,811,841 | 11,870,432 | | | 9.52 | |  |
|  | 2027 | 17,939 | 3,739 | | 17.25 | |  | | 11,625,233 | 3,812,348 | | | 24.70 | |  | | 193,344 | 17,116 | | | 8.13 | |  | | 112,381,105 | 12,301,168 | | | 9.87 | |  |
|  | 2028 | 17,564 | 4,114 | | 18.98 | |  | | 11,544,263 | 3,893,318 | | | 25.22 | |  | | 191,655 | 18,805 | | | 8.94 | |  | | 111,936,231 | 12,746,042 | | | 10.22 | |  |
|  | 2029 | 17,199 | 4,480 | | 20.66 | |  | | 11,465,476 | 3,972,105 | | | 25.73 | |  | | 189,961 | 20,499 | | | 9.74 | |  | | 111,491,791 | 13,190,482 | | | 10.58 | |  |
|  | 2030 | 16,852 | 4,826 | | 22.26 | |  | | 11,391,079 | 4,046,502 | | | 26.21 | |  | | 188,308 | 22,151 | | | 10.53 | |  | | 111,060,725 | 13,621,549 | | | 10.93 | |  |
|  | Each year of delay* | -392 | .. | | .. | |  | | -86,401 | .. | | | .. | |  | | -1,697 | .. | | | .. | |  | | -455,002 | .. | | | .. | |  |
| LBC 2050 | No vaccination | 3,060 | 18,618 | | 85.88 | |  | | 8,618,794 | 6,818,787 | | | 44.17 | |  | | 68,995 | 141,464 | | | 67.22 | |  | | 78,063,484 | 46,618,789 | | | 37.39 | |  |
|  | 2022 | 15,718 | 5,960 | | 27.49 | |  | | 11,333,455 | 4,104,126 | | | 26.59 | |  | | 186,180 | 24,280 | | | 11.54 | |  | | 109,405,700 | 15,276,574 | | | 12.25 | |  |
|  | 2023 | 15,230 | 6,448 | | 29.74 | |  | | 11,224,315 | 4,213,266 | | | 27.29 | |  | | 184,303 | 26,157 | | | 12.43 | |  | | 108,876,501 | 15,805,773 | | | 12.68 | |  |
|  | 2024 | 14,725 | 6,953 | | 32.07 | |  | | 11,112,463 | 4,325,118 | | | 28.02 | |  | | 182,308 | 28,151 | | | 13.38 | |  | | 108,320,360 | 16,361,914 | | | 13.12 | |  |
|  | 2025 | 14,210 | 7,468 | | 34.45 | |  | | 10,998,419 | 4,439,163 | | | 28.76 | |  | | 180,216 | 30,244 | | | 14.37 | |  | | 107,738,496 | 16,943,778 | | | 13.59 | |  |
|  | 2026 | 13,740 | 7,938 | | 36.62 | |  | | 10,896,152 | 4,541,429 | | | 29.42 | |  | | 178,269 | 32,191 | | | 15.30 | |  | | 107,202,338 | 17,479,935 | | | 14.02 | |  |
|  | 2027 | 13,299 | 8,379 | | 38.65 | |  | | 10,801,574 | 4,636,007 | | | 30.03 | |  | | 176,401 | 34,058 | | | 16.18 | |  | | 106,693,016 | 17,989,257 | | | 14.43 | |  |
|  | 2028 | 12,855 | 8,823 | | 40.70 | |  | | 10,705,848 | 4,731,734 | | | 30.65 | |  | | 174,460 | 36,000 | | | 17.11 | |  | | 106,163,886 | 18,518,388 | | | 14.85 | |  |
|  | 2029 | 12,422 | 9,256 | | 42.70 | |  | | 10,612,373 | 4,825,208 | | | 31.26 | |  | | 172,510 | 37,949 | | | 18.03 | |  | | 105,633,255 | 19,049,019 | | | 15.28 | |  |
|  | 2030 | 12,010 | 9,668 | | 44.60 | |  | | 10,523,827 | 4,913,754 | | | 31.83 | |  | | 170,606 | 39,853 | | | 18.94 | |  | | 105,116,812 | 19,565,461 | | | 15.69 | |  |
|  | Each year of delay* | -463 | .. | | .. | |  | | -101,204 | .. | | | .. | |  | | -1,947 | .. | | | .. | |  | | -536,111 | .. | | | .. | |  |
| HPV 2070 | No vaccination | 8,186 | 13,492 | | 62.24 | |  | | 7,685,815 | 7,751,766 | | | 50.21 | |  | | 92,477 | 117,982 | | | 56.06 | |  | | 79,353,585 | 45,328,688 | | | 36.36 | |  |
|  | 2022 | 19,653 | 2,026 | | 9.34 | |  | | 10,336,653 | 5,100,929 | | | 33.04 | |  | | 197,636 | 12,824 | | | 6.09 | |  | | 107,763,561 | 16,918,713 | | | 13.57 | |  |
|  | 2023 | 19,183 | 2,495 | | 11.51 | |  | | 10,218,087 | 5,219,494 | | | 33.81 | |  | | 195,773 | 14,687 | | | 6.98 | |  | | 107,176,230 | 17,506,044 | | | 14.04 | |  |
|  | 2024 | 18,698 | 2,980 | | 13.75 | |  | | 10,097,041 | 5,340,541 | | | 34.59 | |  | | 193,799 | 16,660 | | | 7.92 | |  | | 106,561,572 | 18,120,701 | | | 14.53 | |  |
|  | 2025 | 18,205 | 3,473 | | 16.02 | |  | | 9,973,937 | 5,463,644 | | | 35.39 | |  | | 191,733 | 18,726 | | | 8.90 | |  | | 105,920,368 | 18,761,905 | | | 15.05 | |  |
|  | 2026 | 17,755 | 3,923 | | 18.10 | |  | | 9,863,733 | 5,573,849 | | | 36.11 | |  | | 189,814 | 20,646 | | | 9.81 | |  | | 105,330,879 | 19,351,394 | | | 15.52 | |  |
|  | 2027 | 17,334 | 4,345 | | 20.04 | |  | | 9,762,072 | 5,675,509 | | | 36.76 | |  | | 187,976 | 22,483 | | | 10.68 | |  | | 104,772,652 | 19,909,621 | | | 15.97 | |  |
|  | 2028 | 16,910 | 4,768 | | 21.99 | |  | | 9,659,532 | 5,778,049 | | | 37.43 | |  | | 186,071 | 24,388 | | | 11.59 | |  | | 104,194,998 | 20,487,275 | | | 16.43 | |  |
|  | 2029 | 16,498 | 5,180 | | 23.90 | |  | | 9,559,736 | 5,877,845 | | | 38.07 | |  | | 184,162 | 26,298 | | | 12.50 | |  | | 103,617,940 | 21,064,334 | | | 16.89 | |  |
|  | 2030 | 16,107 | 5,571 | | 25.70 | |  | | 9,465,489 | 5,972,092 | | | 38.69 | |  | | 182,301 | 28,158 | | | 13.38 | |  | | 103,058,367 | 21,623,906 | | | 17.34 | |  |
|  | Each year of delay* | -443 | .. | | .. | |  | | -108,895 | .. | | | .. | |  | | -1,917 | .. | | | .. | |  | | -588,149 | .. | | | .. | |  |
| LBC 2070 | No vaccination | 2,517 | 19,161 | | 88.39 | |  | | 6,622,779 | 8,814,802 | | | 57.10 | |  | | 61,810 | 148,650 | | | 70.63 | |  | | 69,029,919 | 55,652,354 | | | 44.64 | |  |
|  | 2022 | 15,964 | 5,714 | | 26.36 | |  | | 9,686,067 | 5,751,515 | | | 37.26 | |  | | 183,144 | 27,315 | | | 12.98 | |  | | 102,899,899 | 21,782,374 | | | 17.47 | |  |
|  | 2023 | 15,428 | 6,250 | | 28.83 | |  | | 9,555,908 | 5,881,673 | | | 38.10 | |  | | 181,074 | 29,386 | | | 13.96 | |  | | 102,255,930 | 22,426,343 | | | 17.99 | |  |
|  | 2024 | 14,876 | 6,802 | | 31.38 | |  | | 9,422,281 | 6,015,301 | | | 38.97 | |  | | 178,878 | 31,582 | | | 15.01 | |  | | 101,578,021 | 23,104,252 | | | 18.53 | |  |
|  | 2025 | 14,312 | 7,366 | | 33.98 | |  | | 9,285,914 | 6,151,667 | | | 39.85 | |  | | 176,575 | 33,885 | | | 16.10 | |  | | 100,868,235 | 23,814,038 | | | 19.10 | |  |
|  | 2026 | 13,797 | 7,881 | | 36.35 | |  | | 9,163,523 | 6,274,059 | | | 40.64 | |  | | 174,433 | 36,026 | | | 17.12 | |  | | 100,213,808 | 24,468,466 | | | 19.62 | |  |
|  | 2027 | 13,316 | 8,362 | | 38.58 | |  | | 9,050,220 | 6,387,362 | | | 41.38 | |  | | 172,381 | 38,079 | | | 18.09 | |  | | 99,591,686 | 25,090,587 | | | 20.12 | |  |
|  | 2028 | 12,831 | 8,847 | | 40.81 | |  | | 8,935,542 | 6,502,040 | | | 42.12 | |  | | 170,249 | 40,210 | | | 19.11 | |  | | 98,945,515 | 25,736,758 | | | 20.64 | |  |
|  | 2029 | 12,357 | 9,321 | | 43.00 | |  | | 8,823,612 | 6,613,969 | | | 42.84 | |  | | 168,109 | 42,350 | | | 20.12 | |  | | 98,297,977 | 26,384,296 | | | 21.16 | |  |
|  | 2030 | 11,907 | 9,771 | | 45.07 | |  | | 8,717,629 | 6,719,952 | | | 43.53 | |  | | 166,021 | 44,438 | | | 21.11 | |  | | 97,668,226 | 27,014,047 | | | 21.67 | |  |
|  | Each year of delay* | -507 | .. | | .. | |  | | -121,055 | .. | | | .. | |  | | -2,140 | .. | | | .. | |  | | -653,959 | .. | | | .. | |  |
| Status quo | No vaccination (status quo) | 0 | 21,678 | | 100.00 | |  | | 0 | 15,437,581 | | | 100.00 | |  | | 0 | 210,459 | | | 100.00 | |  | | 0 | 124,682,273 | | | 100.00 | |  |
|  | 2022 | 17,139 | 4,539 | | 20.94 | |  | | 5,119,705 | 10,317,876 | | | 66.84 | |  | | 168,579 | 41,880 | | | 19.90 | |  | | 65,753,750 | 58,928,523 | | | 47.26 | |  |
|  | 2023 | 16,504 | 5,174 | | 23.87 | |  | | 4,932,198 | 10,505,384 | | | 68.05 | |  | | 166,032 | 44,428 | | | 21.11 | |  | | 64,777,649 | 59,904,625 | | | 48.05 | |  |
|  | 2024 | 15,849 | 5,829 | | 26.89 | |  | | 4,738,121 | 10,699,460 | | | 69.31 | |  | | 163,330 | 47,130 | | | 22.39 | |  | | 63,740,758 | 60,941,516 | | | 48.88 | |  |
|  | 2025 | 15,179 | 6,499 | | 29.98 | |  | | 4,538,585 | 10,898,996 | | | 70.60 | |  | | 160,487 | 49,973 | | | 23.74 | |  | | 62,645,736 | 62,036,537 | | | 49.76 | |  |
|  | 2026 | 14,567 | 7,111 | | 32.80 | |  | | 4,357,959 | 11,079,623 | | | 71.77 | |  | | 157,832 | 52,628 | | | 25.01 | |  | | 61,626,004 | 63,056,270 | | | 50.57 | |  |
|  | 2027 | 13,993 | 7,685 | | 35.45 | |  | | 4,189,325 | 11,248,256 | | | 72.86 | |  | | 155,280 | 55,179 | | | 26.22 | |  | | 60,647,114 | 64,035,159 | | | 51.36 | |  |
|  | 2028 | 13,413 | 8,265 | | 38.13 | |  | | 4,017,423 | 11,420,159 | | | 73.98 | |  | | 152,621 | 57,839 | | | 27.48 | |  | | 59,621,882 | 65,060,391 | | | 52.18 | |  |
|  | 2029 | 12,844 | 8,834 | | 40.75 | |  | | 3,848,392 | 11,589,189 | | | 75.07 | |  | | 149,939 | 60,520 | | | 28.76 | |  | | 58,585,505 | 66,096,768 | | | 53.01 | |  |
|  | 2030 | 12,302 | 9,376 | | 43.25 | |  | | 3,687,102 | 11,750,480 | | | 76.12 | |  | | 147,311 | 63,148 | | | 30.01 | |  | | 57,568,405 | 67,113,868 | | | 53.83 | |  |
|  | Each year of delay* | -605 | .. | | .. | |  | | -179,075 | .. | | | .. | |  | | -2,659 | .. | | | .. | |  | | -1,023,168 | .. | | | .. | |  |

Abbreviations: HPV, human papillomavirus; LBC, liquid-based cytology; QALY, quality-adjusted life-year.

*The mean annual impact of an eight-year delay in large-scale vaccination was calculated under each screening scenario from figures for vaccination initiated in 2030 compared to vaccination initiated in 2022 divided by eight.

Costs saved and QALYs gained in each scenario were estimated as compared with status quo scenario (no vaccination and status quo screening). Additional costs and reduced QALYs in each scenario were estimated as compared with no-delay scenario (vaccination initiated in 2022 and HPV 2030 screening). Reduction in saved/gained (%) referred to the percentage reduction in costs saved and QALYs gained for each scenario compared with the no-delay scenario. Seven screening scenarios with different modalities and rates of increase in coverage for target population of women aged 35-64 years. “HPV 2030”, “HPV 2050”, and “HPV 2070” screening scenarios represent switching to HPV-based screening at 5-year intervals in 2022, with linearly increasing age-specific uptake from status quo in 2021, to 70% in 2030 (rapid), 2050 (moderate), and 2070 (gradual), respectively, followed by a 1% increase every year till 90% is reached. “LBC 2030”, “LBC 2050”, and “LBC 2070” screening scenarios represent maintaining LBC-based screening at 3-year intervals, with the corresponding rapid, moderate, and gradual increase in age-specific uptake. Status quo represents maintaining LBC-based screening with current coverage.

**Table S9. Estimated numbers of net costs saved and QALYs gained of each scenario with 9vHPV vaccine compared with the status quo scenario.**

| **Screening scenarios** | **Initial years of vaccination** | **Costs (million US$), 3% discount rate** | | | | |  | | **QALYs, 3% discount rate** | | | | | |  | | **Costs (million US$), 0% discount rate** | | | | | |  | | **QALYs, 0% discount rate** | | | | | |  |
| --- | --- | --- | --- | --- | --- | --- | --- | --- | --- | --- | --- | --- | --- | --- | --- | --- | --- | --- | --- | --- | --- | --- | --- | --- | --- | --- | --- | --- | --- | --- | --- |
|  |  | **Saved vs. status quo** | **Additional vs. no-delay** | | **Reduction in saved (%)** | |  | | **Gained vs. status quo** | **Reduced vs. no-delay** | | | **Reduction in gained (%)** | |  | | **Saved vs. status quo** | **Additional vs. no-delay** | | | **Reduction in saved (%)** | |  | | **Gained vs. status quo** | **Reduced vs. no-delay** | | | **Reduction in gained (%)** | |  |
| **9vHPV** | |  | |  | |  | |  | | |  |  | |  | |  | | |  |  | |  | |  | | |  |  | |  | |
| HPV 2030 | No vaccination | 11,519 | 16,181 | | 58.41 | |  | | 13,347,085 | 2,634,265 | | | 16.48 | |  | | 111,448 | 155,284 | | | 58.22 | |  | | 100,084,279 | 31,316,761 | | | 23.83 | |  |
|  | 2022 (no-delay) | 27,701 | 0 | | 0.00 | |  | | 15,981,350 | 0 | | | 0.00 | |  | | 266,732 | 0 | | | 0.00 | |  | | 131,401,039 | 0 | | | 0.00 | |  |
|  | 2023 | 27,152 | 549 | | 1.98 | |  | | 15,886,820 | 94,530 | | | 0.59 | |  | | 264,538 | 2,194 | | | 0.82 | |  | | 130,948,326 | 452,713 | | | 0.34 | |  |
|  | 2024 | 26,564 | 1,136 | | 4.10 | |  | | 15,789,790 | 191,560 | | | 1.20 | |  | | 262,146 | 4,586 | | | 1.72 | |  | | 130,471,859 | 929,180 | | | 0.71 | |  |
|  | 2025 | 25,955 | 1,745 | | 6.30 | |  | | 15,690,098 | 291,252 | | | 1.82 | |  | | 259,601 | 7,131 | | | 2.67 | |  | | 129,969,280 | 1,431,759 | | | 1.09 | |  |
|  | 2026 | 25,394 | 2,307 | | 8.33 | |  | | 15,600,042 | 381,308 | | | 2.39 | |  | | 257,219 | 9,513 | | | 3.57 | |  | | 129,502,549 | 1,898,490 | | | 1.44 | |  |
|  | 2027 | 24,860 | 2,840 | | 10.25 | |  | | 15,516,313 | 465,037 | | | 2.91 | |  | | 254,908 | 11,824 | | | 4.43 | |  | | 129,056,742 | 2,344,297 | | | 1.78 | |  |
|  | 2028 | 24,316 | 3,385 | | 12.22 | |  | | 15,430,953 | 550,397 | | | 3.44 | |  | | 252,478 | 14,254 | | | 5.34 | |  | | 128,590,066 | 2,810,974 | | | 2.14 | |  |
|  | 2029 | 23,779 | 3,922 | | 14.16 | |  | | 15,346,765 | 634,585 | | | 3.97 | |  | | 250,016 | 16,716 | | | 6.27 | |  | | 128,117,169 | 3,283,870 | | | 2.50 | |  |
|  | 2030 | 23,264 | 4,437 | | 16.02 | |  | | 15,266,201 | 715,149 | | | 4.47 | |  | | 247,595 | 19,137 | | | 7.17 | |  | | 127,652,034 | 3,749,005 | | | 2.85 | |  |
|  | Each year of delay* | -555 | .. | | .. | |  | | -89,394 | .. | | | .. | |  | | -2,392 | .. | | | .. | |  | | -468,626 | .. | | | .. | |  |
| LBC 2030 | No vaccination | 3,919 | 23,781 | | 85.85 | |  | | 11,835,195 | 4,146,155 | | | 25.94 | |  | | 76,820 | 189,911 | | | 71.20 | |  | | 87,945,501 | 43,455,538 | | | 33.07 | |  |
|  | 2022 | 22,717 | 4,984 | | 17.99 | |  | | 15,109,835 | 871,515 | | | 5.45 | |  | | 254,388 | 12,344 | | | 4.63 | |  | | 127,328,434 | 4,072,605 | | | 3.10 | |  |
|  | 2023 | 22,073 | 5,628 | | 20.32 | |  | | 14,993,774 | 987,576 | | | 6.18 | |  | | 251,876 | 14,856 | | | 5.57 | |  | | 126,767,594 | 4,633,445 | | | 3.53 | |  |
|  | 2024 | 21,388 | 6,313 | | 22.79 | |  | | 14,873,788 | 1,107,562 | | | 6.93 | |  | | 249,146 | 17,585 | | | 6.59 | |  | | 126,172,650 | 5,228,389 | | | 3.98 | |  |
|  | 2025 | 20,679 | 7,022 | | 25.35 | |  | | 14,750,231 | 1,231,119 | | | 7.70 | |  | | 246,246 | 20,486 | | | 7.68 | |  | | 125,543,432 | 5,857,607 | | | 4.46 | |  |
|  | 2026 | 20,027 | 7,673 | | 27.70 | |  | | 14,638,405 | 1,342,945 | | | 8.40 | |  | | 243,530 | 23,202 | | | 8.70 | |  | | 124,957,907 | 6,443,132 | | | 4.90 | |  |
|  | 2027 | 19,409 | 8,292 | | 29.93 | |  | | 14,534,053 | 1,447,297 | | | 9.06 | |  | | 240,900 | 25,832 | | | 9.68 | |  | | 124,396,401 | 7,004,638 | | | 5.33 | |  |
|  | 2028 | 18,778 | 8,922 | | 32.21 | |  | | 14,427,348 | 1,554,002 | | | 9.72 | |  | | 238,134 | 28,598 | | | 10.72 | |  | | 123,806,619 | 7,594,420 | | | 5.78 | |  |
|  | 2029 | 18,157 | 9,544 | | 34.45 | |  | | 14,322,022 | 1,659,328 | | | 10.38 | |  | | 235,331 | 31,401 | | | 11.77 | |  | | 123,208,343 | 8,192,696 | | | 6.23 | |  |
|  | 2030 | 17,561 | 10,140 | | 36.60 | |  | | 14,221,200 | 1,760,150 | | | 11.01 | |  | | 232,575 | 34,157 | | | 12.81 | |  | | 122,619,593 | 8,781,446 | | | 6.68 | |  |
|  | Each year of delay* | -644 | .. | | .. | |  | | -111,079 | .. | | | .. | |  | | -2,727 | .. | | | .. | |  | | -588,605 | .. | | | .. | |  |
| HPV 2050 | No vaccination | 9,445 | 18,256 | | 65.90 | |  | | 9,831,116 | 6,150,234 | | | 38.48 | |  | | 101,440 | 165,292 | | | 61.97 | |  | | 89,189,024 | 42,212,015 | | | 32.12 | |  |
|  | 2022 | 26,048 | 1,653 | | 5.97 | |  | | 12,667,451 | 3,313,899 | | | 20.74 | |  | | 258,464 | 8,268 | | | 3.10 | |  | | 121,668,279 | 9,732,760 | | | 7.41 | |  |
|  | 2023 | 25,448 | 2,253 | | 8.13 | |  | | 12,550,326 | 3,431,024 | | | 21.47 | |  | | 256,087 | 10,645 | | | 3.99 | |  | | 121,102,509 | 10,298,530 | | | 7.84 | |  |
|  | 2024 | 24,813 | 2,888 | | 10.43 | |  | | 12,430,952 | 3,550,398 | | | 22.22 | |  | | 253,516 | 13,216 | | | 4.95 | |  | | 120,511,484 | 10,889,555 | | | 8.29 | |  |
|  | 2025 | 24,158 | 3,543 | | 12.79 | |  | | 12,309,575 | 3,671,775 | | | 22.98 | |  | | 250,796 | 15,936 | | | 5.97 | |  | | 119,894,948 | 11,506,091 | | | 8.76 | |  |
|  | 2026 | 23,558 | 4,143 | | 14.96 | |  | | 12,201,072 | 3,780,278 | | | 23.65 | |  | | 248,262 | 18,470 | | | 6.92 | |  | | 119,328,993 | 12,072,046 | | | 9.19 | |  |
|  | 2027 | 22,991 | 4,710 | | 17.00 | |  | | 12,101,170 | 3,880,180 | | | 24.28 | |  | | 245,817 | 20,915 | | | 7.84 | |  | | 118,794,167 | 12,606,872 | | | 9.59 | |  |
|  | 2028 | 22,415 | 5,286 | | 19.08 | |  | | 12,000,436 | 3,980,914 | | | 24.91 | |  | | 243,259 | 23,473 | | | 8.80 | |  | | 118,240,845 | 13,160,194 | | | 10.02 | |  |
|  | 2029 | 21,849 | 5,852 | | 21.12 | |  | | 11,902,267 | 4,079,083 | | | 25.52 | |  | | 240,678 | 26,054 | | | 9.77 | |  | | 117,687,158 | 13,713,881 | | | 10.44 | |  |
|  | 2030 | 21,310 | 6,391 | | 23.07 | |  | | 11,809,444 | 4,171,906 | | | 26.10 | |  | | 238,150 | 28,582 | | | 10.72 | |  | | 117,149,437 | 14,251,602 | | | 10.85 | |  |
|  | Each year of delay* | -592 | .. | | .. | |  | | -107,251 | .. | | | .. | |  | | -2,539 | .. | | | .. | |  | | -564,855 | .. | | | .. | |  |
| LBC 2050 | No vaccination | 3,060 | 24,641 | | 88.95 | |  | | 8,618,794 | 7,362,556 | | | 46.07 | |  | | 68,995 | 197,737 | | | 74.13 | |  | | 78,063,484 | 53,337,555 | | | 40.59 | |  |
|  | 2022 | 22,226 | 5,474 | | 19.76 | |  | | 12,066,176 | 3,915,174 | | | 24.50 | |  | | 248,131 | 18,601 | | | 6.97 | |  | | 118,445,084 | 12,955,955 | | | 9.86 | |  |
|  | 2023 | 21,538 | 6,163 | | 22.25 | |  | | 11,930,566 | 4,050,785 | | | 25.35 | |  | | 245,453 | 21,279 | | | 7.98 | |  | | 117,785,735 | 13,615,304 | | | 10.36 | |  |
|  | 2024 | 20,811 | 6,890 | | 24.87 | |  | | 11,791,269 | 4,190,081 | | | 26.22 | |  | | 242,562 | 24,170 | | | 9.06 | |  | | 117,091,148 | 14,309,892 | | | 10.89 | |  |
|  | 2025 | 20,062 | 7,639 | | 27.58 | |  | | 11,648,939 | 4,332,411 | | | 27.11 | |  | | 239,502 | 27,230 | | | 10.21 | |  | | 116,362,643 | 15,038,396 | | | 11.44 | |  |
|  | 2026 | 19,376 | 8,325 | | 30.05 | |  | | 11,521,188 | 4,460,162 | | | 27.91 | |  | | 236,649 | 30,083 | | | 11.28 | |  | | 115,690,895 | 15,710,144 | | | 11.96 | |  |
|  | 2027 | 18,728 | 8,973 | | 32.39 | |  | | 11,402,918 | 4,578,432 | | | 28.65 | |  | | 233,897 | 32,835 | | | 12.31 | |  | | 115,052,266 | 16,348,773 | | | 12.44 | |  |
|  | 2028 | 18,070 | 9,631 | | 34.77 | |  | | 11,282,992 | 4,698,358 | | | 29.40 | |  | | 231,014 | 35,718 | | | 13.39 | |  | | 114,387,380 | 17,013,659 | | | 12.95 | |  |
|  | 2029 | 17,423 | 10,278 | | 37.10 | |  | | 11,165,692 | 4,815,658 | | | 30.13 | |  | | 228,104 | 38,628 | | | 14.48 | |  | | 113,719,393 | 17,681,646 | | | 13.46 | |  |
|  | 2030 | 16,806 | 10,895 | | 39.33 | |  | | 11,054,420 | 4,926,930 | | | 30.83 | |  | | 225,251 | 41,480 | | | 15.55 | |  | | 113,068,351 | 18,332,688 | | | 13.95 | |  |
|  | Each year of delay* | -678 | .. | | .. | |  | | -126,469 | .. | | | .. | |  | | -2,860 | .. | | | .. | |  | | -672,092 | .. | | | .. | |  |
| HPV 2070 | No vaccination | 8,186 | 19,515 | | 70.45 | |  | | 7,685,815 | 8,295,535 | | | 51.91 | |  | | 92,477 | 174,255 | | | 65.33 | |  | | 79,353,585 | 52,047,454 | | | 39.61 | |  |
|  | 2022 | 25,838 | 1,863 | | 6.73 | |  | | 11,049,698 | 4,931,652 | | | 30.86 | |  | | 255,524 | 11,208 | | | 4.20 | |  | | 115,706,178 | 15,694,861 | | | 11.94 | |  |
|  | 2023 | 25,173 | 2,528 | | 9.13 | |  | | 10,901,411 | 5,079,939 | | | 31.79 | |  | | 252,861 | 13,871 | | | 5.20 | |  | | 114,968,375 | 16,432,664 | | | 12.51 | |  |
|  | 2024 | 24,474 | 3,227 | | 11.65 | |  | | 10,749,693 | 5,231,657 | | | 32.74 | |  | | 249,997 | 16,735 | | | 6.27 | |  | | 114,194,511 | 17,206,528 | | | 13.09 | |  |
|  | 2025 | 23,754 | 3,947 | | 14.25 | |  | | 10,595,078 | 5,386,272 | | | 33.70 | |  | | 246,972 | 19,759 | | | 7.41 | |  | | 113,385,344 | 18,015,695 | | | 13.71 | |  |
|  | 2026 | 23,095 | 4,606 | | 16.63 | |  | | 10,456,541 | 5,524,809 | | | 34.57 | |  | | 244,156 | 22,576 | | | 8.46 | |  | | 112,640,970 | 18,760,069 | | | 14.28 | |  |
|  | 2027 | 22,474 | 5,227 | | 18.87 | |  | | 10,328,626 | 5,652,724 | | | 35.37 | |  | | 241,444 | 25,288 | | | 9.48 | |  | | 111,935,616 | 19,465,423 | | | 14.81 | |  |
|  | 2028 | 21,844 | 5,857 | | 21.14 | |  | | 10,199,387 | 5,781,963 | | | 36.18 | |  | | 238,610 | 28,122 | | | 10.54 | |  | | 111,204,338 | 20,196,702 | | | 15.37 | |  |
|  | 2029 | 21,226 | 6,475 | | 23.38 | |  | | 10,073,413 | 5,907,937 | | | 36.97 | |  | | 235,754 | 30,978 | | | 11.61 | |  | | 110,472,629 | 20,928,410 | | | 15.93 | |  |
|  | 2030 | 20,636 | 7,065 | | 25.50 | |  | | 9,954,290 | 6,027,060 | | | 37.71 | |  | | 232,960 | 33,772 | | | 12.66 | |  | | 109,762,221 | 21,638,818 | | | 16.47 | |  |
|  | Each year of delay* | -650 | .. | | .. | |  | | -136,926 | .. | | | .. | |  | | -2,820 | .. | | | .. | |  | | -742,995 | .. | | | .. | |  |
| LBC 2070 | No vaccination | 2,517 | 25,184 | | 90.91 | |  | | 6,622,779 | 9,358,571 | | | 58.56 | |  | | 61,810 | 204,922 | | | 76.83 | |  | | 69,029,919 | 62,371,120 | | | 47.47 | |  |
|  | 2022 | 22,602 | 5,098 | | 18.41 | |  | | 10,536,866 | 5,444,485 | | | 34.07 | |  | | 246,407 | 20,325 | | | 7.62 | |  | | 112,845,446 | 18,555,593 | | | 14.12 | |  |
|  | 2023 | 21,857 | 5,843 | | 21.09 | |  | | 10,373,352 | 5,607,998 | | | 35.09 | |  | | 243,473 | 23,259 | | | 8.72 | |  | | 112,031,433 | 19,369,606 | | | 14.74 | |  |
|  | 2024 | 21,075 | 6,626 | | 23.92 | |  | | 10,205,063 | 5,776,287 | | | 36.14 | |  | | 240,318 | 26,414 | | | 9.90 | |  | | 111,172,216 | 20,228,823 | | | 15.39 | |  |
|  | 2025 | 20,269 | 7,431 | | 26.83 | |  | | 10,032,941 | 5,948,409 | | | 37.22 | |  | | 236,983 | 29,749 | | | 11.15 | |  | | 110,270,262 | 21,130,777 | | | 16.08 | |  |
|  | 2026 | 19,533 | 8,168 | | 29.49 | |  | | 9,878,316 | 6,103,034 | | | 38.19 | |  | | 233,875 | 32,857 | | | 12.32 | |  | | 109,438,097 | 21,962,942 | | | 16.71 | |  |
|  | 2027 | 18,838 | 8,863 | | 32.00 | |  | | 9,735,024 | 6,246,326 | | | 39.09 | |  | | 230,882 | 35,850 | | | 13.44 | |  | | 108,646,364 | 22,754,675 | | | 17.32 | |  |
|  | 2028 | 18,133 | 9,568 | | 34.54 | |  | | 9,589,720 | 6,391,630 | | | 39.99 | |  | | 227,750 | 38,982 | | | 14.61 | |  | | 107,822,238 | 23,578,801 | | | 17.94 | |  |
|  | 2029 | 17,440 | 10,261 | | 37.04 | |  | | 9,447,660 | 6,533,690 | | | 40.88 | |  | | 224,591 | 42,141 | | | 15.80 | |  | | 106,994,866 | 24,406,173 | | | 18.57 | |  |
|  | 2030 | 16,779 | 10,922 | | 39.43 | |  | | 9,312,959 | 6,668,391 | | | 41.73 | |  | | 221,497 | 45,235 | | | 16.96 | |  | | 106,189,114 | 25,211,925 | | | 19.19 | |  |
|  | Each year of delay* | -728 | .. | | .. | |  | | -152,988 | .. | | | .. | |  | | -3,114 | .. | | | .. | |  | | -832,041 | .. | | | .. | |  |
| Status quo | No vaccination (status quo) | 0 | 27,701 | | 100.00 | |  | | 0 | 15,981,350 | | | 100.00 | |  | | 0 | 266,732 | | | 100.00 | |  | | 0 | 131,401,039 | | | 100.00 | |  |
|  | 2022 | 24,742 | 2,959 | | 10.68 | |  | | 6,875,430 | 9,105,920 | | | 56.98 | |  | | 254,047 | 12,685 | | | 4.76 | |  | | 90,837,788 | 40,563,251 | | | 30.87 | |  |
|  | 2023 | 23,872 | 3,828 | | 13.82 | |  | | 6,631,998 | 9,349,352 | | | 58.50 | |  | | 250,437 | 16,295 | | | 6.11 | |  | | 89,555,119 | 41,845,920 | | | 31.85 | |  |
|  | 2024 | 22,966 | 4,735 | | 17.09 | |  | | 6,379,029 | 9,602,321 | | | 60.08 | |  | | 246,567 | 20,165 | | | 7.56 | |  | | 88,186,200 | 43,214,839 | | | 32.89 | |  |
|  | 2025 | 22,031 | 5,669 | | 20.47 | |  | | 6,117,997 | 9,863,353 | | | 61.72 | |  | | 242,468 | 24,264 | | | 9.10 | |  | | 86,734,222 | 44,666,818 | | | 33.99 | |  |
|  | 2026 | 21,175 | 6,525 | | 23.56 | |  | | 5,881,308 | 10,100,042 | | | 63.20 | |  | | 238,632 | 28,100 | | | 10.53 | |  | | 85,380,182 | 46,020,858 | | | 35.02 | |  |
|  | 2027 | 20,369 | 7,332 | | 26.47 | |  | | 5,659,911 | 10,321,439 | | | 64.58 | |  | | 234,933 | 31,799 | | | 11.92 | |  | | 84,078,049 | 47,322,990 | | | 36.01 | |  |
|  | 2028 | 19,550 | 8,151 | | 29.43 | |  | | 5,433,487 | 10,547,863 | | | 66.00 | |  | | 231,053 | 35,679 | | | 13.38 | |  | | 82,708,963 | 48,692,076 | | | 37.06 | |  |
|  | 2029 | 18,743 | 8,958 | | 32.34 | |  | | 5,210,209 | 10,771,141 | | | 67.40 | |  | | 227,125 | 39,607 | | | 14.85 | |  | | 81,320,514 | 50,080,525 | | | 38.11 | |  |
|  | 2030 | 17,970 | 9,730 | | 35.13 | |  | | 4,996,641 | 10,984,709 | | | 68.73 | |  | | 223,261 | 43,471 | | | 16.30 | |  | | 79,954,451 | 51,446,588 | | | 39.15 | |  |
|  | Each year of delay* | -846 | .. | | .. | |  | | -234,849 | .. | | | .. | |  | | -3,848 | .. | | | .. | |  | | -1,360,417 | .. | | | .. | |  |

Abbreviations: HPV, human papillomavirus; LBC, liquid-based cytology; QALY, quality-adjusted life-year.

*The mean annual impact of an eight-year delay in large-scale vaccination was calculated under each screening scenario from figures for vaccination initiated in 2030 compared to vaccination initiated in 2022 divided by eight.

Costs saved and QALYs gained in each scenario were estimated as compared with status quo scenario (no vaccination and status quo screening). Additional costs and reduced QALYs in each scenario were estimated as compared with no-delay scenario (vaccination initiated in 2022 and HPV 2030 screening). Reduction in saved/gained (%) referred to the percentage reduction in costs saved and QALYs gained for each scenario compared with the no-delay scenario. Seven screening scenarios with different modalities and rates of increase in coverage for target population of women aged 35-64 years. “HPV 2030”, “HPV 2050”, and “HPV 2070” screening scenarios represent switching to HPV-based screening at 5-year intervals in 2022, with linearly increasing age-specific uptake from status quo in 2021, to 70% in 2030 (rapid), 2050 (moderate), and 2070 (gradual), respectively, followed by a 1% increase every year till 90% is reached. “LBC 2030”, “LBC 2050”, and “LBC 2070” screening scenarios represent maintaining LBC-based screening at 3-year intervals, with the corresponding rapid, moderate, and gradual increase in age-specific uptake. Status quo represents maintaining LBC-based screening with current coverage.


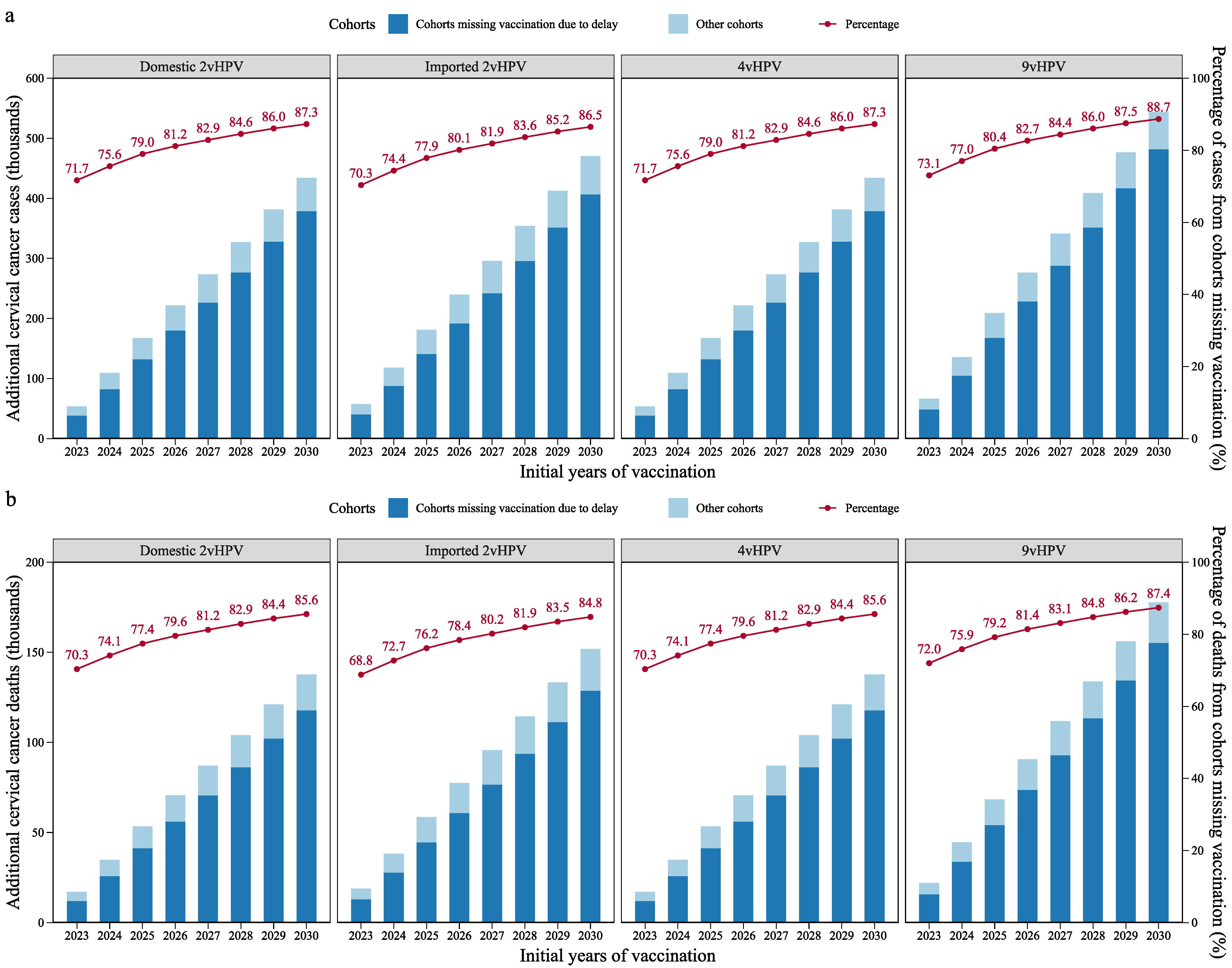


**Figure S4. Estimated numbers of additional (a) cervical cancer cases, and (b) deaths of scenarios with vaccination initated from 2023-2030 and HPV 2030 screening by cohorts compared with no-delay scenario.**

The dark blue bars indicate the additional cases and deaths that occurred in age cohorts who missed out on the vaccination due to delayed implementation, while the light blue bars indicate those additional cases and deaths that occurred in other age cohorts who would be protected through herd effects. HPV 2030 screening represents switching to HPV-based screening at 5-year intervals in 2022, with linearly increasing age-specific uptake from status quo in 2021 to 70% in 2030, followed by a 1% increase every year till 90% is reached.


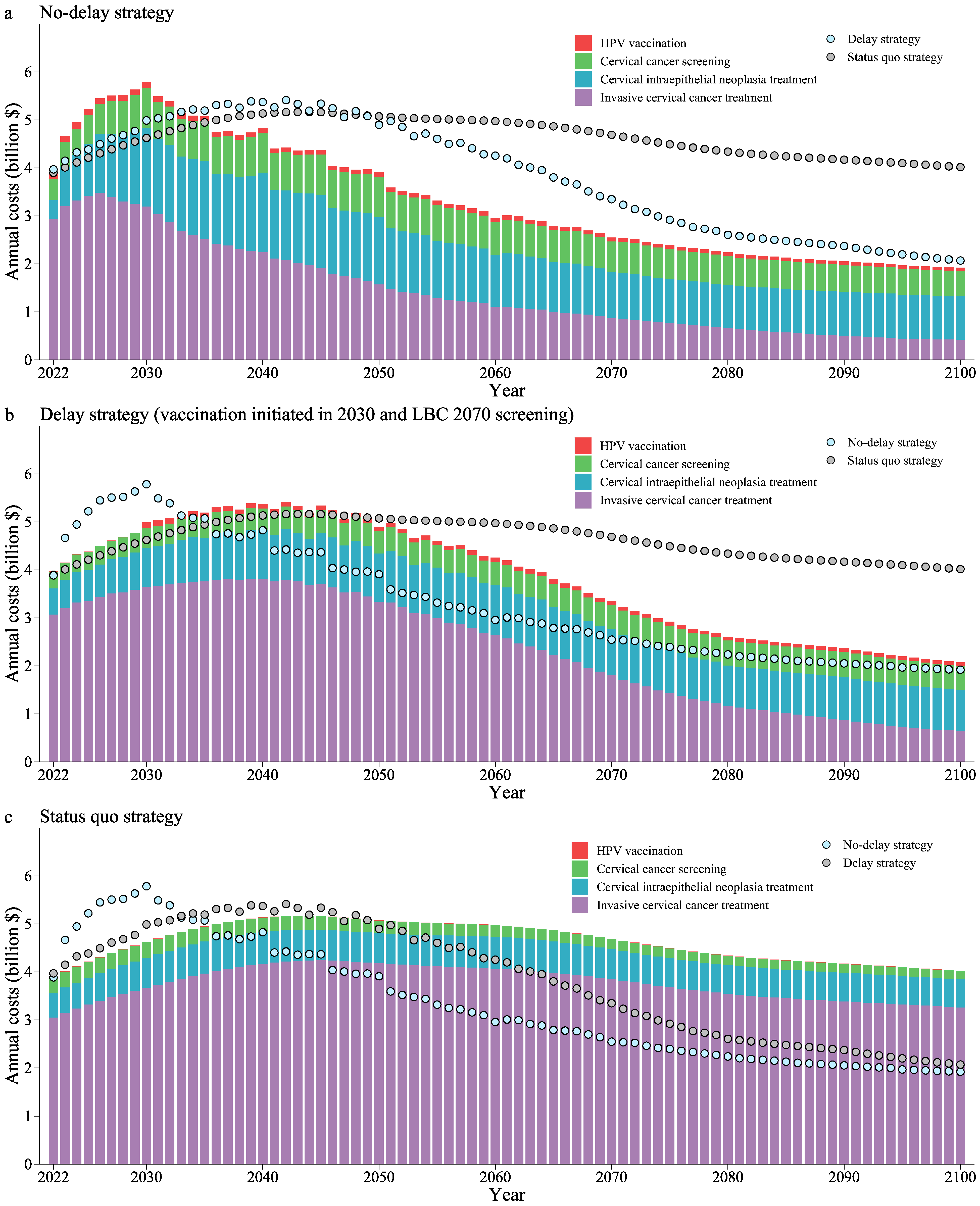


**Figure S5. Annual cost from each component of cervical cancer prevention and treatment for the domestic 2vHPV vaccine in the (a) no-delay scenario, (b) delay scenario, and (c) status quo scenario.**

The different colored bars indicate annual costs for each component, and the points indicate annual net costs of the other two alternative scenarios. The no-delay scenario was assumed to initiate large-scale domestic 2vHPV vaccination in 2022 with HPV 2030 screening. The delay scenario was assumed to initiate large-scale domestic 2vHPV vaccination in 2030 with LBC 2070 screening. The status quo scenario was assumed to maintain no vaccination and current LBC-based screening scenario in China. HPV 2030 screening represents switching to HPV-based screening at 5-year intervals in 2022, with linearly increasing age-specific uptake from status quo in 2021 to 70% in 2030, followed by a 1% increase every year till 90% is reached. LBC 2070 screening represents maintaining LBC-based screening at 3-year intervals, with linearly increasing age-specific uptake from status quo in 2021 to 70% in 2070, followed by a 1% increase every year till 90% is reached.


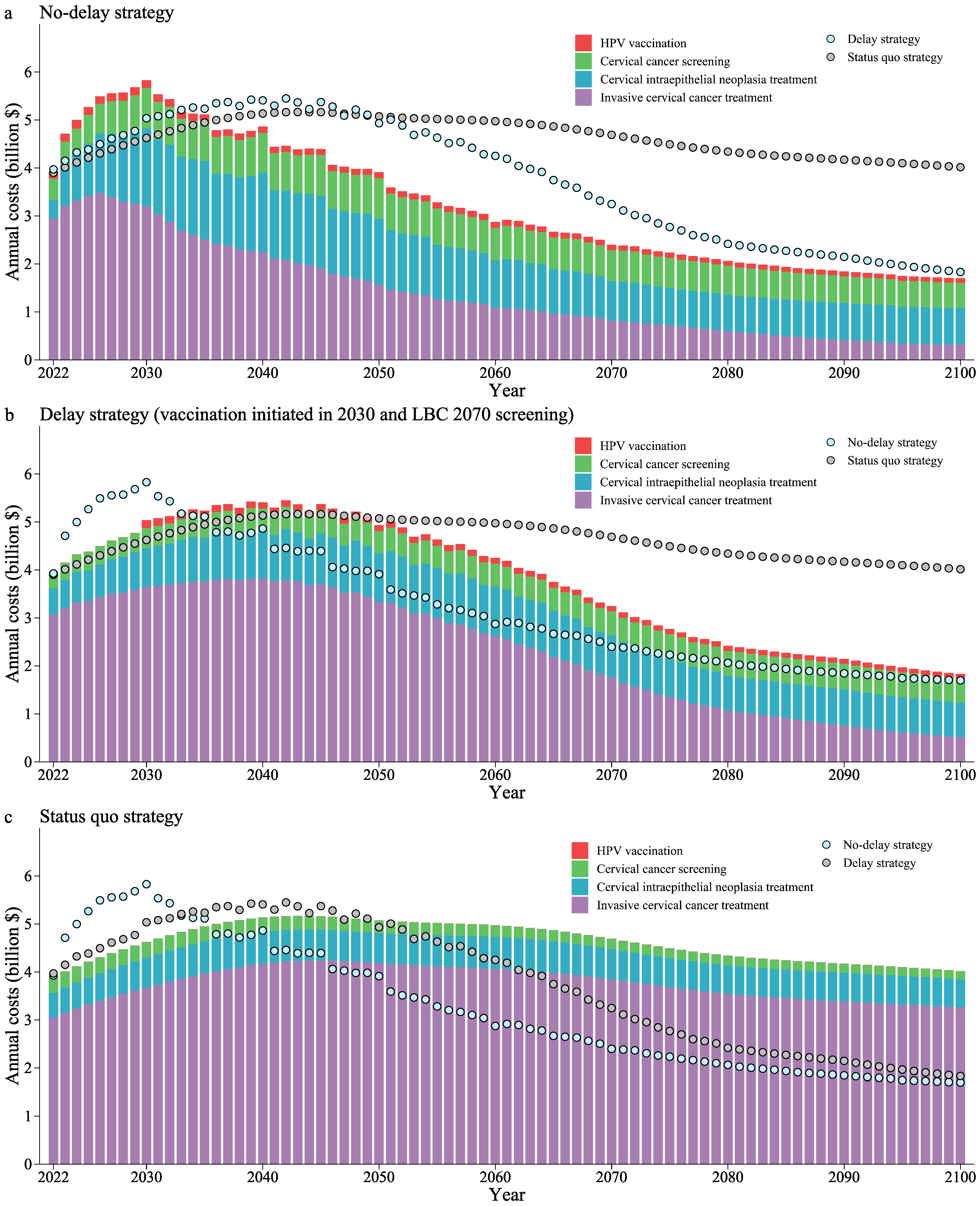


**Figure S6. Annual cost from each component of cervical cancer prevention and treatment for the imported 2vHPV vaccine in the (a) no-delay scenario, (b) delay scenario, and (c) status quo scenario.**

The different colored bars indicate annual costs for each component, and the points indicate annual net costs of the other two alternative scenarios. The no-delay scenario was assumed to initiate large-scale imported 2vHPV vaccination in 2022 with HPV 2030 screening. The delay scenario was assumed to initiate large-scale imported 2vHPV vaccination in 2030 with LBC 2070 screening. The status quo scenario was assumed to maintain no vaccination and current LBC-based screening scenario in China. HPV 2030 screening represents switching to HPV-based screening at 5-year intervals in 2022, with linearly increasing age-specific uptake from status quo in 2021 to 70% in 2030, followed by a 1% increase every year till 90% is reached. LBC 2070 screening represents maintaining LBC-based screening at 3-year intervals, with linearly increasing age-specific uptake from status quo in 2021 to 70% in 2070, followed by a 1% increase every year till 90% is reached.


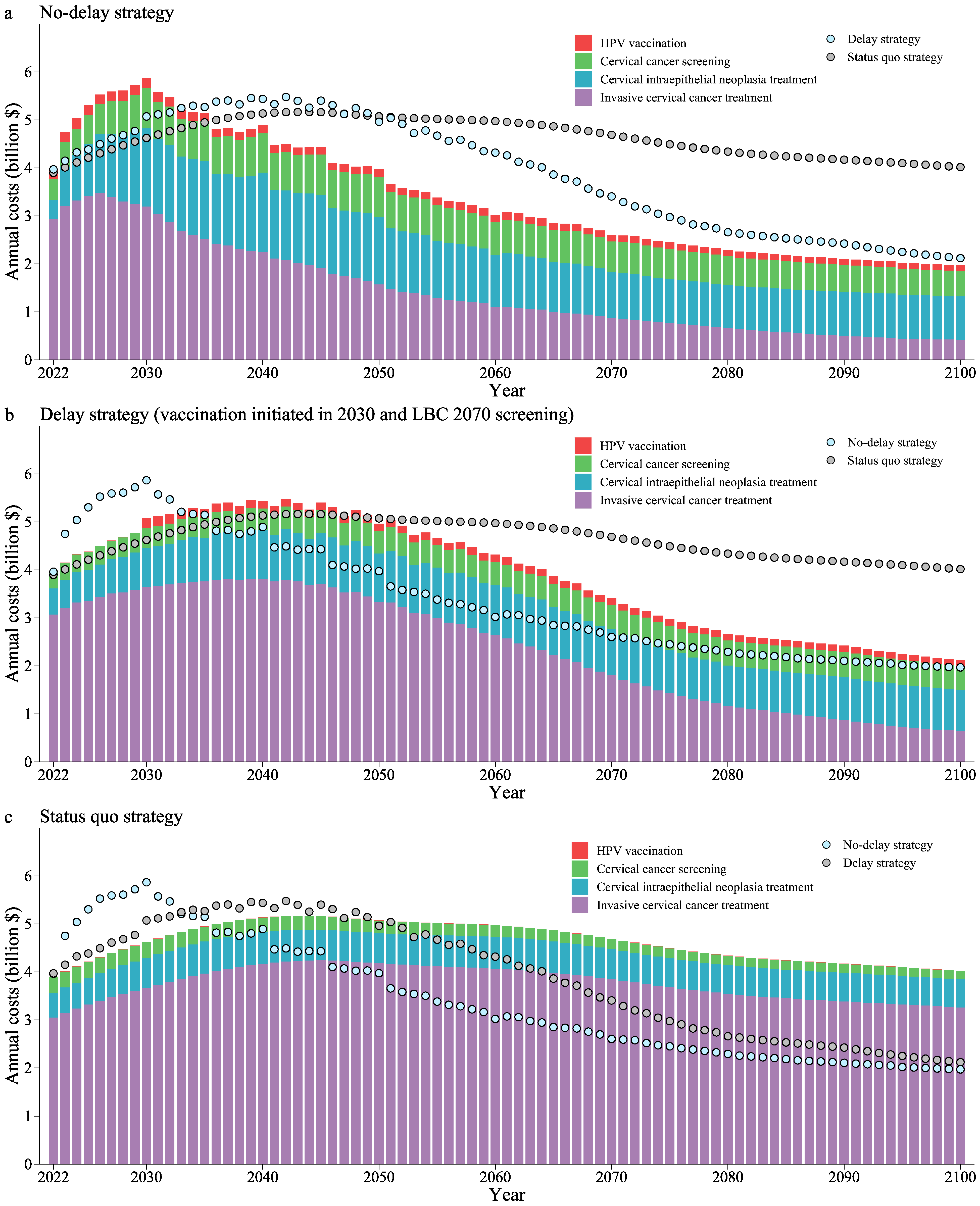


**Figure S7. Annual cost from each component of cervical cancer prevention and treatment for the 4vHPV vaccine in the (a) no-delay scenario, (b) delay scenario, and (c) status quo scenario.**

The different colored bars indicate annual costs for each component, and the points indicate annual net costs of the other two alternative scenarios. The no-delay scenario was assumed to initiate large-scale 4vHPV vaccination in 2022 with HPV 2030 screening. The delay scenario was assumed to initiate large-scale 4vHPV vaccination in 2030 with LBC 2070 screening. The status quo scenario was assumed to maintain no vaccination and current LBC-based screening scenario in China. HPV 2030 screening represents switching to HPV-based screening at 5-year intervals in 2022, with linearly increasing age-specific uptake from status quo in 2021 to 70% in 2030, followed by a 1% increase every year till 90% is reached. LBC 2070 screening represents maintaining LBC-based screening at 3-year intervals, with linearly increasing age-specific uptake from status quo in 2021 to 70% in 2070, followed by a 1% increase every year till 90% is reached.


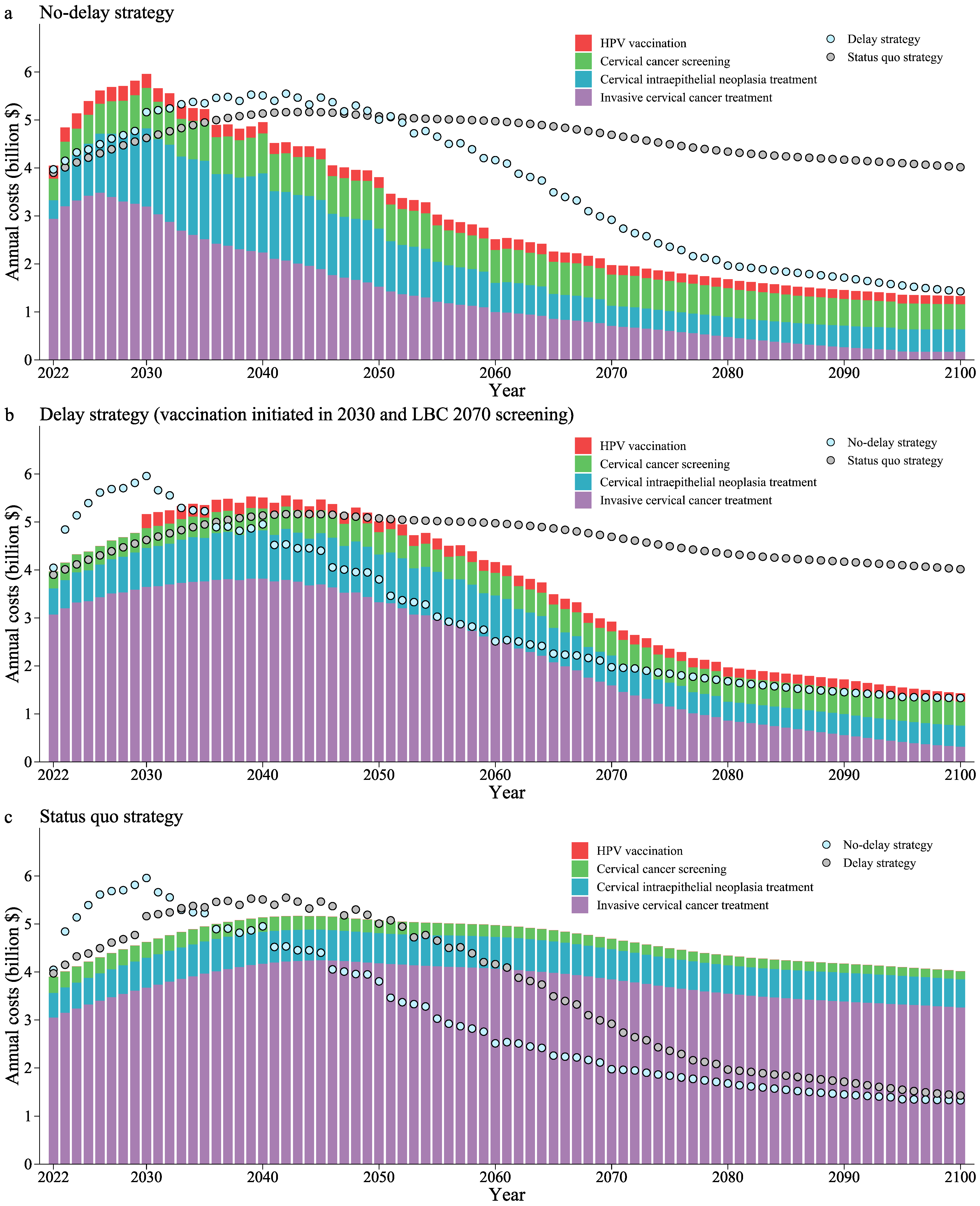


**Figure S8. Annual cost from each component of cervical cancer prevention and treatment for the 9vHPV vaccine in the (a) no-delay scenario, (b) delay scenario, and (c) status quo scenario.**

The different colored bars indicate annual costs for each component, and the points indicate annual net costs of the other two alternative scenarios. The no-delay scenario was assumed to initiate large-scale 9vHPV vaccination in 2022 with HPV 2030 screening. The delay scenario was assumed to initiate large-scale 9vHPV vaccination in 2030 with LBC 2070 screening. The status quo scenario was assumed to maintain no vaccination and current LBC-based screening scenario in China. HPV 2030 screening represents switching to HPV-based screening at 5-year intervals in 2022, with linearly increasing age-specific uptake from status quo in 2021 to 70% in 2030, followed by a 1% increase every year till 90% is reached. LBC 2070 screening represents maintaining LBC-based screening at 3-year intervals, with linearly increasing age-specific uptake from status quo in 2021 to 70% in 2070, followed by a 1% increase every year till 90% is reached.

**Table S10. Estimated elimination year (80% UI) of different large-scale vaccination and screening scenarios**

| Screening scenarios | No vaccination | Domestic 2vHPV | |  | Imported 2vHPV | |  | 4vHPV | |  | 9vHPV | |
| --- | --- | --- | --- | --- | --- | --- | --- | --- | --- | --- | --- | --- |
|  |  | Vaccination from 2022 | Vaccination from 2030 |  | Vaccination from 2022 | Vaccination from 2030 |  | Vaccination from 2022 | Vaccination from 2030 |  | Vaccination from 2022 | Vaccination from 2030 |
| HPV 2030 | 9.20* | 2063 (2060-2070) | 2073 (2068-2078) |  | 2060 (2059-2065) | 2070 (2068-2074) |  | 2063 (2060-2070) | 2073 (2068-2078) |  | 2059 (2056-2060) | 2068 (2064-2069) |
| LBC 2030 | 12.75* | 4.09* | 4.16* |  | 2072 (2069-2074) | 2080 (2077-2084) |  | 4.09* | 4.16* |  | 2063 (2062-2065) | 2071 (2071-2073) |
| HPV 2050 | 9.20* | 2070 (2066-2073) | 2074 (2072-2080) |  | 2067 (2065-2070) | 2073 (2070-2076) |  | 2070 (2066-2073) | 2074 (2072-2080) |  | 2063 (2061-2065) | 2069 (2068-2072) |
| LBC 2050 | 12.75* | 4.09* | 4.16* |  | 2073 (2072-2076) | 2080 (2078-2085) |  | 4.09* | 4.16* |  | 2066 (2065-2067) | 2073 (2072-2074) |
| HPV 2070 | 9.24* | 2079 (2076-2085) | 2085 (2081-2090) |  | 2074 (2072-2078) | 2080 (2078-2083) |  | 2079 (2076-2085) | 2085 (2081-2090) |  | 2068 (2066-2070) | 2073 (2073-2076) |
| LBC 2070 | 12.83* | 4.13* | 4.21* |  | 2082 (2079-2085) | 2087 (2085-2091) |  | 4.13* | 4.21* |  | 2070 (2069-2071) | 2077 (2076-2078) |
| Status quo | 23.52* | 9.11* | 9.23* |  | 6.97* | 7.12* |  | 9.11* | 9.23* |  | 2079 (2078-2080) | 2090 (2088-2091) |

*Cervical cancer will not be eliminated in this scenario and the values represent cervical cancer incidence (per 100,000 women) in 2100.

Seven screening scenarios with different modalities and rates of increase in coverage for target population of women aged 35-64 years. “HPV 2030”, “HPV 2050”, and “HPV 2070” screening scenarios represent switching to HPV-based screening at 5-year intervals in 2022, with linearly increasing age-specific uptake from status quo in 2021, to 70% in 2030 (rapid), 2050 (moderate), and 2070 (gradual), respectively, followed by a 1% increase every year till 90% is reached. “LBC 2030”, “LBC 2050”, and “LBC 2070” screening scenarios represent maintaining LBC-based screening at 3-year intervals, with the corresponding rapid, moderate, and gradual increase in age-specific uptake. Status quo represents maintaining LBC-based screening with current coverage.

Abbreviations: HPV, human papillomavirus; LBC, liquid-based cytology; UI, uncertainty interval.

## **2.2 Sensitivity analysis**

The effect of varied vaccination costs on the cost-effectiveness analysis is shown in Figure S9-S12. Deterministic sensitivity analyses are displayed in a tornado diagram for model parameters (Figure S13-S16). In this diagram, each bar represents the impact of uncertainty in an individual variable. Probabilistic sensitivity analyses are displayed in scatter plots for discounted incremental costs and QALYs (Figure S17).


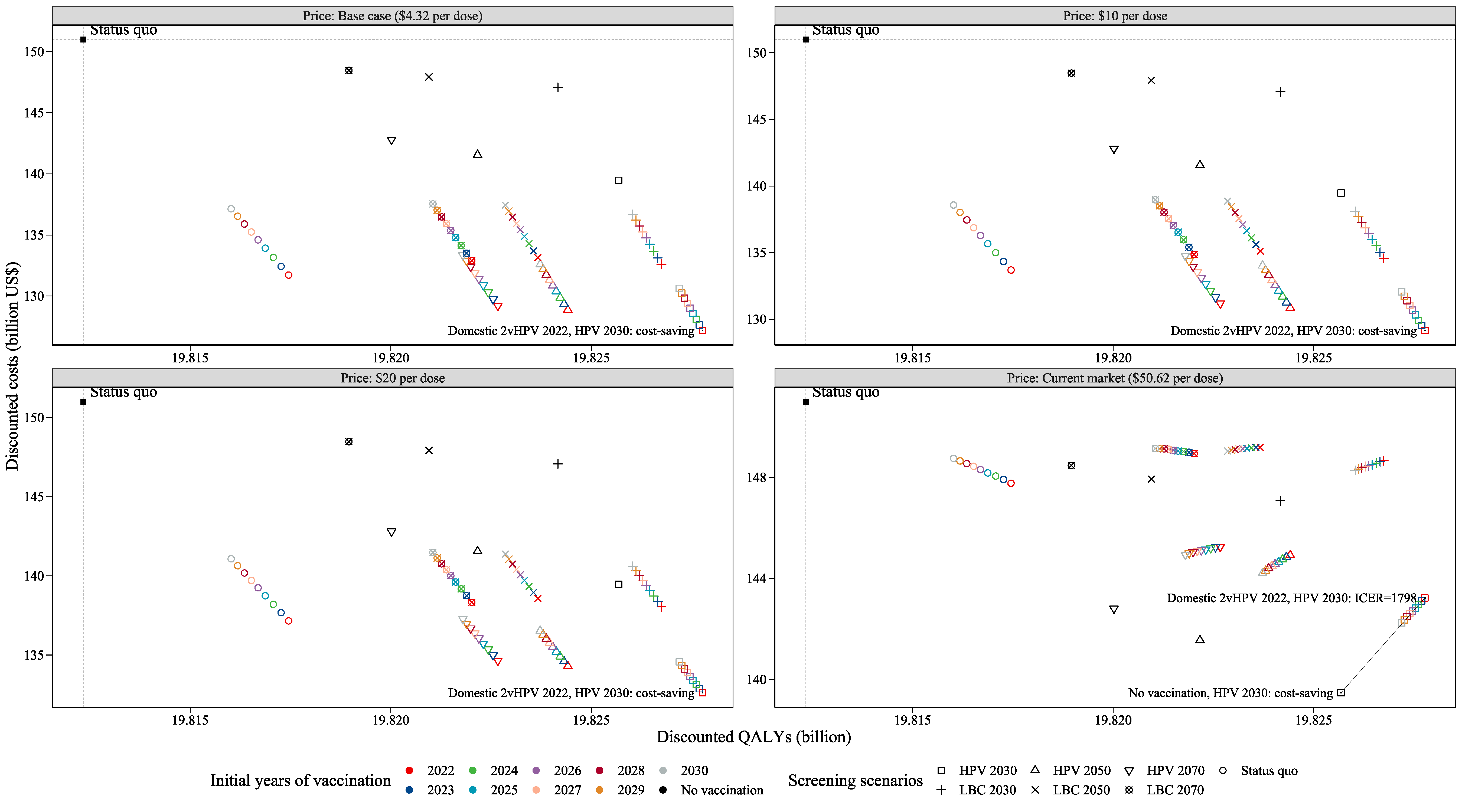


**Figure S9. Cost-effectiveness plane for domestic 2vHPV vaccination and screening scenarios at different vaccine prices**

The black squares indicate the reference scenario, which is the status quo scenario. The labels represent the vaccination initiation years and the screening scenario of scenarios located on the cost-effectiveness frontier and their ICERs compared with the next most costly non-dominated scenario. The scenarios on the upper left of the frontier are dominated by the scenarios on the lower right of them. When the scenarios are cost-saving at the vaccine price, the ICERs are negative and not shown. Different shaped points indicate seven screening scenarios with different modalities and rates of increase in coverage for target population of women aged 35-64 years. “HPV 2030”, “HPV 2050”, and “HPV 2070” screening scenarios represent switching to HPV-based screening at 5-year intervals in 2022, with linearly increasing age-specific uptake from status quo in 2021, to 70% in 2030 (rapid), 2050 (moderate), and 2070 (gradual), respectively, followed by a 1% increase every year till 90% is reached. “LBC 2030”, “LBC 2050”, and “LBC 2070” screening scenarios represent maintaining LBC-based screening at 3-year intervals, with the corresponding rapid, moderate, and gradual increase in age-specific uptake. Status quo represents maintaining LBC-based screening with current coverage.

Abbreviations: HPV, human papillomavirus; LBC, liquid-based cytology; QALY, quality-adjusted life-year; ICER, incremental cost-effectiveness ratio.

**Figure S10. Cost-effectiveness frontier for imported 2vHPV vaccination and screening scenarios at different vaccine prices**


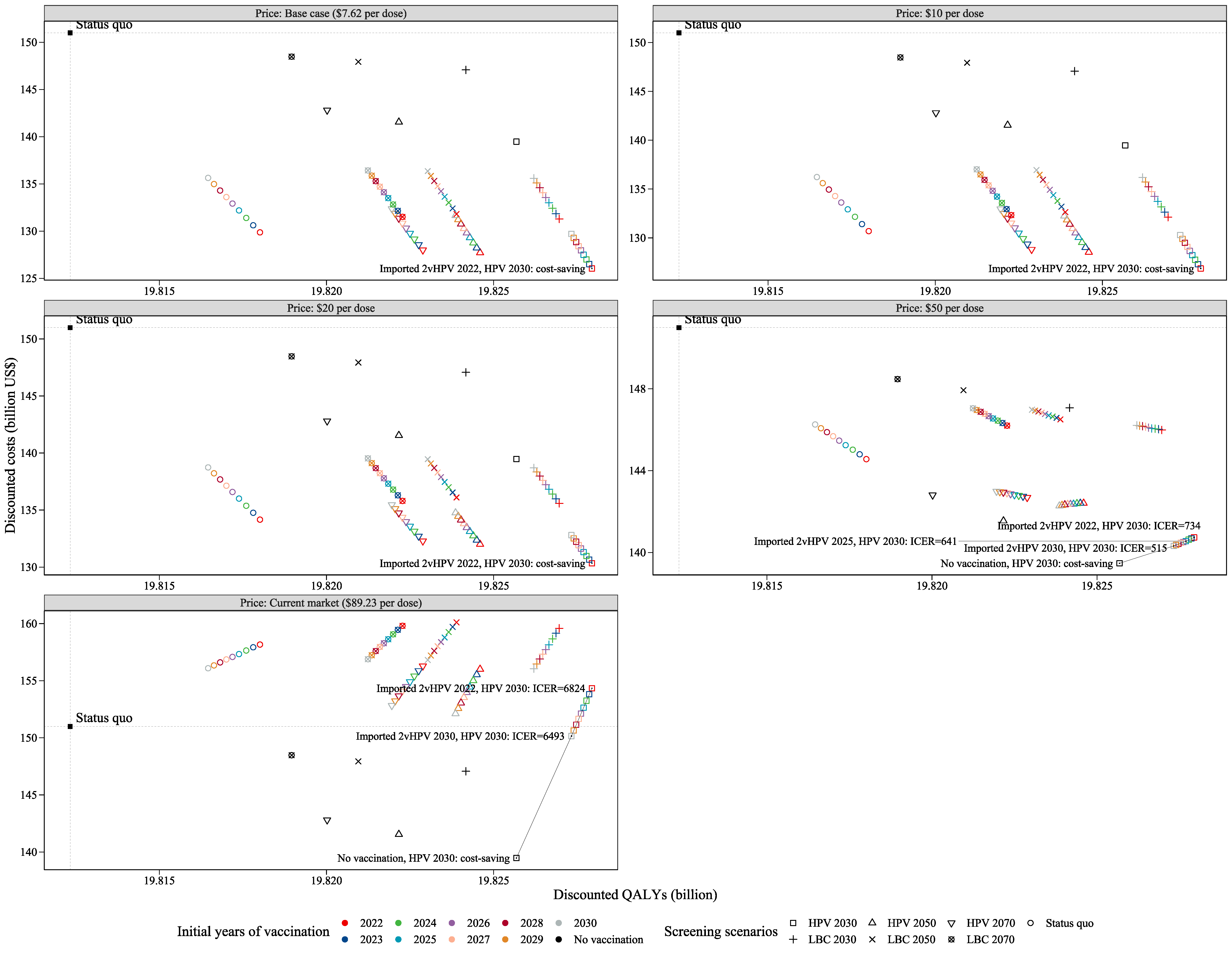


The black squares indicate the reference scenario, which is the status quo scenario. The labels represent the vaccination initiation years and the screening scenario of scenarios located on the cost-effectiveness frontier and their ICERs compared with the next most costly non-dominated scenario. The scenarios on the upper left of the frontier are dominated by the scenarios on the lower right of them. When the scenarios are cost-saving at the vaccine price, the ICERs are negative and not shown. Different shaped points indicate seven screening scenarios with different modalities and rates of increase in coverage for target population of women aged 35-64 years. “HPV 2030”, “HPV 2050”, and “HPV 2070” screening scenarios represent switching to HPV-based screening at 5-year intervals in 2022, with linearly increasing age-specific uptake from status quo in 2021, to 70% in 2030 (rapid), 2050 (moderate), and 2070 (gradual), respectively, followed by a 1% increase every year till 90% is reached. “LBC 2030”, “LBC 2050”, and “LBC 2070” screening scenarios represent maintaining LBC-based screening at 3-year intervals, with the corresponding rapid, moderate, and gradual increase in age-specific uptake. Status quo represents maintaining LBC-based screening with current coverage.

Abbreviations: HPV, human papillomavirus; LBC, liquid-based cytology; QALY, quality-adjusted life-year; ICER, incremental cost-effectiveness ratio.


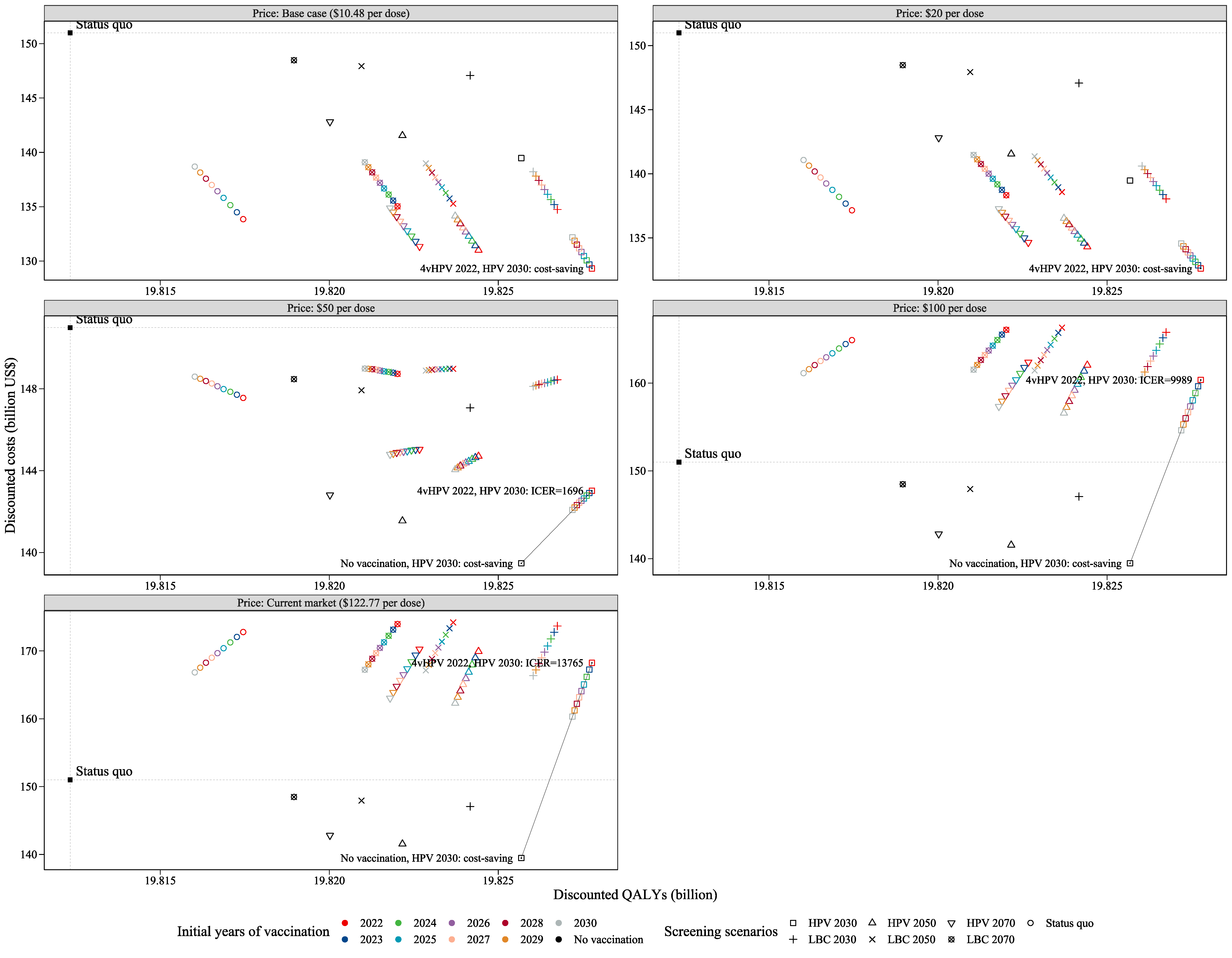


**Figure S11. Cost-effectiveness frontier for 4vHPV vaccination and screening scenarios at different vaccine prices**

The black squares indicate the reference scenario, which is the status quo scenario. The labels represent the vaccination initiation years and the screening scenario of scenarios located on the cost-effectiveness frontier and their ICERs compared with the next most costly non-dominated scenario. The scenarios on the upper left of the frontier are dominated by the scenarios on the lower right of them. When the scenarios are cost-saving at the vaccine price, the ICERs are negative and not shown. Different shaped points indicate seven screening scenarios with different modalities and rates of increase in coverage for target population of women aged 35-64 years. “HPV 2030”, “HPV 2050”, and “HPV 2070” screening scenarios represent switching to HPV-based screening at 5-year intervals in 2022, with linearly increasing age-specific uptake from status quo in 2021, to 70% in 2030 (rapid), 2050 (moderate), and 2070 (gradual), respectively, followed by a 1% increase every year till 90% is reached. “LBC 2030”, “LBC 2050”, and “LBC 2070” screening scenarios represent maintaining LBC-based screening at 3-year intervals, with the corresponding rapid, moderate, and gradual increase in age-specific uptake. Status quo represents maintaining LBC-based screening with current coverage.

Abbreviations: HPV, human papillomavirus; LBC, liquid-based cytology; QALY, quality-adjusted life-year; ICER, incremental cost-effectiveness ratio.

**Figure S12. Cost-effectiveness frontier for 9vHPV vaccination and screening scenarios at different vaccine prices**


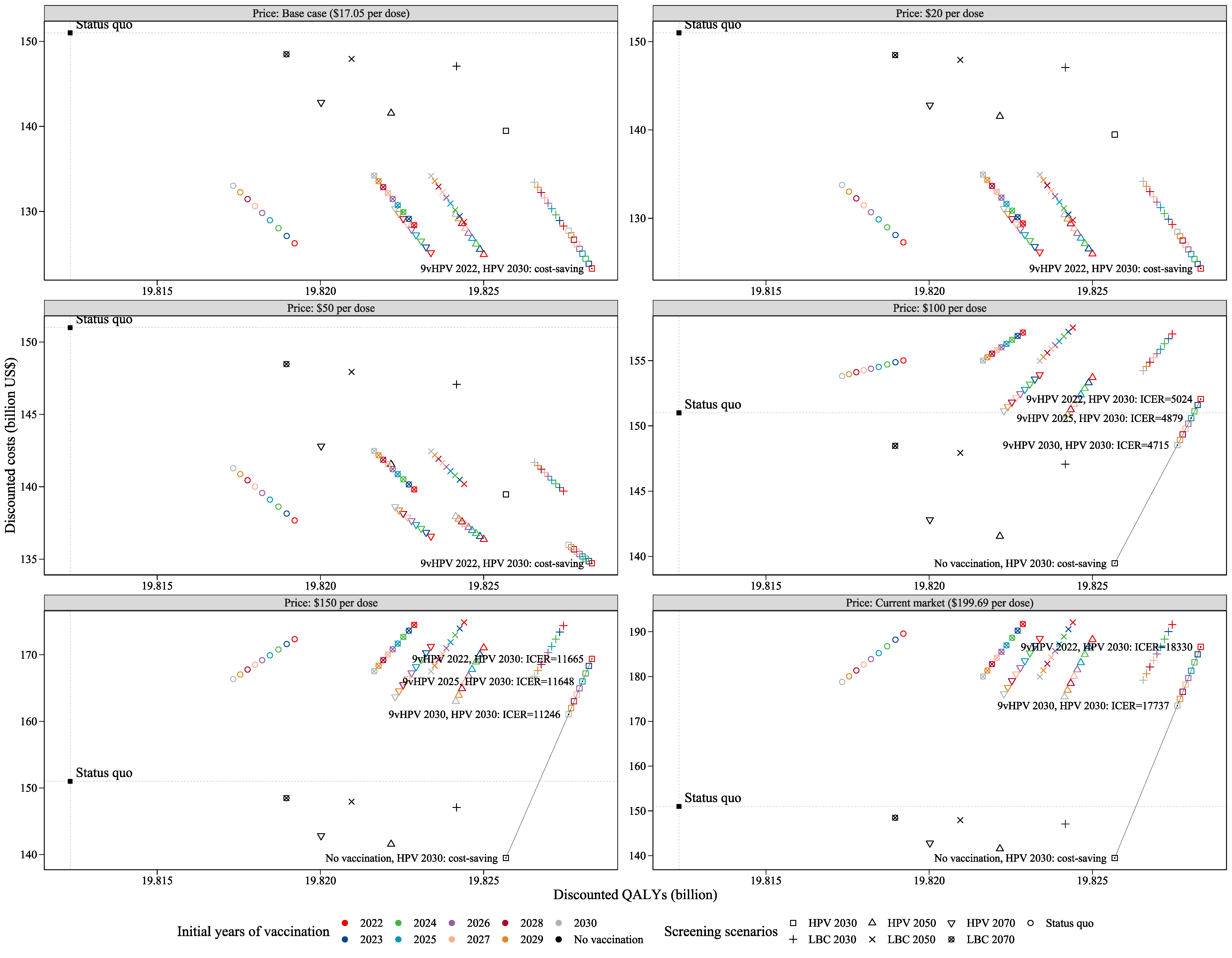


The black squares indicate the reference scenario, which is the status quo scenario. The labels represent the vaccination initiation years and the screening scenario of scenarios located on the cost-effectiveness frontier and their ICERs compared with the next most costly non-dominated scenario. The scenarios on the upper left of the frontier are dominated by the scenarios on the lower right of them. When the scenarios are cost-saving at the vaccine price, the ICERs are negative and not shown. Different shaped points indicate seven screening scenarios with different modalities and rates of increase in coverage for target population of women aged 35-64 years. “HPV 2030”, “HPV 2050”, and “HPV 2070” screening scenarios represent switching to HPV-based screening at 5-year intervals in 2022, with linearly increasing age-specific uptake from status quo in 2021, to 70% in 2030 (rapid), 2050 (moderate), and 2070 (gradual), respectively, followed by a 1% increase every year till 90% is reached. “LBC 2030”, “LBC 2050”, and “LBC 2070” screening scenarios represent maintaining LBC-based screening at 3-year intervals, with the corresponding rapid, moderate, and gradual increase in age-specific uptake. Status quo represents maintaining LBC-based screening with current coverage.

Abbreviations: HPV, human papillomavirus; LBC, liquid-based cytology; QALY, quality-adjusted life-year; ICER, incremental cost-effectiveness ratio.


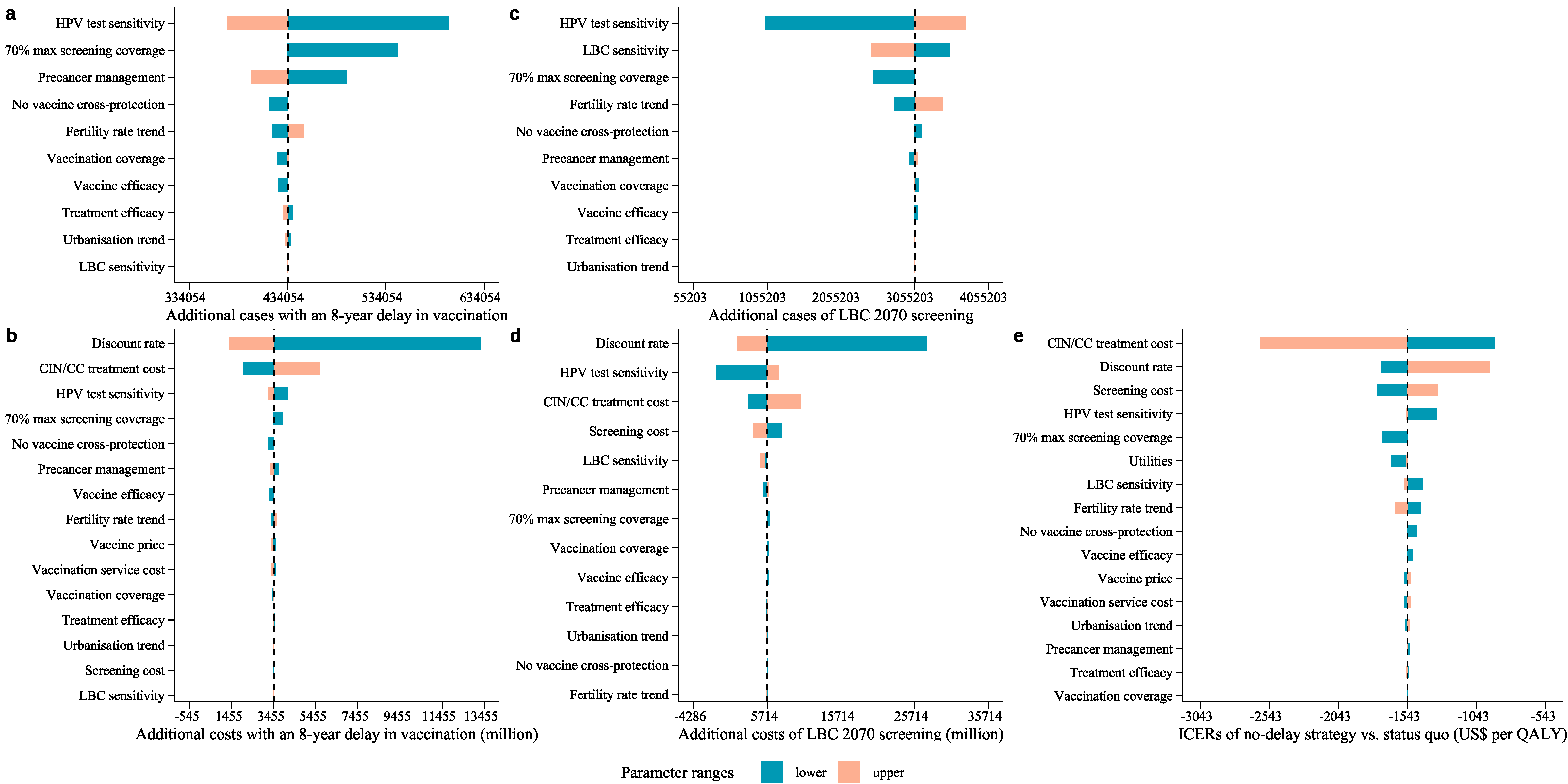


**Figure S13. Deterministic sensitivity analysis for the health and economic impact of delay in domestic 2vHPV vaccination and large-scale screening.**

The health and economic impact is shown as the additional numbers of (a) cases and (b) costs of the scenario with 8-year delayed domestic 2vHPV vaccination and HPV 2030 screening (vaccination delay) compared with no-delay scenario, (c) cases and (d) costs of LBC 2070 screening scenario with immediate vaccination (screening delay) compared with no-delay scenario, and (e) the ICERs of no-delay scenario compared with the status quo scenario. The no-delay scenario was assumed to initiate large-scale vaccination in 2022 with HPV 2030 screening. The status quo scenario was assumed to maintain no vaccination and current LBC-based screening scenario in China. HPV 2030 screening represents switching to HPV-based screening at 5-year intervals in 2022, with linearly increasing age-specific uptake from status quo in 2021 to 70% in 2030, followed by a 1% increase every year till 90% is reached. LBC 2070 screening represents maintaining LBC-based screening at 3-year intervals, with linearly increasing age-specific uptake from status quo in 2021 to 70% in 2070, followed by a 1% increase every year till 90% is reached.

Abbreviations: HPV, Human Papillomavirus; LBC, liquid-based cytology; CIN, cervical intraepithelial neoplasia; CC, cervical cancer; ICER, incremental cost-effectiveness ratio; QALY, quality-adjusted life-year.


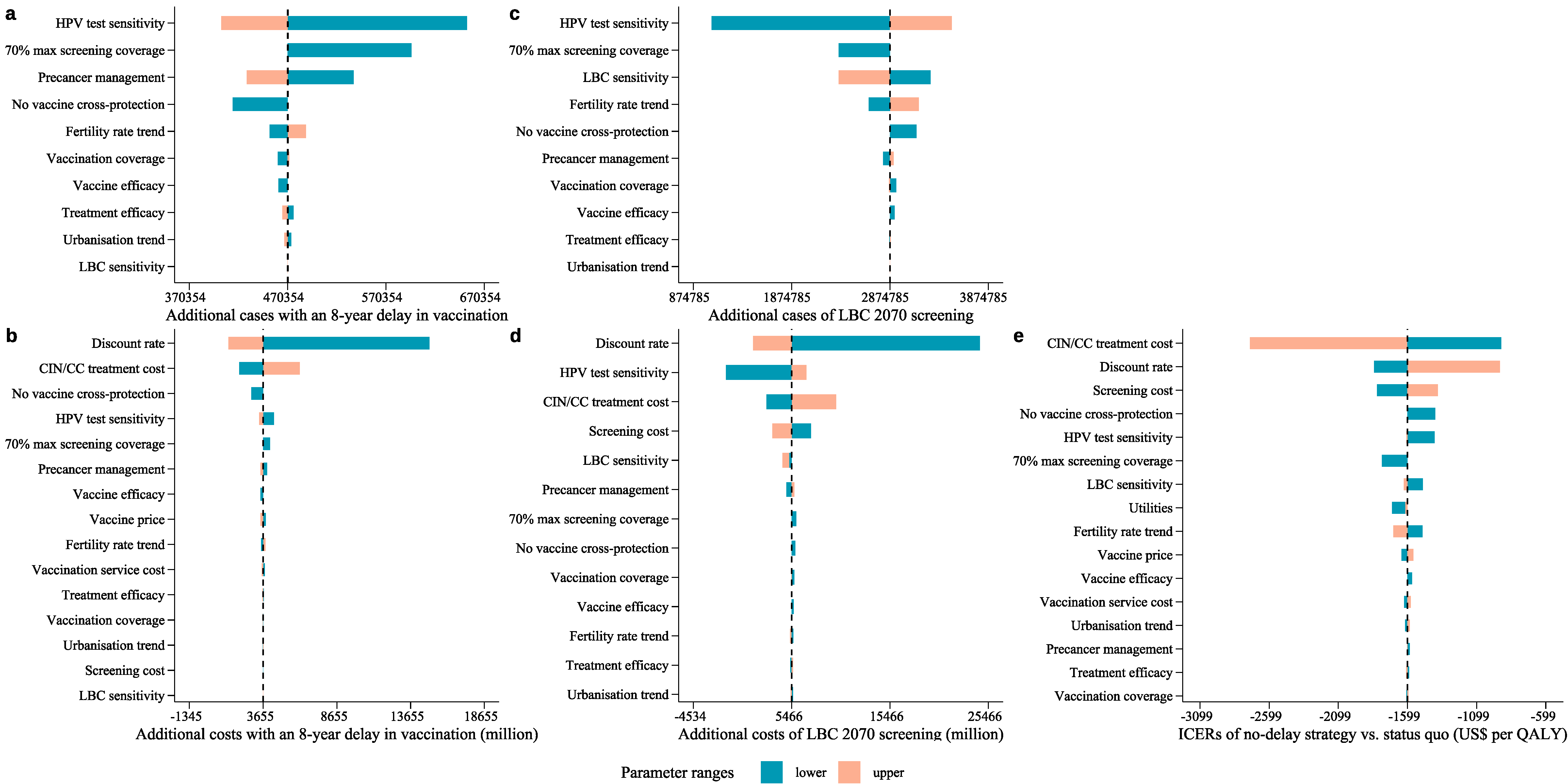


**Figure S14. Deterministic sensitivity analysis for the health and economic impact of delay in imported 2vHPV vaccination and large-scale screening.**

The health and economic impact is shown as the additional numbers of (a) cases and (b) costs of the scenario with 8-year delayed imported 2vHPV vaccination and HPV 2030 screening (vaccination delay) compared with no-delay scenario, (c) cases and (d) costs of LBC 2070 screening scenario with immediate vaccination (screening delay) compared with no-delay scenario, and (e) the ICERs of no-delay scenario compared with the status quo scenario. The no-delay scenario was assumed to initiate large-scale vaccination in 2022 with HPV 2030 screening. The status quo scenario was assumed to maintain no vaccination and current LBC-based screening scenario in China. HPV 2030 screening represents switching to HPV-based screening at 5-year intervals in 2022, with linearly increasing age-specific uptake from status quo in 2021 to 70% in 2030, followed by a 1% increase every year till 90% is reached. LBC 2070 screening represents maintaining LBC-based screening at 3-year intervals, with linearly increasing age-specific uptake from status quo in 2021 to 70% in 2070, followed by a 1% increase every year till 90% is reached.

Abbreviations: HPV, Human Papillomavirus; LBC, liquid-based cytology; CIN, cervical intraepithelial neoplasia; CC, cervical cancer; ICER, incremental cost-effectiveness ratio; QALY, quality-adjusted life-year.


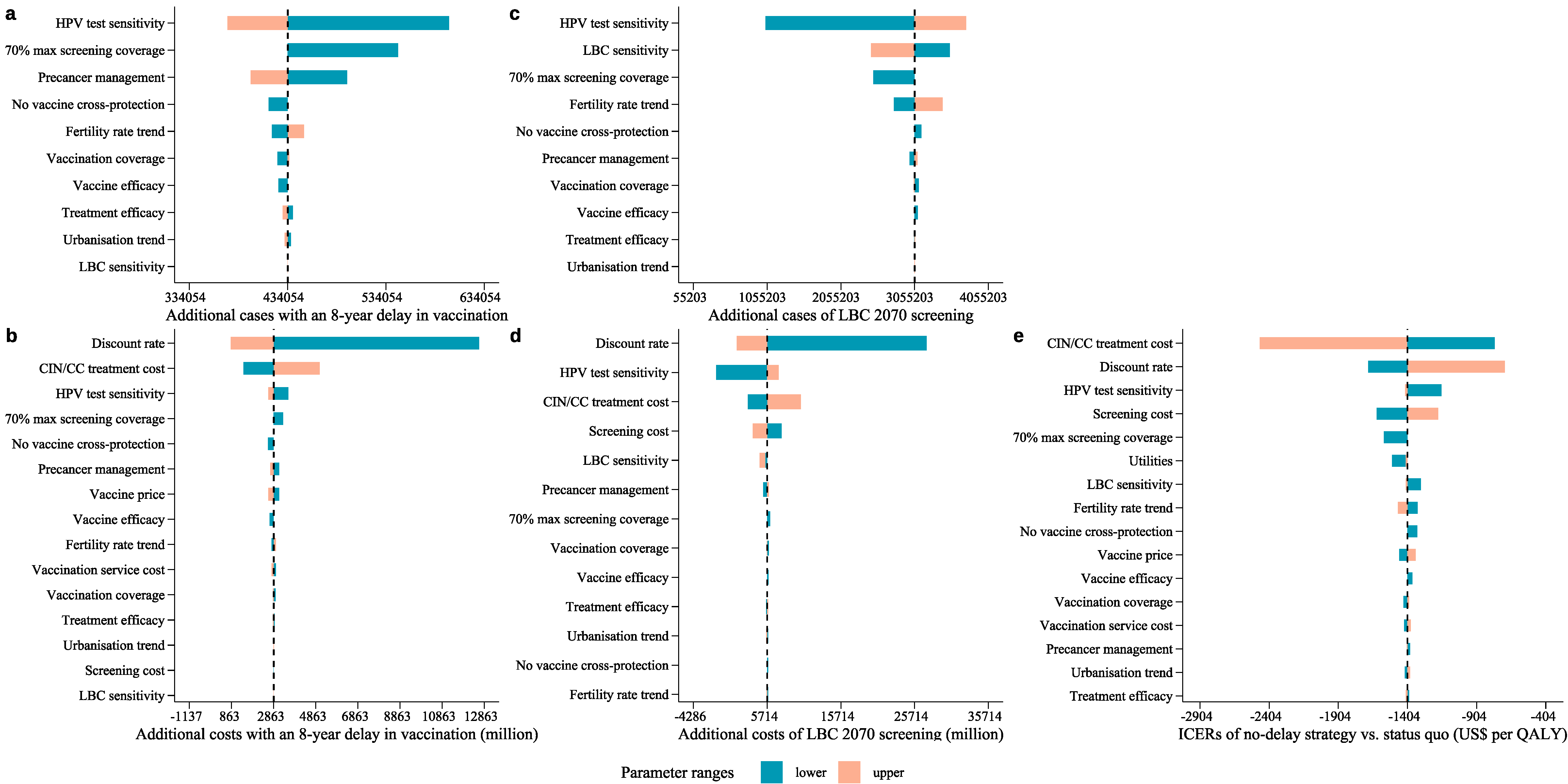


**Figure S15. Deterministic sensitivity analysis for the health and economic impact of delay in 4vHPV vaccination and large-scale screening.**

The health and economic impact is shown as the additional numbers of (a) cases and (b) costs of the scenario with 8-year delayed 4vHPV vaccination and HPV 2030 screening (vaccination delay) compared with no-delay scenario, (c) cases and (d) costs of LBC 2070 screening scenario with immediate vaccination (screening delay) compared with no-delay scenario, and (e) the ICERs of no-delay scenario compared with the status quo scenario. The no-delay scenario was assumed to initiate large-scale vaccination in 2022 with HPV 2030 screening. The status quo scenario was assumed to maintain no vaccination and current LBC-based screening scenario in China. HPV 2030 screening represents switching to HPV-based screening at 5-year intervals in 2022, with linearly increasing age-specific uptake from status quo in 2021 to 70% in 2030, followed by a 1% increase every year till 90% is reached. LBC 2070 screening represents maintaining LBC-based screening at 3-year intervals, with linearly increasing age-specific uptake from status quo in 2021 to 70% in 2070, followed by a 1% increase every year till 90% is reached.

Abbreviations: HPV, Human Papillomavirus; LBC, liquid-based cytology; CIN, cervical intraepithelial neoplasia; CC, cervical cancer; ICER, incremental cost-effectiveness ratio; QALY, quality-adjusted life-year.


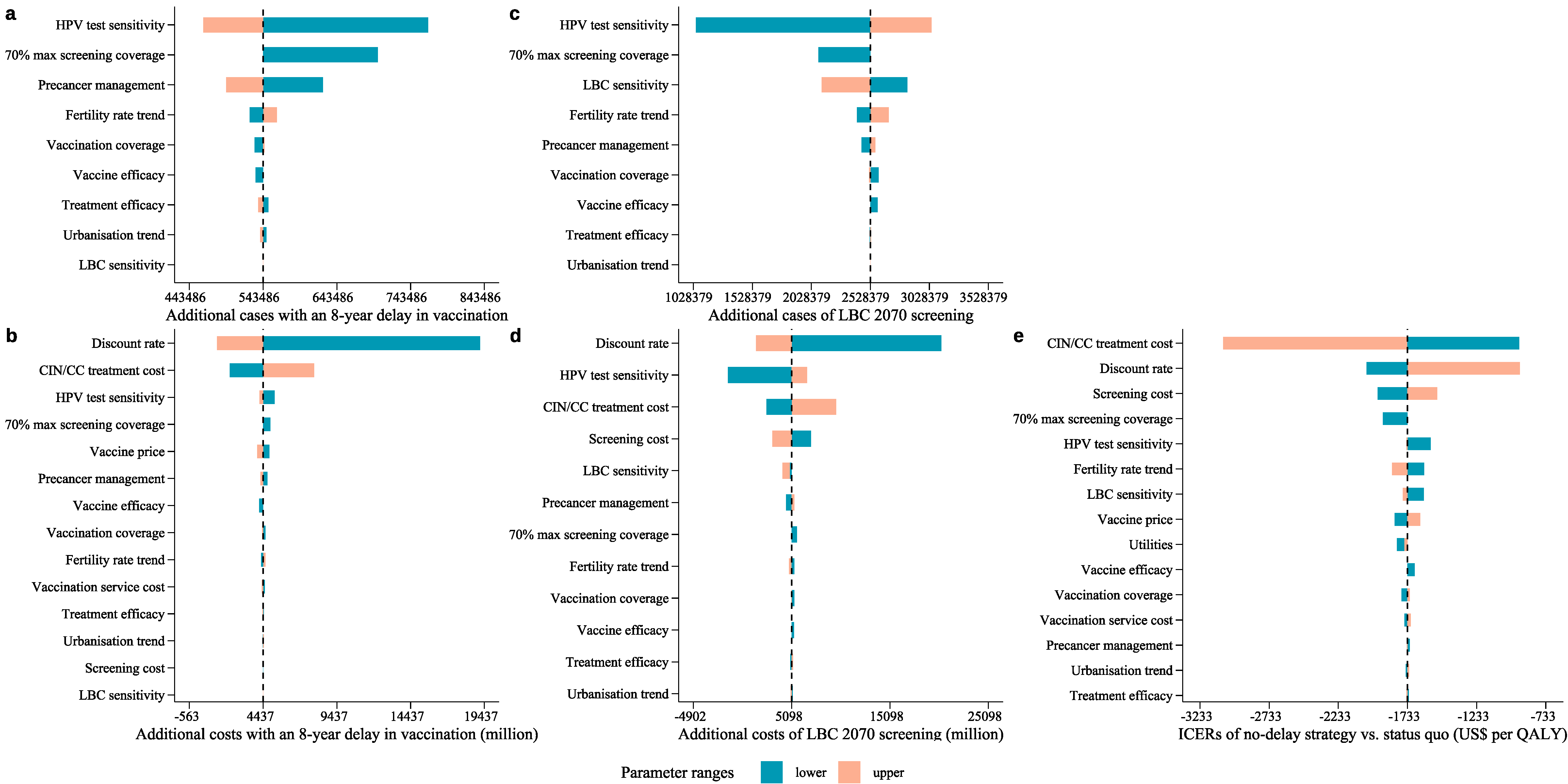


**Figure S16. Deterministic sensitivity analysis for the health and economic impact of delay in 9vHPV vaccination and large-scale screening.**

The health and economic impact is shown as the additional numbers of (a) cases and (b) costs of the scenario with 8-year delayed 9vHPV vaccination and HPV 2030 screening (vaccination delay) compared with no-delay scenario, (c) cases and (d) costs of LBC 2070 screening scenario with immediate vaccination (screening delay) compared with no-delay scenario, and (e) the ICERs of no-delay scenario compared with the status quo scenario. The no-delay scenario was assumed to initiate large-scale vaccination in 2022 with HPV 2030 screening. The status quo scenario was assumed to maintain no vaccination and current LBC-based screening scenario in China. HPV 2030 screening represents switching to HPV-based screening at 5-year intervals in 2022, with linearly increasing age-specific uptake from status quo in 2021 to 70% in 2030, followed by a 1% increase every year till 90% is reached. LBC 2070 screening represents maintaining LBC-based screening at 3-year intervals, with linearly increasing age-specific uptake from status quo in 2021 to 70% in 2070, followed by a 1% increase every year till 90% is reached.

Abbreviations: HPV, Human Papillomavirus; LBC, liquid-based cytology; CIN, cervical intraepithelial neoplasia; CC, cervical cancer; ICER, incremental cost-effectiveness ratio; QALY, quality-adjusted life-year.


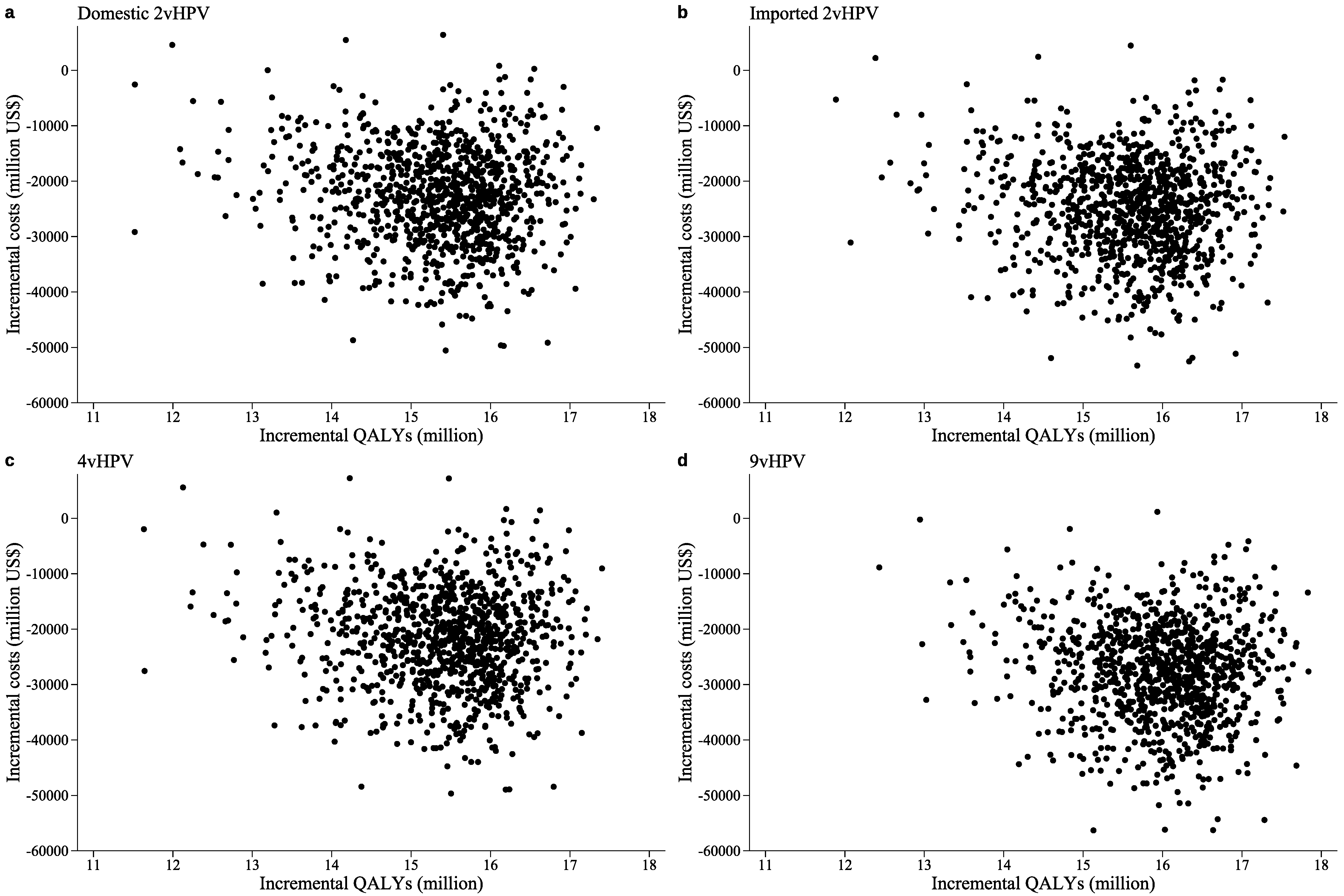


**Figure S17. Probabilistic sensitivity analyses for no-delay vs. status quo scenario (3% discount).** (a) Domestic 2vHPV; (b) imported 2vHPV; (c) 4vHPV; and (d) 9vHPV vaccines.

Abbreviations: HPV, Human Papillomavirus; QALY, quality-adjusted life-year.

Reference:

1. Xia C, Hu S, Xu X, et al. Projections up to 2100 and a budget optimisation strategy towards cervical cancer elimination in China: a modelling study. *The Lancet Public health* 2019; **4**(9): e462-e72.

2. Xia C, Xu X, Zhao X, et al. Effectiveness and cost-effectiveness of eliminating cervical cancer through a tailored optimal pathway: a modeling study. *BMC medicine* 2021; **19**(1): 62.

3. Pan American Health Organization. PAHO Revolving Fund Vaccine Prices for 2022. <https://www.paho.org/en/documents/paho-revolving-fund-vaccine-prices-2022> (accessed June 1, 2022).

4. World Bank. World Bank Country and Lending Groups. 2022. <https://datahelpdesk.worldbank.org/knowledgebase/articles/906519-world-bank-country-and-lending-groups> (accessed June 1, 2022).

5. Canfell K, Barnabas R, Patnick J, Beral V. The predicted effect of changes in cervical screening practice in the UK: results from a modelling study. *British journal of cancer* 2004; **91**(3): 530-6.

6. Goldie SJ, Grima D, Kohli M, Wright TC, Weinstein M, Franco E. A comprehensive natural history model of HPV infection and cervical cancer to estimate the clinical impact of a prophylactic HPV-16/18 vaccine. *International journal of cancer* 2003; **106**(6): 896-904.

7. Haeussler K, den Hout AV, Baio G. A dynamic Bayesian Markov model for health economic evaluations of interventions in infectious disease. *BMC medical research methodology* 2018; **18**(1): 82.

8. Liu YJ, Zhang Q, Hu SY, Zhao FH. Effect of vaccination age on cost-effectiveness of human papillomavirus vaccination against cervical cancer in China. *BMC cancer* 2016; **16**: 164.

9. Haeussler K, Marcellusi A, Mennini FS, et al. Cost-Effectiveness Analysis of Universal Human Papillomavirus Vaccination Using a Dynamic Bayesian Methodology: The BEST II Study. *Value in health : the journal of the International Society for Pharmacoeconomics and Outcomes Research* 2015; **18**(8): 956-68.

10. Myers ER, McCrory DC, Nanda K, Bastian L, Matchar DB. Mathematical model for the natural history of human papillomavirus infection and cervical carcinogenesis. *American journal of epidemiology* 2000; **151**(12): 1158-71.

11. Yokoyama M, Iwasaka T, Nagata C, et al. Prognostic factors associated with the clinical outcome of cervical intraepithelial neoplasia: a cohort study in Japan. *Cancer letters* 2003; **192**(2): 171-9.

12. Sawaya GF, Sanstead E, Alarid-Escudero F, et al. Estimated Quality of Life and Economic Outcomes Associated With 12 Cervical Cancer Screening Strategies: A Cost-effectiveness Analysis. *JAMA internal medicine* 2019; **179**(7): 867-78.

13. Johnson HC, Elfström KM, Edmunds WJ. Inference of type-specific HPV transmissibility, progression and clearance rates: a mathematical modelling approach. *PloS one* 2012; **7**(11): e49614.

14. Abbas KM, van Zandvoort K, Brisson M, Jit M. Effects of updated demography, disability weights, and cervical cancer burden on estimates of human papillomavirus vaccination impact at the global, regional, and national levels: a PRIME modelling study. *The Lancet Global health* 2020; **8**(4): e536-e44.

15. Brisson M, Kim JJ, Canfell K, et al. Impact of HPV vaccination and cervical screening on cervical cancer elimination: a comparative modelling analysis in 78 low-income and lower-middle-income countries. *Lancet (London, England)* 2020; **395**(10224): 575-90.

16. Canfell K, Kim JJ, Brisson M, et al. Mortality impact of achieving WHO cervical cancer elimination targets: a comparative modelling analysis in 78 low-income and lower-middle-income countries. *Lancet (London, England)* 2020; **395**(10224): 591-603.

17. Malagón T, Drolet M, Boily MC, et al. Cross-protective efficacy of two human papillomavirus vaccines: a systematic review and meta-analysis. *The Lancet Infectious diseases* 2012; **12**(10): 781-9.

18. Cuzick J, Clavel C, Petry KU, et al. Overview of the European and North American studies on HPV testing in primary cervical cancer screening. *International journal of cancer* 2006; **119**(5): 1095-101.

19. Yu W, Lu M, Wang H, et al. Routine immunization services costs and financing in China, 2015. *Vaccine* 2018; **36**(21): 3041-7.

20. Van Rosmalen J, de Kok IM, van Ballegooijen M. Cost-effectiveness of cervical cancer screening: cytology versus human papillomavirus DNA testing. *BJOG : an international journal of obstetrics and gynaecology* 2012; **119**(6): 699-709.

21. Zhao ZM, Pan XF, Lv SH, et al. Quality of life in women with cervical precursor lesions and cancer: a prospective, 6-month, hospital-based study in China. *Chinese journal of cancer* 2014; **33**(7): 339-45.

22. WHO. Global strategy to accelerate the elimination of cervical cancer as a public health problem. 2020. <https://www.who.int/publications/i/item/9789240014107> (accessed June 1, 2022).

23. Burger EA, Smith MA, Killen J, et al. Projected time to elimination of cervical cancer in the USA: a comparative modelling study. *The Lancet Public health* 2020; **5**(4): e213-e22.

24. Haacker M, Hallett TB, Atun R. On discount rates for economic evaluations in global health. *Health policy and planning* 2020; **35**(1): 107-14.

25. National Bureau of Statistics of China. National Data. <http://data.stats.gov.cn/english/> (accessed June 1, 2022).

26. National Bureau of Statistics of China. China Population and Employment Statistics Yearbook 2016. Beijing: China Statistics Press; 2017.

27. National Institutes for Food and Drug Control. 2016 China Health Statistics Yearbook. Beijing: Peking Union Medical College Press; 2016.

28. He J. 2018 China Cancer Registry Annual Report. Beijing, 2019.

29. United Nations Department of Economic and Social Affairs Population Division. World Population Prospects: The 2017 Revision. 2017. <https://population.un.org/wpp/Download/Standard/Fertility/> (accessed June 1, 2022).

30. United Nations Department of Economic and Social Affairs Population Division. World Urbanization Prospects: The 2018 Revision. 2018. <https://population.un.org/wup/Download/> (accessed June 1, 2022).

31. Gu CL, Guan WH, Liu HL. Chinese urbanization 2050: SD modeling and process simulation. *Science China Earth Sciences* 2017.

32. UN Development Programme China, Development Research Center of the State Council of China. China national human development report 2016: social innovation for inclusive human development. 2016. <http://hdr.undp.org/sites/default/files/2016_human_development_report.pdf> (accessed June 1, 2022).

33. Bao H, Zhang L, Wang L, et al. Significant variations in the cervical cancer screening rate in China by individual-level and geographical measures of socioeconomic status: a multilevel model analysis of a nationally representative survey dataset. *Cancer medicine* 2018; **7**(5): 2089-100.

34. Bao H, Wang L, Wang L, et al. Study on the coverage of cervical and breast cancer screening among women aged 35-69 years and related impact of socioeconomic factors in China, 2013. *Chinese Journal of Epidemiology* 2018; **39**(2): 208-12.
